# PBMC-derived RNA signatures show impaired ability to regulate immune responses in women developing preeclampsia^†^

**DOI:** 10.1093/biolre/ioag143

**Published:** 2026-07-09

**Authors:** Monica Frøystad, Thor Ueland, Marie Cecilie Paasche Roland, Tove Lekva

**Affiliations:** Research Institute of Internal Medicine, Oslo University Hospital, Oslo, Norway; Faculty of Medicine, University of Oslo, Oslo, Norway; Research Institute of Internal Medicine, Oslo University Hospital, Oslo, Norway; Faculty of Medicine, University of Oslo, Oslo, Norway; Department of Medical Biochemistry, Oslo University Hospital, Oslo, Norway; Research Institute of Internal Medicine, Oslo University Hospital, Oslo, Norway

**Keywords:** preeclampsia, PBMCs, lncRNA, notch signaling, immune activation

## Abstract

Preeclampsia is a serious pregnancy complication with unclear etiology, making early detection challenging but imperative to improve prognosis. We here examine the potential roles of protein-coding and long non-coding (lnc) RNA from peripheral blood mononuclear cells (PBMCs) in the development of preeclampsia by examining women during pregnancy and 5 years following pregnancy. We isolated PBMCs from women with preeclampsia (*N* = 37) and normal pregnant controls (*N* = 215) at 22–24 and 36–38 weeks of gestation, and 5-year follow-up. We performed RNA sequencing at all time points for *N* = 7 normal pregnant controls and *N* = 9 women with preeclampsia and explored data manually and using bioinformatics analyses. Spearman correlation was used between lncRNA and preeclampsia-associated markers, while Notch-related RNAs were measured by qPCR. We found dysregulated RNAs at all time points, mostly at 36–38 weeks (491 upregulated, 155 downregulated). Most downregulated RNAs were lncRNA and these correlated positively with P-selectin and negatively with sFlt-1 and myeloperoxidase from the maternal circulation. Overrepresentation analyses and manual data exploration at 36–38 weeks revealed increased activation of adaptive immune functions and dampening of innate immune response, including Notch signaling. *NOTCH2NLA/B/C* was decreased at 22–24 weeks in the total cohort. In conclusion, circulating protein-coding and lncRNA from women developing preeclampsia broadly indicate enhanced adaptive immune cell activation but dampened innate immune responses. The association of downregulated lncRNAs with markers of endothelial dysfunction and platelet activation may support impaired immune regulation in these women. The function of circulating lncRNAs in women with preeclampsia warrants further study.

## Teaser

Women with preeclampsia have aberrant immune regulation during pregnancy, as shown by transcriptomic data from circulating peripheral blood mononuclear cells.

## Graphical abstract

**Graphical Abstract:**
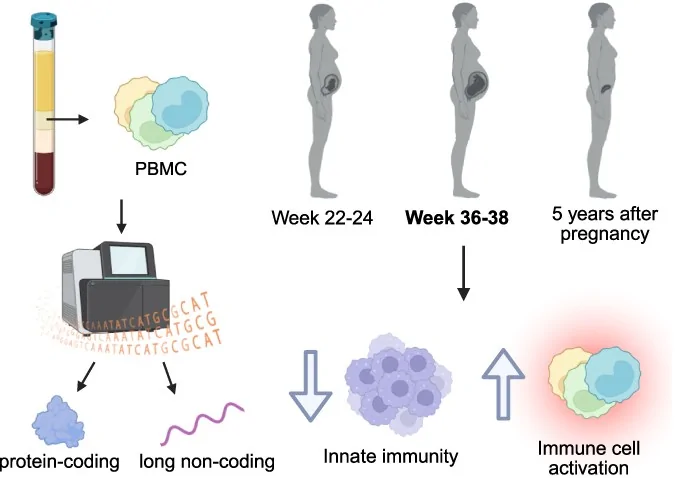

## Introduction

Preeclampsia is a potentially severe complication of pregnancy which affects 2–8% of pregnancies worldwide, making it a leading cause of maternal and fetal perinatal morbidity and mortality [1, 2]. The disease is characterized by development of hypertension after 20 weeks of gestation along with proteinuria or other signs of end-organ damage [3, 4]. Women with a history of preeclampsia are at increased risk for future cardiovascular disease (CVD), and infants of these women have increased risk of obesity, diabetes, and hypertension [5–10].

Preeclampsia is commonly categorized as either early-onset, with diagnosis or delivery <34 weeks of gestation, or late-onset at or after 34 weeks of gestation. The two subtypes of preeclampsia are thought to have different underlying pathological mechanisms, with early-onset more associated with syncytiotrophoblast stress and defective spiral artery remodeling in the placenta [11, 12]. Late-onset preeclampsia is more associated with maternal factors and is characterized by vascular endothelial stress in the mother, in addition to a weaker association with the common preeclampsia biomarkers soluble Flt-1 (sFlt-1) and placental growth factor (PlGF) [6, 13–15].

Immune cell activation is disturbed in preeclampsia with excessive activation of leukocytes involving neutrophils, monocytes, T cells, and NK cells, leading to augmented release of inflammatory proteins and anti-angiogenic factors [16]. This enhanced systemic inflammation may impair placental development, e.g., trophoblast invasion, as well as vascular remodeling, promoting endothelial dysfunction, hypertension, and maternal complications [16]. Furthermore, the placenta may release a high quantity of extracellular vesicles (EVs) containing both coding and long non-coding (lnc) RNAs that may be taken up by maternal immune cells and regulate their functions [17]. Secretion of EVs and their uptake by circulating immune cells is increased in preeclampsia [18], potentially representing a vicious circle at the maternal–fetal interface with sustained systemic inflammation and endothelial stress. Indeed, we and others have showed that lncRNAs may represent early biomarkers in preeclampsia, although these studies have typically been performed with qPCR on selected targets [19–21].

RNA sequencing (RNAseq) is a powerful tool for characterization of the transcriptomes of cells and tissues, providing insights into the expression of both protein-coding and non-coding RNAs [22]. Moreover, an abundance of studies performed RNAseq on placental tissue in preeclampsia and through bioinformatic deconvolution or through single-cell resolution, identified a role and provided insight into immune dysregulation in preeclampsia pathogenesis [23, 24]. Fewer studies have performed RNAseq on maternal immune cells early in pregnancy, preceding diagnosis and with temporal sampling, as opposed to microarray studies assessing whole blood or peripheral blood mononuclear cells (PBMCs) [25–27]. Indeed, preeclampsia was already understood to be characterized by an excessive maternal systemic inflammatory response, with activation of both the innate and adaptive arms of the immune system, more than 2 decades ago [28, 29].

In this exploratory study, we use bulk RNAseq to explore differences in the transcriptomic profiles of PBMCs from normal pregnant women and women with late-onset preeclampsia from the Norwegian STORK cohort, where we have samples from antenatal visits as well as 5-year follow-up. Our aim is to uncover novel protein-coding or lncRNAs in the circulation of women with preeclampsia during and after pregnancy, which may play crucial roles in the development of preeclampsia and thus provide new avenues for elucidating its etiology. In addition, some RNAs were chosen and further validated by qPCR in a larger cohort of women developing preeclampsia, as well as normal pregnancies.

## Methods

### Cohort

The population in this study consists of follow-up examinations of 300 women from the Norwegian STORK cohort, as described elsewhere [30]. Briefly, STORK consists of 1031 women who gave birth at Oslo University Hospital, Rikshospitalet, between 2001 and 2008. Exclusion criteria included pre-gestational diabetes and severe chronic conditions [30]. The women had study-related visits at 14–16 weeks, 22–24 weeks, 30–32 weeks, and 36–38 weeks of gestation, with 300 participants also examined 5 years following the index pregnancy [31]. Of the follow-up women, 215 were normotensive controls and 10 of them had developed preeclampsia at index pregnancy in the STORK study, while women who had gestational diabetes mellitus (GDM) at index pregnancy using WHO 2013 diagnostic criteria were excluded from this study. In addition, we used data from 27 women who had preeclampsia at index pregnancy in STORK but who were not examined at 5-year follow-up, resulting in a total of *N* = 215 normal pregnant controls and *N* = 37 women with preeclampsia used for validation analyses. Using the 300 women from the 5-year follow-up study, we created an additional validation cohort by separating participants by preeclampsia diagnosis before index pregnancy or between index pregnancy and the follow-up study (*N* = 29), and women who had no history of preeclampsia (*N* = 271).

Preeclampsia was diagnosed using criteria in the 2001–2008 period, namely hypertension with blood pressure (BP) ≥140/90 mmHg and proteinuria at total protein/creatinine ratio >30 or +1 on a urine dipstick. Most cases of preeclampsia were late-onset preeclampsia diagnosed after gestational week 34, while 3 cases were early-onset preeclampsia. One case of early-onset preeclampsia was included in RNA sequencing at 22–24 weeks and at 5-year follow-up.

Clinical examinations were conducted according to the principles of the Declaration of Helsinki. The study was approved by the Regional Committee for Medical Research Ethics (REK) of Southern Norway in Oslo, Norway (REK# S-01191a, S-07392a).

### Uterine artery pulsatility index

Ultrasound examinations were done using the same equipment for all participants (Acuson Aspen, Mountain View, CA). The mean uterine artery pulsatility index (PI) was obtained by bilateral Doppler flow velocity measurements using an abdominal approach with measurements performed close to the crossing of the external iliac arteries, keeping the insonation angle as low as possible. PI is defined as the difference between the peak systolic flow and minimum diastolic flow velocity, divided by the mean velocity recorded throughout the cardiac cycle. The formula for PI = (peak systolic velocity-end diastolic velocity)/mean flow velocity. Here, the PI was calculated as the mean of three heart cycles on each side. Some of the uterine artery PI data was missing (approximately 10 preeclampsia and 40 normal pregnancies) in the larger cohort (Table S8).

### Biochemical analysis

Peripheral venous blood was drawn in tubes with citrate additive between 07:30 and 08:30 a.m. following an overnight fast. Tubes were centrifuged for 25 min at 3000 g at 4°C, and plasma was collected and stored at −80°C. Levels of sFlt-1 (catalog #DY321B), PlGF (#DY264), myeloperoxidase (MPO) (#DY3174), B-cell activating factor (BAFF) (#DY124-05), and P-selectin (#DY137) were measured by enzyme immune-assay (ELISA) as previously published [21, 32]. CD14 (#DY383) and Thrombomodulin (#DY3947) were measured by ELISA using antibodies from R&D Systems (Minneapolis, MN), while for von Willebrand factor, we used antibodies from Dako Cytomation (Norway), as described elsewhere [33]. Duplicates were measured and analyzed using CyBi SELMA (CyBio, Jena, Germany) for pipetting, an EL406 washer/dispenser (Biotek, Winooski, VT) and a Synergy H1 microplate reader from Biotek. Fasting plasma levels of advanced oxidation protein products (AOPP) were measured in duplicate using 5 μL plasma by absorbance readings using a kit from Abcam (#ab242295, Cambridge, UK). Fasting plasma beta-galactosidase was measured in duplicate by fluorescence, as previously published [34].

### Blood collection and extraction of RNA

Blood was drawn in BD Vacutainer cell preparation tubes (CPT) tubes (8 mL) with sodium citrate additive (Becton Dickinson Vacutainer Systems, Franklin Lakes, NJ) following an overnight fast at gestational weeks 22–24 and 36–38, as well as 5 years following pregnancy. CPT tubes were centrifuged at room temperature in a swing-out head horizontal rotor for 20 min at 1800 g with no brake to establish a mononucleated cell layer. Maternal plasma was collected from the top layer in the CPT tube, and the cell layer was collected separately and stored at −80°C.

Isolation of total RNA was performed with two different kits due to changing instrumentation in the lab. The MagNA Pure Isolation Kit (Roche Life Science, Penzberg, Germany) was used at 22–24 weeks and MagMAX Isolation Kit (Applied Biosystems, Carlsbad, CA) at 36–38 weeks and at 5-year follow-up, as previously published [35].

The RNA used for sequencing from week 36–38 was cleaned up using RNeasy PowerClean Pro Cleanup Kit (Qiagen, Hilden, Germany). For samples at 5-year follow-up, PBMCs were thawed and put in QIAzol (Qiagen) before isolating RNA using an miRNeasy Kit (Qiagen) with DNase digestion using RNase-Free DNase Set (Qiagen).

RNA quality and concentration were measured using the Nanodrop ND-1000 Spectrophotometer (Thermo Fisher Scientific Inc., Waltham, MA), and the integrity of RNA was examined using a 2100 Bioanalyzer from Agilent (Agilent Technologies, Santa Clara, CA).

### Quantitative real-time polymerase chain reaction

PBMC-derived RNA from 22–24 weeks, 36–38 weeks, and 5-year follow-up from all 300 women from the 5-year follow-up study, as well as samples during pregnancy from women who had preeclampsia at index pregnancy but were not examined at 5-year follow-up (*N* = 27), was used in qPCR following cDNA synthesis. All cDNA was synthesized simultaneously from 100 ng uncleaned RNA using the High-Capacity cDNA Archive Kit from Applied Biosystems (Carlsbad, CA). Quantitative polymerase chain reaction (qPCR) was performed in duplicates using PerfeCta SYBR Green PCR FastMix ROX (Quantabio, Beverly, MA) with standard cycling conditions of 3 min initial denaturation at 95°C, 15 s elongation at 95°C, and extension of 60 s at 60°C for 40 cycles, as indicated in the SYBR Green FastMix product protocol [36]. Sequence-specific oligonucleotide primers for qPCR were designed with NCBI Primer-BLAST for *NOTCHNLA/B/C* using a NCBI reference sequence for *NOTCH2NLC* (NM_001364012.2), yielding forward primer 5′-CTTTGAAGCAGGAGGAGGGG-3′ and reverse primer 5′- CATAGCCATCTCGACACATGC-3′.

Data from qPCR were preprocessed in CFX Maestro Software (Bio-Rad Laboratories, Hercules, CA), where RFU was set in the exponential phase, and duplicates with non-exponential quantification curves or no amplification were removed. Starting quantities (SQ) for each target were averaged across duplicates and divided by the average mean of the reference RNAs *ACTB* and *GAPDH*. Primers for *ACTB* (NM_001101.3) were forward 5′-AGGCACCAGGGCGTGAT-3′ and reverse 5′-TCGTCCCAGTTGGTGACGAT-3′, while *GAPDH* (NM_00-1256799.1/NM_002046.4/NM_017008.3) primers were forward 5′-CCAAGGTCATCCATGACAACTT-3′ and reverse 5′-AGGGGCCATCCACAGTCTT-3′.

### RNA sequencing

Our discovery cohort consisted of RNA samples from normal pregnant controls (*N* = 7) and preeclampsia (*N* = 9) from the total cohort (*N* = 215 controls, *N* = 37 preeclampsia) and samples were matched by maternal age, body mass index (BMI), and sex of the baby. Sample size was reduced at 36–38 weeks due to poor sample quality following rRNA depletion and missing samples (*N* = 6 normal pregnant controls and *N* = 4 preeclampsia). Despite longitudinal samples, longitudinal analyses were avoided for RNAseq data due to differences in RNA isolation protocols at each time point. Criteria for normal pregnant controls included fasting, 1 h and 2 h glucose blood sugar within normal range, ≤5.1, ≤10, and ≤8.5, respectively, after oral glucose tolerance test at weeks 30–32, excluding GDM using criteria from WHO 2013. rRNA depletion, sample preparation, and RNAseq was performed by the Norwegian Sequencing Centre using the QiaSeq FastSelect-rRNA HMR Kit (Qiagen) and the TruSeq Stranded Total RNA Library Prep Human (Illumina, San Diego, CA). RNAseq was run on a 2/4 NovaSeq S4 flow cell with 150 bp paired end reads on a NovaSeq 6000 Sequencing System (Illumina).

### Preprocessing of RNA sequencing data

RNAseq raw data was preprocessed and quality controlled by Helse Sør-Øst Bioinformatics Core Facility at Oslo University Hospital using R v4.2.1. All fastq files were evaluated using FastQC v0.11.9 in their raw and trimmed states [37]. Optimization of alignment and mapping was achieved with TrimGalore v0.0.6 [38] and fastq files were submitted as pairs. Briefly, the reads were quality trimmed (Phred ≥20) before searching for Illumina adapters based on a 13 bp sequence present in standard Illumina paired-end sequencing adapters, removing adapters with ≥1 bp overlap. Reads ≥20 nt were aligned to the human genome (GRCh38 version 43, https://www.gencodegenes.org/human/) using STAR v2.7.5a [39]. STAR was run with default parameters with output files BAM (SortedByCoordinate) and GeneCount table. Estimated percentages of remaining rRNA following rRNA depletion were evaluated for the fastq files with BBmap v38.86 together with a human rDNA repetitive element reference [40]. BAM files from selected samples were run through QoRTs v1.3.6 to look at library complexity and 3′ and 5′ degradation patterns, see selected plots in Supplemental Figure S1 [41]. VariancePartition v1.26.0 was used to explore contributors to variation of biological and technical origin in all samples using the count matrix adapted from STAR output (Supplemental Figure S1) [42]. Here, we used factors like RNA isolation method, participant group, the log output from bbduk (rRNA content), and statistics from MultiQC v1.9 (uniquely mapped, total reads, duplication), aiming to find any significant batch effects [43].

### Differential gene expression analysis

Differential gene expression (DGE) analysis and most downstream analyses of the RNAseq data were performed in R studio v2023.03.1 (build 446) using R v4.2.3. We performed DGE analysis of preeclampsia vs. normal pregnant controls at 22–24 weeks, 36–38 weeks, and at 5-year follow-up using raw count data for each time point, respectively, and DESeq2 v1.38.3 with default settings [44]. Principal component analysis (PCA) was performed using raw counts transformed with variance-stabilizing transformation (VST) and visualized using plotly v4.12 in R (Supplemental Figure S2). The interactive PCA plot can be accessed in Zenodo (doi:https://doi.org/10.5281/zenodo.20830108). Volcano plots were created using EnhancedVolcano v1.16.0 [45]. Candidates with high relative expression level, log_2_ fold-change (FC) cutoff at |1|, and false discovery rate (FDR) <0.05 following Benjamini–Hochberg *P*-value adjustment were selected for validation by polymerase chain reaction (PCR) in the entire cohort (*N* = 215 controls, *N* = 37 PE). RNA identifiers were converted from Ensembl gene ID versions to Ensembl gene IDs using GeneStructureTools v1.18.0 and annotated offline using org.Hs.eg.db v3.16.0 and AnnotationDbi v1.60.2 [46–48]. More detailed RNA biotype annotation (Figure 1) was achieved with the online tool iDEP (integrated Differential Expression and Pathway analysis) Enrichment feature under the “DEG2” tab in v2.01 [49].

**Figure 1:**
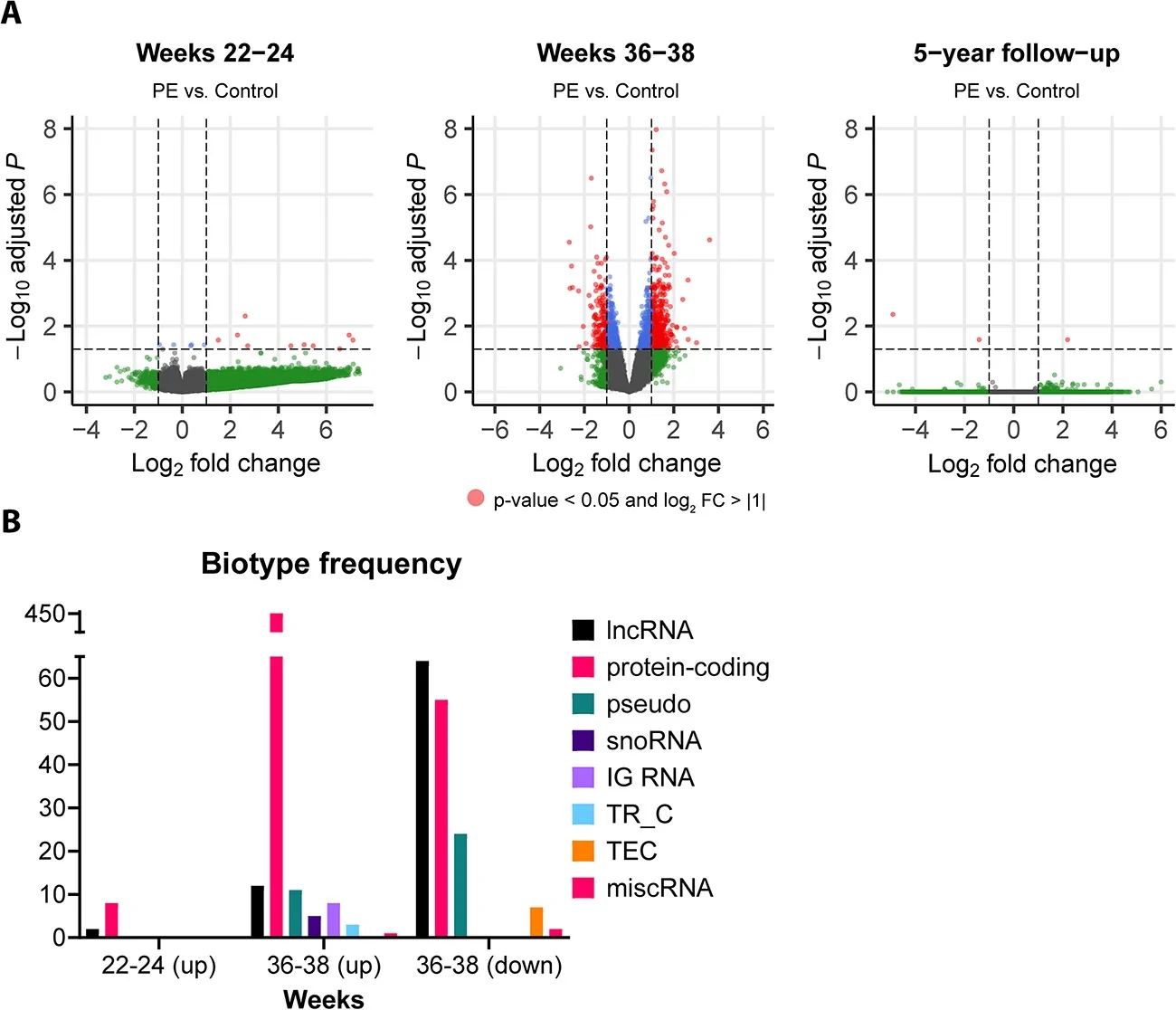
Differential gene expression analysis. (A) Volcano plots visualizing differential gene expression analysis of RNA sequencing data from PBMCs from preeclampsia compared to normal pregnant controls at three different time points, including twice during pregnancy at 22–24 weeks (*N* = 7 controls, *N* = 9 preeclampsia) and 36–38 weeks (*N* = 6 controls, *N* = 4 preeclampsia), as well as at 5-year follow-up (*N* = 7 controls, *N* = 9 preeclampsia). The log_2_ FC cutoff = |1| and FDR adjusted *P*-value cutoff was set to 0.05. Each dot represents one RNA and adjusted *P*-value below cutoff and log_2_ FC above cutoff represents significantly differentially expressed RNAs. PE = preeclampsia, NS = not significant. (B) Bar plot showing the frequency of each biotype for differentially expressed RNAs at 22–24 weeks and at 36–38 weeks in preeclampsia vs normal pregnant controls (red dots in volcano plot). Each bar represents one type of RNA and bars are grouped by time point with the direction of regulation in brackets (up or down). Few RNAs were regulated at 5-year follow-up and are not shown here. lnc = long non-coding, pseudo = pseudogenes, snoRNA = small nucleolar RNA, IG = immunoglobulin, TR_C = T cell receptor constant chain, TEC = To be Experimentally Confirmed, miscRNA = miscellaneous RNA.

### Overrepresentation analyses of differentially expressed RNAs

We performed overrepresentation analyses at each time point using statistically significant (FDR-adjusted *P*-value <0.05) RNAs either upregulated (log_2_ fold-change >1) or downregulated (log_2_ fold-change <−1) in preeclampsia following DGE analysis. We performed Reactome pathway enrichment analysis and Gene Ontology (GO) biological process enrichment analysis of dysregulated RNAs using the “Enrichment” tab under “DEG2” in iDEP v2.01 [49]. We retrieved NOTCH-pathway RNAs from Kyoto Encyclopedia of Genes and Genomes (KEGG) (hsa04330), as well as other NOTCH-related RNAs, and explored their expression levels at each time point using VST-transformed counts scaled across all samples for each RNA and visualized with ComplexHeatmap v2.22.0 [50].

### Deconvolution analysis

We performed deconvolution analysis to elucidate the relative amount of each cell type in every PBMC sample (*N* = 16) at each time point. Briefly, this method uses previously characterized microarray and RNAseq data from PBMCs as a reference point for matching RNAs and their relative proportions to cell types. We used the online interface for CIBERSORTX (https://cibersortx.stanford.edu/) and the built-in “LM22” PBMC signature matrix, run with B-mode batch-correction enabled and in relative mode with 100 permutations [51]. We evaluated differences in cell type proportions between normal pregnant controls and preeclampsia using a simple *t-*test with 2-tailed *P*-values and assuming non-equal variance. Cell type proportions within each group at different time points were visualized with ComplexHeatmap v2.22.0 with data scaled across all samples for each cell type [50].

### Statistical analysis of clinical variables

We compared participant characteristics between women with preeclampsia and normal pregnant women at each time point for the total cohort and the RNAseq cohort, as well as the cohort with history of preeclampsia vs. no history. Comparison of clinical variables was performed using Mann–Whitney U or *t-*test for continuous variables, depending on distribution, and Chi-square test for categorical variables unless *N* < 5, where we used Fisher’s exact test [52]. Normally distributed data are expressed as mean ± SD and skewed data as median (25th and 75th percentiles). Patient characteristics were analyzed using tableone v0.13.2 in R [52].

Statistical analyses of qPCR data were performed using IBM SPSS Statistics v3.1 (Chicago, IL) for Windows. To assess relative *NOTCH2NLA/B/C* expression between normal pregnant controls and women with preeclampsia, we used generalized linear mixed model analysis with gamma distribution and a log link function, with subject (participant ID) as random effect, and maternal age, BMI, time, and preeclampsia diagnosis, as well as interaction between time and preeclampsia diagnosis, as fixed effects. The model was adjusted for maternal age and BMI, but not for blood pressure or angiogenic factors sFlt-1 and PlGF, as these were considered disease-related outcomes. We used linear regression analysis to assess *NOTCH2NLA/B/C* expression at 5-year follow-up (dependent variable) in women with history of preeclampsia vs. no history, accounting for maternal age and BMI. We performed Spearman correlation analyses of *NOTCH2NLA/B/C* expression and various clinical variables, as well as markers associated with preeclampsia at each time point. We also investigated dysregulated lncRNAs at 36–38 weeks and their association with selected clinical markers using Spearman correlation analysis. Two-tailed *P*-values are given and were considered significant at ≤0.05.

## Results

### Participant characteristics

A summary of participant characteristics, including maternal age, parity, gestational age, sex of the baby, BMI, blood pressure, and angiogenic factors can be found in Table 1 for RNAseq samples at 22–24 weeks and 5-year follow-up (*N* = 7 controls, *N* = 9 preeclampsia), as well as 36–38 weeks (*N* = 6 controls, *N* = 4 preeclampsia). The samples were matched for age, BMI, and fetal sex between groups. sFlt-1 levels were elevated in preeclampsia at 22–24 weeks (*P* = 0.05) and at 36–38 weeks (*P* = 0.025), while a trend (*P* = 0.064) was seen for a higher sFlt-1/PlGF ratio at 36–38 weeks. No significant differences were found at this time point although sFlt-1 levels were higher in preeclampsia (*P* = 0.088).

**Table 1:** Participant characteristics of the patient samples submitted for RNA sequencing (*N* = 16)

Characteristics	Normal pregnant controls (*N* = 7)	Preeclampsia (*N* = 9)	*P*-value	Normal pregnant controls (*N* = 6)	Preeclampsia (*N* = 4)	*P*-value
	Gestational weeks 22–24 and 5-year follow-up			Gestational weeks 36–38		
Age (mean (SD))	30 [28, 32]	29 [27, 33]	0.873	30 [27, 31]	26 [25, 29]	0.334
Primiparous n (%)	2 (28.6)	7 (77.8)	0.126	1 (16.7)	3 (75.0)	0.190
Sex of the baby: female (%)	6 (85.7)	5 (55.6)	0.455	5 (83.3)	2 (50.0)	0.500
BMI (kg/m^2^)^*^						
Weeks 14–16	23.6 [22.0, 24.3]	23.1 [20.5, 25.9]	0.817	23.7 [23.0, 24.5]	24.6 [21.6, 25.2]	1.000
Weeks 22–24	25.1 [23.0, 25.9]	23.8 [21.7, 26.0]	0.643	25.2 [24.2, 26.1]	25.2 [22.6, 25.2]	0.439
Weeks 36–38	26.4 [24.8, 27.6]	27.6 [25.0, 28.1]	0.668	26.9 [25.5, 27.7]	27.4 [25.5, 27.8]	1.000
5-year follow-up	23.6 [20.9, 24.1]	23.1 [20.2, 25.7]	0.634			
Systolic BP (mmHg)^*^						
Weeks 22–24	120 [110, 120]	110 [104, 120]	0.375			
Weeks 36–38	115 [113, 123]	125 [120, 140]	0.246	118 [115, 124]	123 [120, 126]	0.328
5-year follow-up	116 [105, 120]	110 [105, 110]	0.412			
Diastolic BP (mmHg)^*^						
Weeks 22–24	70 [70, 70]	70 [60, 80]	0.951			
Weeks 36–38	70 [70, 78]	80 [75, 90]	0.165	73 [70, 79]	80 [78, 81]	0.211
5-year follow-up	70 [67, 70]	70 [60, 75]	0.504			
Uterine artery PI^*^						
Weeks 22–24	0.81 [0.64, 1.10]	1.10 [0.89, 1.42]	0.262			
Weeks 36–38	0.79 [0.68, 0.99]	0.87 [0.79, 0.98]	0.873	0.82 [0.64, 1.05]	0.79 [0.79, 0.98]	0.881
sFlt-1 (ng/mL)^*^						
Weeks 22–24	1.1 [0.8, 1.5]	2.2 [1.3, 3.4]	0.050			
Weeks 36–38	2.2 [2.0, 2.7]	6.7 [5.5, 11.4]	0.025	2.3 [2.0, 2.9]	9.0 [5.6, 11.3]	0.088
5-year follow-up	0.4 [0.3, 0.7]	0.5 [0.3, 0.7]	0.491			
PlGF (pg/mL)^*^						
Weeks 22–24	142 [92, 177]	160 [109, 250]	0.560			
Weeks 36–38	184 [99, 257]	44 [26, 96]	0.142	219 [92, 259]	62 [37, 149]	0.522
5-year follow-up	8 [5, 25]	7 [5, 29]	0.825			
sFLlt1/PlGF ratio^*^						
Weeks 22–24	8.29 [6.13, 10.58]	9.70 [4.36, 43.76]	0.874			
Weeks 36–38	11.88 [9.75, 25.88]	184.85 [64.80, 462.11]	0.064	11.02 [9.54, 29.65]	174.48 [65.47, 362.87]	0.286
5-year follow-up	38.00 [19.46, 110.00]	62.00 [51.43, 98.28]	0.791			

^*^(median [25th, 75th percentile]), BP = blood pressure.

### Differential gene expression analysis

RNAseq and subsequent DGE analysis revealed dysregulated RNAs in PBMCs from women with preeclampsia compared to normal pregnant controls at 22–24 weeks (10 upregulated), 36–38 weeks (491 upregulated and 155 downregulated), and 5-year follow-up (1 upregulated and 2 downregulated). The volcano plots in Figure 1A illustrate the differentially expressed RNAs with log_2_ FC cutoff at |1| and FDR-adjusted *P*-value <0.05 for each time point. Complete lists of significantly regulated RNAs at each time point are shown in Tables S1–S4.

Biotype distributions of the dysregulated RNAs are illustrated in Figure 1B. At 36–38 weeks, most upregulated RNAs were protein-coding (*n* = 449, 91.4%), a few were lncRNA (*n* = 12, 2.4%) and pseudogenes (*n* = 12, 2.4%), while downregulated RNAs were mostly lncRNA (*n* = 64, 41.3%), followed by protein-coding RNA (*n* = 55, 35.5%) and a high share of pseudogenes (*n* = 24, 15.5%).

### Overrepresentation analyses and NOTCH-related RNAs at 36–38 weeks of gestation

We performed overrepresentation analyses for up- and downregulated RNAs at 36–38 weeks and lists of RNAs involved in each pathway term can be found in Supplemental Tables S5 and S6. In both GO enrichment and pathway analysis, shown in Figure 2A and B, respectively, we found many general terms for biological, cellular, or signaling processes such as several terms involved in transcription, translation, and growth processes for upregulated RNAs. For downregulated RNAs, GO enrichment analysis revealed several virus response and innate immunity terms, while pathway analysis specified immune signaling pathways such as Toll-like receptor cascades. Importantly, we found a specific term for “Positive regulation of Notch signaling pathway” in the otherwise generalized GO terms for downregulated RNAs. We subsequently explored the expression levels of RNAs involved in this term (*NOTCH2NLA, NOTCH2NLB*, and *NOTCH2NLC*) and some of their co-evolving RNAs [53] (*NBPF10, NBPF14*, and *NBPF19*, respectively), as well as NOTCH signaling pathway-related RNAs (*TLE5* and KEGG pathway hsa04330) at 36–38 weeks, as shown in Figure 2C. Figure 2D highlights differentially expressed NOTCH-related RNAs, of which *DTX1, DTX3, MFNG*, and *TLE5* were upregulated and *NOTCH2NLA, NOTCH2NLB, NOTCH2NLC*, and *NBPF19* were downregulated in preeclampsia compared to normal pregnant controls at 36–38 weeks of pregnancy. We chose to validate *NOTCH2NLA, NOTCH2NLB*, and *NOTCH2NLC* in our larger cohort (*N* = 252) using qPCR, as these had high expression levels with log_2_ FC (FDR adjusted *P*-value) of −1.1 (*P* < 0.003), −1.5 (*P* < 0.001), and −1.7 (*P* < 0.001), respectively (see below).

**Figure 2:**
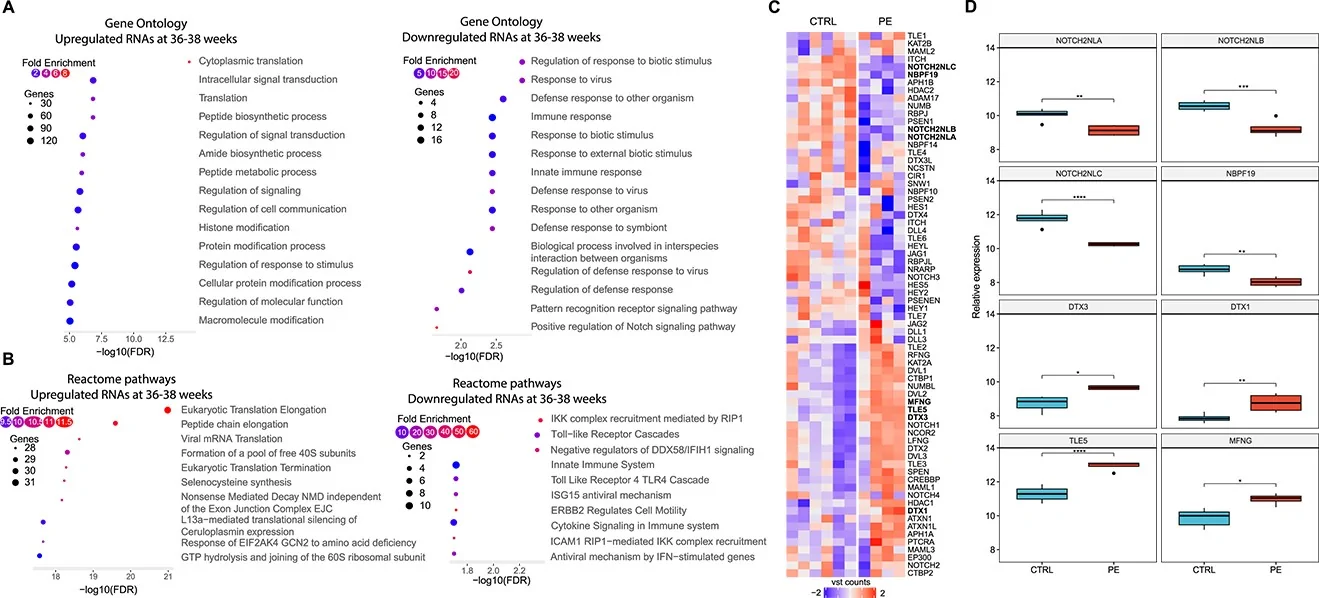
Overrepresentation analyses and NOTCH-related RNAs. (A) Dot plots showing the top 15 biological process (BP) GO terms and (B) top 10 terms from Reactome pathway analysis of up- and downregulated RNAs, respectively, at 36–38 weeks (*N* = 6 controls, *N* = 4 preeclampsia). Genes refers to how many RNAs are involved in each term, −log_10_(FDR) to the Benjamini–Hochberg adjusted *P*-value with cutoff 0.05 and the fold enrichment shows the degree of overrepresentation of the pathway term in our data compared to all protein-coding genes. (C) Heatmap showing relative expression of NOTCH-related RNAs between women with preeclampsia (PE) and normal pregnant controls (CTRL) at weeks 36–38. Significantly dysregulated RNAs are highlighted in bold letters. (D) Boxplots highlighting the up- or downregulated NOTCH-related RNAs with log_2_ fold change cutoff at |1| and FDR-adjusted *P*-value <0.05. Expression levels are shown using VST normalized counts. CTRL = normal pregnant controls, PE = preeclampsia, dsRNA = double-stranded RNA. ^*^ = *P* < 0.05, ^**^ = *P* < 0.01, ^***^ = *P* < 0.001, ^****^ = *P* < 0.0001.

We also explored the dysregulated RNAs manually and organized them into groups (Table 2). Interestingly, we found upregulated RNAs coding for T-cell receptors and immune cell activation, as well as many downregulated RNAs encoding mitochondrial pseudogenes at 36–38 weeks.

**Table 2:** Some up- and down-regulated RNAs organized into different groups at gestational week 36–38 (*N* = 6 controls, *N* = 4 preeclampsia)

Upregulated RNAs	
Ribosomes	*RPS2, 3, 5, 7, 12, 14–16, 18–21, RPLP0–2, RPL3, 7A, 8, 13, 13A, 18A, 19, 27–29, 35, 36, RRP9, RPSA, RPS6KB2, MRPS2, NOP53*
Histones	*H3C3, H4C2, H4C4, H4C6, H2AC20, H2AC11, H2BC12, H1–2, H1–3, H1–4, HDAC7*
Immunoglobins	*IGHA1, IGKV3–20, IGHG2, IGLC2, IGLC3, IGKC, IGHG3, IGHD*
T-cell receptor	*TRGC2, TRBC1, TRBC2, ZAP70, CD8A, RUNX3, GATA3, LCK*
Zinc finger proteins	*ZNF683, ZNF48, ZNF512B, ZNF335, ZNF865, ZNF777, ZNF428, ZNF831, ZNF500, ZNF692, ZNF687*
Immune cell activation	*CD2, CD7, CD27, CD5, CD81, TNFRSF25, CST7, NKG7, IL32, IL2RB, IL12RB1, CARMIL2, CARD11, SH2D2A, GPR68, NFATC1, LAG3, ICAM3, ITGB7, ACKR3, TRIM28, CTSW, RASAL3, CD82, ITM2C, GZMH, CXCR3, ITPR3, ORAI1, ANO9, ADGRG1, TBX21*
Immune cell recruitment and migration	*CD82, CD151, ITGB7, ICAM3, RBM38*
VEGF pathway	*VEGFB, FLT4*
Suppress TLR signaling	*SIGIRR*
Cell proliferation and apoptosis	*NPDC1, TMUB1, CDK2AP2, CDC25B, EEF2, LTK, MYBL2, TGFB1I1, ARMC5, NACC1, RBM38, PCBP4, EIF3G, TP53I13, CDK9, FASTK, PPDPF, TCF3, BCL9L, ATP2A3, DUSP2, TMC8, PLEKHF1*
Heat shock protein	*HSPB1*
Oxidative stress	*PRDX2, KEAP1, BCR*
Antiviral defense	*IRF3, TTLL12, TRAF1, TRAF7, RELB, PPP1R15A*
Dampens NFkB signaling	*NFKBIB, LRRC14, NLRC3*
Phagocytosis	*DBN1, APBB1, CNN2, FKBP8, CLU*
**Downregulated RNAs**	
Interferon signaling	*IFI44L, IFIT1, IFI44, IFIT2*
C-type lectin family	*CLEC4D, CLEC12A, CLEC12B, CLEC1A*
Immune response	*LY96, TLR8, EREG, GPR141, UBE2V1, IL15, IRAK3, HERC5, RIG-I*
Protection against oxidative stress	*NME8*
Macrophage-related	*TMEM144, TFEC, LACC1, MS4A7*
Mitochondrial pseudogenes	*MTND5P32, MTND4LP23, MTND4P14, MTND1P11, MTND5P14, MTCYB23, MTCYBP3, MTATP6P11, MTCO1P11, MTCO2P11, MTCO3P11*

### Differentially expressed long non-coding RNAs at 36–38 weeks of gestation

We next explored the significantly dysregulated lncRNAs between preeclampsia and normal pregnant controls at gestational weeks 36–38. Of the 12 upregulated, we found 1 competing endogenous, 1 sense intronic, 1 intronic, 5 antisense, and 4 novel lncRNA, while the 64 downregulated included 1 host gene, 19 sense intronic, 11 intronic, 9 antisense, 3 divergent, 5 intergenic, and 16 novel lncRNA. Briefly, some of the lncRNAs have been shown to be involved in oxidative stress and mitochondrial function (*CYP1B1-AS1*), proliferation and invasion (*LINC01410*), or to have immune-related functions (*POC1B-AS1, MIR181A1HG, BACH1-IT2, RC3H1-IT1, ASAP1-IT2, AOAH-IT1, E2F3-IT1, N4BP2L2-IT2, DAPK1-IT1, CHASERR, MIR223HG, LINC02207, TNFRSF14-AS1*). Also, some lncRNA with unknown functions, *RASA3-IT1, FOXP1-DT*, ENSG00000289310 (sense intronic to *LRRK2*), ENSG00000272807 (antisense to *KANSL1L*), and ENSG00000236194 (sense intronic to *STAT5B*), have complementary coding RNAs significantly differentially expressed but with log_2_ FC <|1| in current study. These have functions involving T cell activation, mitochondrial function, cellular senescence, immune regulation, and growth. Correlating these lncRNA to clinical markers important for preeclampsia development and markers for immune cell activation and endothelial dysfunction, we found many downregulated lncRNAs significantly negatively associated with circulating sFlt-1 (Figure 3), while to a lesser extent associated with circulating PlGF. Also, many lncRNAs were associated with circulating markers of oxidative stress and endothelial dysfunction, like myeloperoxidase, thrombomodulin, and P-selectin (Figure 3). All correlation coefficients for the selected markers can be found in Table S7.

**Figure 3:**
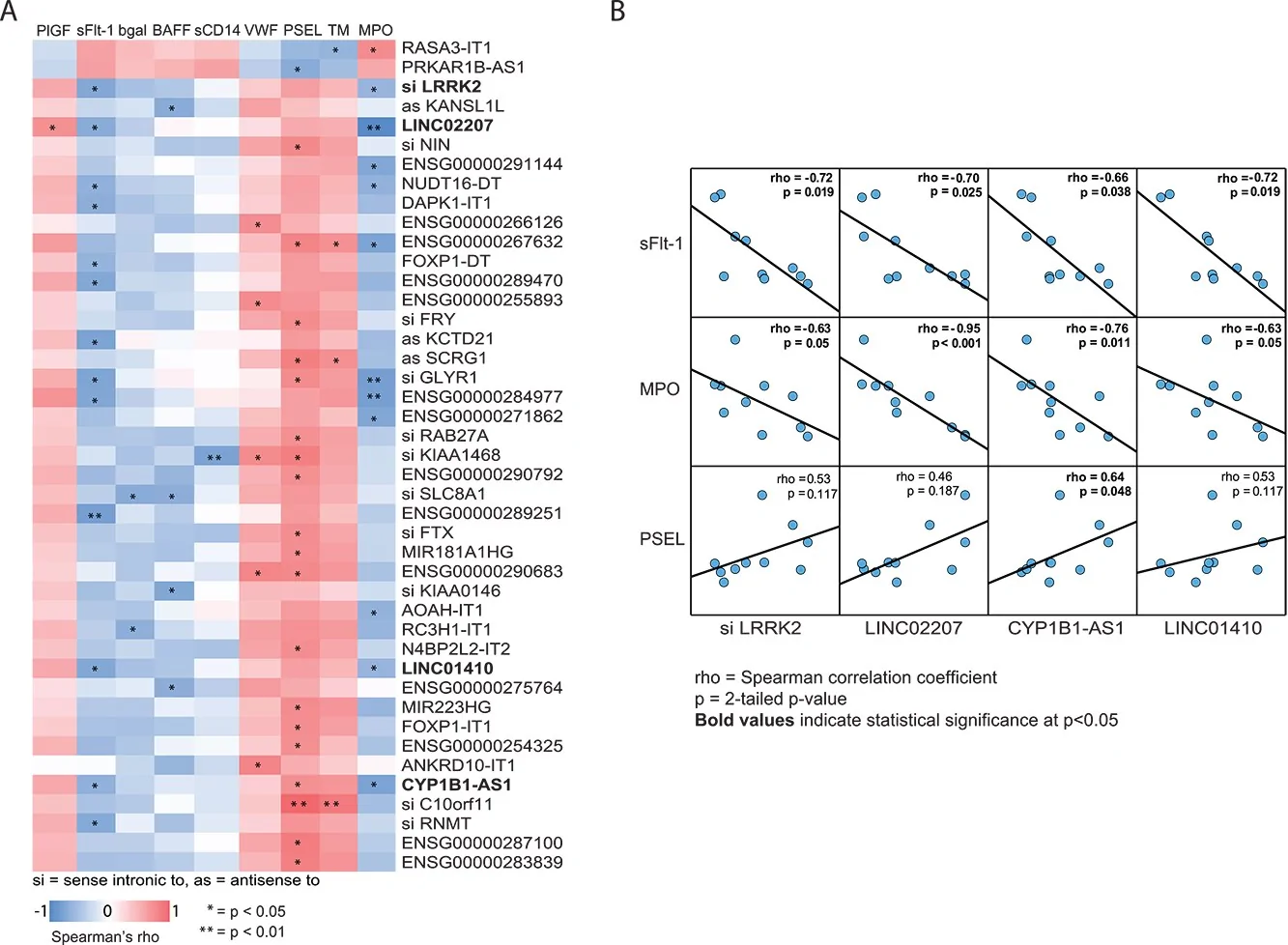
Spearman correlation analyses of lncRNAs dysregulated at 36–38 weeks and clinical markers. (A) Heatmap illustrating correlations between gene expression (normalized count data) of dysregulated lncRNAs at weeks 36–38 (*N* = 6 normal pregnant controls, *N* = 4 preeclampsia) and clinical markers using Spearman correlation analysis with 2-tailed *P*-values. Complete table with Spearman correlation coefficients (rho) and 2-tailed *P*-values can be found in Supplemental Table S7. Blue = negative correlation, Red = positive correlation. PlGF = placental growth factor, bgal = β-galactosidase, BAFF = B-cell activating factor, sCD14 = soluble CD14, VWF = von Willebrand factor, PSEL = P-selectin, TM = thrombomodulin, MPO = myeloperoxidase. ^*^ = *P* < 0.05, ^**^ = *P* < 0.01. (B) A selection of Spearman correlations from (A) illustrated with scatter plots featuring linear regression lines to illustrate correlation direction. Spearman correlation coefficients (rho) and 2-tailed *P*-values (p) are given in the plots. Bold values indicate statistical significance at *P* < 0.05.

### Deconvolution analysis

We performed relative deconvolution analysis to elucidate the estimated proportion of PBMC-derived cell types in each RNAseq sample. Figure 4A shows the distribution of estimated proportions of each cell type across all samples, where monocytes, naïve and memory CD4^+^ T cells, CD8^+^ T cells, and resting NK cells were most abundant at all time points. T regulatory cells and NK cells were more abundant, while activated dendritic cells were less abundant, in women developing preeclampsia compared to normal pregnant controls at 36–38 weeks. At 22–24 weeks and at 5-year follow-up, we found no significant differences in estimated cell proportions between the groups. Finally, expression levels of dysregulated RNAs at 36–38 weeks were explored in the PBMC signature matrix reference (LM22), showing that many upregulated RNAs were specifically abundant in various T cell subsets, NK cells, B cells, and plasma cells (Figure 4B).

**Figure 4:**
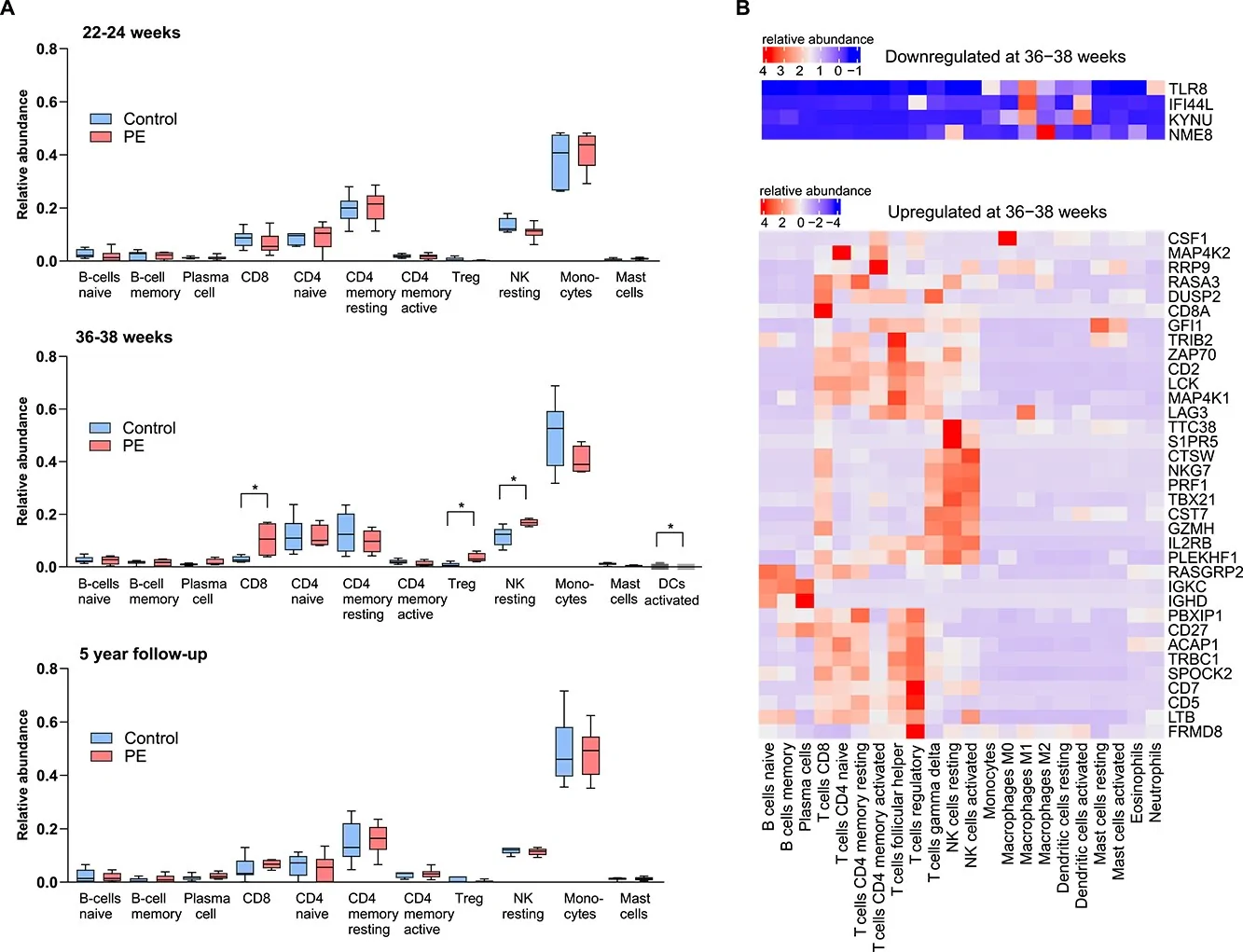
Deconvolution analysis of RNA sequencing data from PBMCs. (A) Boxplots illustrating the distribution of estimated cell type proportions in PBMC samples from women with preeclampsia and normal pregnant controls at 22–24 weeks (*N* = 7 controls, *N* = 9 preeclampsia), 36–38 weeks (*N* = 6 controls, *N* = 4 preeclampsia), and at 5-year follow-up (*N* = 7 controls, *N* = 9 preeclampsia) following deconvolution analysis. Cell types with estimated proportions of zero across most samples are not shown. ^*^ = *P*-value <0.05 (2-tailed *t-*test). (B) Heatmaps showing relative abundance of up- or downregulated RNAs from 36–38 weeks present in the LM22 signature matrix used as reference for the deconvolution analysis and which cell type signatures they are associated with.

### qPCR validation of *NOTCH2NLA/B/C* in a larger cohort

We validated *NOTCH2NLA, NOTCH2NLB*, and *NOTCH-2NLC* combined in our total cohort (*N* = 215 controls, *N* = 37 preeclampsia) using qPCR. The participant characteristics for this cohort are in Table S8, where we found lower maternal age and higher BMI throughout pregnancy in women developing preeclampsia compared to controls. Systolic and diastolic blood pressures, as well as uterine artery PI, were higher during pregnancy in women who developed preeclampsia. In addition, sFlt-1 was elevated and PlGF reduced during pregnancies complicated by preeclampsia. No differences were found between the groups for the selected parameters at 5-year follow-up.

We were unable to design primers targeting each *NOTCH-2NL* RNA specifically due to high similarity and used primers for all three combined (*NOTCH2NLA/B/C*). As shown in Figure 5A, *NOTCH2NLA/B/C* combined was significantly decreased in preeclampsia (*N* = 37) compared to normal pregnancies (*N* = 215) at 22–24 weeks, with a similar trend at 36–38 weeks. Exploratory correlation analyses for *NOTCH2NLA/B/C* and various clinical variables, as well as known preeclampsia-associated markers at each time point (Table S10), revealed that *NOTCH2NLA/B/C* was negatively correlated with circulating plasma levels of β-galactosidase and AOPP at 22–24 weeks and negatively correlated with β-galactosidase at 36–38 weeks. However, all correlations were weak with coefficients <|0.23|. We also investigated whether *NOTCH2NLA/B/C* differed between women with (*N* = 29) or without (*N* = 271) a history of preeclampsia among those who participated in the 5-year follow-up of the STORK study (Table S9) and found no difference (Figure 5B).

**Figure 5:**
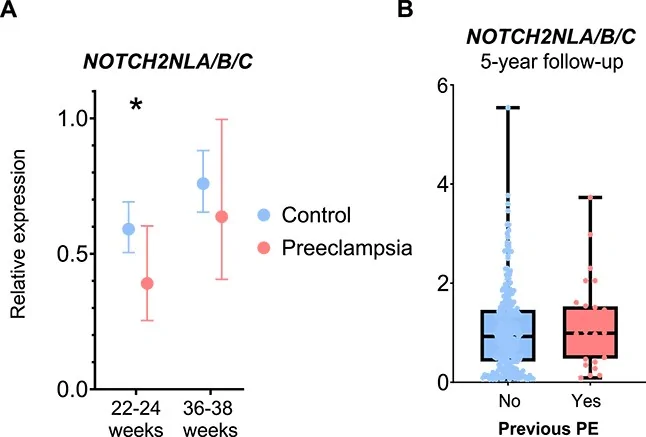
Validation and correlation analyses of *NOTCH2NLA/B/C* at each time point. (A) The relative expression of *NOTCH2NLA/B/C* following qPCR analysis of the total cohort (CTRL = 215, PE = 37) at 22–24 and 36–38 weeks. (B) Boxplots showing the relative expression values for *NOTCH2NLA/B/C* in women with history of preeclampsia (*N* = 29) compared to women with no history of preeclampsia (PE) (*N* = 271).

## Discussion

We explored RNA expression using RNAseq in PBMCs from women developing preeclampsia compared to normal pregnant women at gestational weeks 22–24 and 36–38, and at 5-year follow-up. Our main findings: (1) many significantly differentially expressed RNAs in women developing preeclampsia compared to normal pregnancies, mostly at 36–38 weeks; (2) the majority biotype for upregulated RNAs at 36–38 weeks was protein-coding, while most downregulated RNAs were lncRNA; (3) downregulated lncRNAs correlated with markers of endothelial dysfunction, vascular stress, and platelet activation; and (4) overrepresentation analyses and manual exploration of data revealed increased activation of adaptive immune responses, as well as dampening of innate immune response, including the Notch signaling pathway. Taken together, PBMCs from women developing preeclampsia display a prominent regulation of immune-related RNAs when approaching term with enrichment of pathways supporting dampening of innate immune responses but enhanced adaptive immune cell activation. The association between downregulated lncRNAs involved in mitochondrial function and markers of vascular stress and dysfunction may suggest a role for these lncRNA in promoting excessive inflammatory signaling in preeclampsia, although this requires further study.

### A note on participant characteristics and time point variation

Key characteristics of women with preeclampsia such as blood pressure were not significantly different between the two groups for the sequencing cohorts at each time point, including 36–38 weeks when preeclampsia has usually manifested. This could be due to sample matching and low sample size. Indeed, when comparing blood pressures in the total validation cohort (*N* = 215 normal pregnant controls, *N* = 37 preeclampsia), there are significant differences in blood pressures at gestational weeks 36–38, as well as differences in angiogenic factors sFlt-1 and PlGF.

Despite use of different RNA isolation and cleanup protocols at each time point for the RNAseq samples, variance partition analysis (Supplemental Figure S1) showed that time point and RNA isolation methods were not large contributors to variance in the RNAseq data overall. However, due to the additional challenge of small sample sizes, we decided not to use longitudinal analyses for the RNAseq data in this study.

### Dysregulated immune signaling in women developing preeclampsia

Preeclampsia is characterized by a maladaptive maternal immune response [16]. Circulating monocytes from women with preeclampsia release more inflammatory cytokines than in normal pregnancy, suggesting a reduced ability to regulate inflammatory processes [54]. Our data in women with late-onset preeclampsia support this in several ways. First, we found upregulated extracellular danger signals such as *HSPB1* [55] and *PDX2* [56], and the intracellular sensor *KEAP1* which responds to stress signals and triggers immune responses [57, 58]. In addition, several mitochondrial pseudogenes were downregulated, and mitochondrial dysfunction has been shown to trigger and amplify maladaptive immune response in preeclampsia [59, 60]. Women with preeclampsia demonstrate enhanced uptake of EVs [18], which could contain dysfunctional placental mitochondria, potentially activating immune cells [61].

Second, we show downregulation of pathways involved in innate immune activity, including Toll-like receptors (TLRs), which bridge innate and adaptive immune responses through release of proinflammatory cytokines via NF-κB [62]. Furthermore, upregulation of transcripts with inhibitory effects on innate immune pathways such as TLR (*SIGIRR* [63]) and NF-κB signaling (*IRAK3* [64, 65], *LRRC14* [66], *NLRC3* [67, 68], and *NFKBIB* (*IκBβ*) [69]), combined with downregulation of RNAs coding for interferon-induced proteins and viral RNA sensors like *RIG-I* [70], both important in early antiviral defense [71], further indicates impaired innate immune response in these women.

Third, terms involved in translation, as well as RNAs encoding immunoglobulins, ribosomes, and histones, were upregulated at 36–38 weeks, reflecting increased protein synthesis and cell activation, which may occur in response to infection, inflammation, or stress [72–74]. Activation of neutrophils [75], macrophages, and lymphocytes is known in preeclampsia [16], and in this study, various RNAs involved in immune cell activation, recruitment, and migration were upregulated. This included RNAs encoding T-cell receptors and T-cell activation [76–79], as well as RNAs expressed in tissue resident memory CD8^+^ T-cells, *CXCR3, CD27* [80], *RUNX3* [81], *PRDM1* and *ZNF683* (Hobit) [82]. These T-cells may rejoin the circulation [83–85], and we speculate if they rejoined from the decidua [80, 85]. Indeed, our deconvolution analysis showed increased abundance of CD8^+^ T cells during pregnancy in women with preeclampsia. Lastly, increased expression of *LAG3* [86] and *ADGRG1* [87] may reflect exhausted CD8^+^ T cells circulating in these women.

### Altered Notch signaling in PBMCs from women developing preeclampsia

Activation of developmental signaling pathways such as Wnt and Notch have been shown to play a critical role in promoting trophoblast invasion during pregnancy [88, 89], and disruption of these pathways may cause shallow invasion and inadequate remodeling of spiral arteries in preeclampsia [90]. While a concerted downregulation of notch receptors, ligands, and target genes has been shown in placental tissue from late-onset preeclampsia [91], less is known about Notch signaling in immune cells from these women. Notch signaling was one of the most enriched GO pathways in this study despite low gene count, driven by the concerted downregulation of *NOTCH2NL* variants. NOTCH2NL proteins may inhibit NOTCH2-ligand binding, leading to activation of the NOTCH pathway [92, 93], while *NOTCH2NLA* silencing has been shown to inhibit proliferation and migration, and promote apoptosis, in an ovarian cancer cell line [94]. Attenuated Notch signaling was supported by upregulated RNAs *DTX1, DTX3, TLE5*, and *MFNG* in the current study, which are known to inhibit Notch signaling [95–98]. Altered Notch signaling in the circulation of these women may reflect impaired placental development through reduced trophoblast invasion and vascular formation [88, 99].

The discrepancy between RNAseq results and qPCR validation of *NOTCH2NL* variants is possibly owed to qPCR primers picking up all three variants, masking the individual signals of *NOTCH2NLA, NOTCH2NLB*, and *NOTCH2NLC*, and as such reflects only the net regulation of all three variants combined at each time point. The sample sizes for RNAseq were also small and more vulnerable to sample heterogeneity, and validating results from small groups at 36–38 weeks (*N* = 6 controls, *N* = 4 preeclampsia) to a larger cohort (*N* = 215 controls, *N* = 37 preeclampsia) may yield more robust data on differences between groups, and lead to a discrepancy between RNAseq and qPCR results.

Correlation of *NOTCH2NLA/B/C* with clinical markers yielded some significant but weak negative associations with AOPP and beta-galactosidase during pregnancy, markers of oxidative stress and senescence, respectively. While current data is limited, NOTCH inhibition seems to have a dual and context-specific relationship with oxidative stress. Consequently, the downregulation of *NOTCH2NLA/B/C* in women with preeclampsia could indicate an impaired ability to alleviate oxidative stress through this pathway, although this connection remains purely theoretical.

### Long non-coding RNA involved in mitochondrial function, Notch signaling and immunity decreased during pregnancy in women with preeclampsia

The abundance of lncRNA dysregulated during pregnancy in this study is not unprecedented as several lncRNAs have been implicated in the development of preeclampsia; however, mostly assessed in placental tissue [21, 100–102]. Zhu et al. investigated lncRNA expression in whole blood from three healthy pregnancies and three complicated by preeclampsia at early term and found a total of 896 DEGs of which the majority (*n* = 586) were upregulated in women with preeclampsia [103, 104]. In contrast, we observed primarily downregulated lncRNA in PBMCs from women with preeclampsia, possibly partly explained by the abundance of neutrophils in whole blood where counts and activation status are typically higher in preeclampsia [105].

Several downregulated lncRNAs at 36–38 weeks were involved in mitochondrial function, like *ENSG00000289310*, which is sense intronic to *LRRK2*, as well as *LRRK2* itself, and *CYP1B1-*AS1 whose reduction has been linked to mitochondrial dysfunction [106]. Other downregulated lncRNAs indicate dysregulated immune signaling, namely *BACH1-IT2* linked to inflammatory response pathways [107] and *LINC01410* which mediates activation of the Notch signaling pathway [108]. *CHASERR* is involved in T cell regulation, and the knockdown of this lncRNA has been associated with the upregulation of genes involved in T cell activation [109], and could corroborate the overall increased immune cell activation observed in this study.

Most lncRNAs did not correlate with leukocyte activation markers. However, their negative correlation with sFlt-1 and MPO could potentially link downregulated lncRNAs to increased disease severity and vascular stress [110–112], although this association requires further investigation. Additionally, the positive correlation between these lncRNAs and P-selectin, a platelet activation marker which is generally higher in PE [113], may reflect some interaction between leukocytes and platelets during pregnancy. Indeed, platelets contain abundant lncRNA [114] and may transfer RNA to leukocytes [115], although the existence and nature of platelet–leukocyte interactions during pregnancy, with or without preeclampsia, are still unclear.

### Summary

In summary, our results broadly indicate reduced ability to regulate inflammatory processes in women with preeclampsia. This is reflected by the dampening of innate immune mechanisms and altered Notch signaling, indications of mitochondrial dysfunction, and increased activation of adaptive immune cells, possibly mediated by platelet activation. The function of dysregulated lncRNAs in PBMCs and their potential impact on preeclampsia development and cardiovascular health warrant further study.

### Limitations

Limitations of this study include the low number of samples for RNA sequencing, few cases of preeclampsia overall and heterogeneity among preeclampsia cases, one being early-onset and included in the sequencing analysis at 22–24 weeks and at 5-year follow-up. Variation in lab instrumentation at each time point negated use of longitudinal analyses, and primers detected the highly similar *NOTCH2NLA, NOTCH2NLB*, and *NOTCH2NLC* whose relative abundances may differ. Several correlations analyses were performed but not investigated further and do not on their own imply causality. Other limitations include limited participant heterogeneity overall, as women were included from a Norwegian hospital, and lack of functional in vitro experiments.

## Supplementary Material

Supplementary_material_ioag143

## Data Availability

The data sets analyzed during the current study are not publicly available due to patient privacy restrictions but are available from the corresponding author on reasonable request.
