# Supplementary material for: PBMC-derived RNA signatures show impaired ability to regulate immune responses in women developing preeclampsia
Source: Biol Reprod. 2026 Jul 9;115(4):1212–25. doi: 10.1093/biolre/ioag143 (PMC13669408; doi:10.1093/biolre/ioag143)
Supplement: Supplementary file 1 [file supplementary_material_ioag143.zip › Supplemental_Figures_Revised_II_-_Clean_ioag143.docx]

# Supplemental Figures

PBMC-derived RNA signatures show impaired ability to regulate immune responses in women developing preeclampsia

**Figure S1:** Post-mapping quality control plots. A) Cumulative Gene Assignment Diversity plot from QoRTs visualizing library complexity. Plot is from a preeclampsia sample at 22-24 weeks (randomly selected). B) Gene-Body Coverage plot from QoRTs visualizing possible degradation patterns from the same sample as in A)C) Box plots of counts per million (CPM) values from all samples. Values are log transformed. Blue line is the median CPM value. D) Violin plot from variancePartition (see methods) showing the total variance (%) explained by various technical and biological factors in the total RNAseq data set including timepoints 22-24 weeks, 5-year follow-up (N=7 normal pregnant controls and N=9 preeclampsia) and 36-38 weeks (N=6 normal pregnant controls and N=4 preeclampsia).

**Figure S2:** Illustrative views of three-dimensional (3D) principal component (PC) analysis of the top 500 variable genes from RNA sequencing samples colored by status (normal pregnant control or preeclampsia) and timepoint (22-24 weeks, 36-38 weeks or 5-year follow-up). HC = normal pregnant controls, PE = preeclampsia. Illustrated using plotly v4.12.0 in R. The interactive 3D plot can be accessed via Zenodo using doi: 10.5281/zenodo.20830108.
