# Supplementary material for: PBMC-derived RNA signatures show impaired ability to regulate immune responses in women developing preeclampsia
Source: Biol Reprod. 2026 Jul 9;115(4):1212–25. doi: 10.1093/biolre/ioag143 (PMC13669408; doi:10.1093/biolre/ioag143)
Supplement: Supplementary file 1 [file supplementary_material_ioag143.zip › Supplemental_Tables_Revised-Clean_ioag143.docx]

# Supplemental Tables

PBMC-derived RNA signatures show impaired ability to regulate immune responses in women developing preeclampsia

**Table S1:** Significantly upregulated RNAs at gestational weeks 22-24 in women developing preeclampsia (N=9) vs normal pregnancy (N=7). FC = fold change, p-adj = FDR-adjusted p-value

| **Ensembl** | **Symbol** | **Description** | **Biotype** | **log_2_FC** | **p-adj** |
| --- | --- | --- | --- | --- | --- |
| ENSG00000263711 | LINC02864 | long intergenic non-protein coding RNA 2864 | ncRNA | 7.1 | 0.027 |
| ENSG00000117600 | PLPPR4 | phospholipid phosphatase related 4 | protein-coding | 7.0 | 0.019 |
| ENSG00000144285 | SCN1A | sodium voltage-gated channel alpha subunit 1 | protein-coding | 6.6 | 0.049 |
| ENSG00000145794 | MEGF10 | multiple EGF like domains 10 | protein-coding | 5.5 | 0.040 |
| ENSG00000283503 | LINC01902 | long intergenic non-protein coding RNA 1902 | ncRNA | 5.1 | 0.037 |
| ENSG00000165899 | OTOGL | otogelin like | protein-coding | 4.5 | 0.040 |
| ENSG00000151917 | BEND6 | BEN domain containing 6 | protein-coding | 2.7 | 0.040 |
| ENSG00000175985 | PLEKHD1 | pleckstrin homology and coiled-coil domain containing D1 | protein-coding | 2.6 | 0.005 |
| ENSG00000221882 | OR3A2 | olfactory receptor family 3 subfamily A member 2 | protein-coding | 2.3 | 0.019 |
| ENSG00000260314 | MRC1 | mannose receptor C-type 1 | protein-coding | 1.5 | 0.027 |

**FC = fold change, p-adj = FDR-adjusted p-value**

**Table S2:** Significantly upregulated RNAs at gestational weeks 36-38 in women developing preeclampsia (N=4) vs normal pregnancy (N=6).

| **Ensembl** | **Symbol** | **Description** | **Biotype** | **log_2_FC** | **p-adj** |
| --- | --- | --- | --- | --- | --- |
| ENSG00000211895 | IGHA1 | immunoglobulin heavy constant alpha 1 | other | 3.6 | <0.001 |
| ENSG00000287080 | H3C3 | H3 clustered histone 3 | protein-coding | 3.0 | 0.032 |
| ENSG00000239951 | IGKV3-20 | immunoglobulin kappa variable 3-20 | other | 2.6 | 0.026 |
| ENSG00000211893 | IGHG2 | immunoglobulin heavy constant gamma 2 (G2m marker) | other | 2.6 | <0.001 |
| ENSG00000211677 | IGLC2 | immunoglobulin lambda constant 2 | other | 2.5 | 0.012 |
| ENSG00000203616 | RHOT1P2 | ras homolog family member T1 pseudogene 2 | pseudo | 2.4 | 0.002 |
| ENSG00000107281 | NPDC1 | neural proliferation. differentiation and control 1 | protein-coding | 2.1 | 0.045 |
| ENSG00000211679 | IGLC3 | immunoglobulin lambda constant 3 (Kern-Oz+ marker) | other | 2.1 | 0.011 |
| ENSG00000176083 | ZNF683 | zinc finger protein 683 | protein-coding | 2.1 | 0.011 |
| ENSG00000240356 | RPL23AP7 | ribosomal protein L23a pseudogene 7 | pseudo | 2.1 | 0.030 |
| ENSG00000105640 | RPL18A | ribosomal protein L18a | protein-coding | 2.0 | <0.001 |
| ENSG00000106211 | HSPB1 | heat shock protein family B (small) member 1 | protein-coding | 2.0 | 0.020 |
| ENSG00000164897 | TMUB1 | transmembrane and ubiquitin like domain containing 1 | protein-coding | 1.9 | 0.009 |
| ENSG00000176101 | SSNA1 | SS nuclear autoantigen 1 | protein-coding | 1.9 | 0.018 |
| ENSG00000211592 | IGKC | immunoglobulin kappa constant | other | 1.9 | 0.027 |
| ENSG00000290541 | CYP4F29P | cytochrome P450 family 4 subfamily F member 29. pseudogene | pseudo | 1.9 | 0.018 |
| ENSG00000083845 | RPS5 | ribosomal protein S5 | protein-coding | 1.9 | <0.001 |
| ENSG00000173762 | CD7 | CD7 molecule | protein-coding | 1.9 | 0.018 |
| ENSG00000054148 | PHPT1 | phosphohistidine phosphatase 1 | protein-coding | 1.8 | 0.015 |
| ENSG00000137818 | RPLP1 | ribosomal protein lateral stalk subunit P1 | protein-coding | 1.8 | 0.003 |
| ENSG00000212135 | SNORD67 | small nucleolar RNA. C/D box 67 | snoRNA | 1.8 | 0.039 |
| ENSG00000140988 | RPS2 | ribosomal protein S2 | protein-coding | 1.8 | <0.001 |
| ENSG00000277157 | H4C4 | H4 clustered histone 4 | protein-coding | 1.8 | 0.001 |
| ENSG00000089692 | LAG3 | lymphocyte activating 3 | protein-coding | 1.8 | 0.013 |
| ENSG00000139193 | CD27 | CD27 molecule | protein-coding | 1.8 | <0.001 |
| ENSG00000256937 | KRT17P8 | keratin 17 pseudogene 8 | pseudo | 1.8 | 0.047 |
| ENSG00000188322 | SBK1 | SH3 domain binding kinase 1 | protein-coding | 1.8 | 0.001 |
| ENSG00000167658 | EEF2 | eukaryotic translation elongation factor 2 | protein-coding | 1.8 | <0.001 |
| ENSG00000067840 | PDZD4 | PDZ domain containing 4 | protein-coding | 1.8 | 0.023 |
| ENSG00000167100 | SAMD14 | sterile alpha motif domain containing 14 | protein-coding | 1.8 | 0.033 |
| ENSG00000177697 | CD151 | CD151 molecule (Raph blood group) | protein-coding | 1.8 | 0.007 |
| ENSG00000130255 | RPL36 | ribosomal protein L36 | protein-coding | 1.8 | 0.001 |
| ENSG00000166289 | PLEKHF1 | pleckstrin homology and FYVE domain containing 1 | protein-coding | 1.7 | 0.007 |
| ENSG00000278705 | H4C2 | H4 clustered histone 2 | protein-coding | 1.7 | 0.001 |
| ENSG00000101335 | MYL9 | myosin light chain 9 | protein-coding | 1.7 | 0.045 |
| ENSG00000062524 | LTK | leukocyte receptor tyrosine kinase | protein-coding | 1.7 | 0.005 |
| ENSG00000167094 | TTC16 | tetratricopeptide repeat domain 16 | protein-coding | 1.7 | <0.001 |
| ENSG00000197457 | STMN3 | stathmin 3 | protein-coding | 1.7 | 0.005 |
| ENSG00000206341 | HLA-H | major histocompatibility complex. class I. H | pseudo | 1.7 | 0.025 |
| ENSG00000105701 | FKBP8 | FKBP prolyl isomerase 8 | protein-coding | 1.7 | 0.026 |
| ENSG00000133243 | BTBD2 | BTB domain containing 2 | protein-coding | 1.7 | 0.017 |
| ENSG00000211897 | IGHG3 | immunoglobulin heavy constant gamma 3 (G3m marker) | other | 1.7 | 0.030 |
| ENSG00000104964 | TLE5 | TLE family member 5. transcriptional modulator | protein-coding | 1.7 | <0.001 |
| ENSG00000101057 | MYBL2 | MYB proto-oncogene like 2 | protein-coding | 1.7 | 0.026 |
| ENSG00000135916 | ITM2C | integral membrane protein 2C | protein-coding | 1.7 | 0.008 |
| ENSG00000174171 | LOC105370792 | uncharacterized LOC105370792 | ncRNA | 1.7 | 0.002 |
| ENSG00000186174 | BCL9L | BCL9 like | protein-coding | 1.7 | 0.002 |
| ENSG00000167797 | CDK2AP2 | cyclin dependent kinase 2 associated protein 2 | protein-coding | 1.7 | 0.004 |
| ENSG00000182871 | COL18A1 | collagen type XVIII alpha 1 chain | protein-coding | 1.6 | 0.034 |
| ENSG00000167526 | RPL13 | ribosomal protein L13 | protein-coding | 1.6 | <0.001 |
| ENSG00000185187 | SIGIRR | single Ig and TIR domain containing | protein-coding | 1.6 | 0.020 |
| ENSG00000142327 | RNPEPL1 | arginyl aminopeptidase like 1 | protein-coding | 1.6 | 0.035 |
| ENSG00000140854 | KATNB1 | katanin regulatory subunit B1 | protein-coding | 1.6 | 0.035 |
| ENSG00000196924 | FLNA | filamin A | protein-coding | 1.6 | 0.016 |
| ENSG00000201098 | RNY1 | RNA. Ro60-associated Y1 | ncRNA | 1.6 | 0.048 |
| ENSG00000198520 | ARMH1 | armadillo like helical domain containing 1 | protein-coding | 1.6 | 0.007 |
| ENSG00000107331 | ABCA2 | ATP binding cassette subfamily A member 2 | protein-coding | 1.6 | 0.032 |
| ENSG00000105699 | LSR | lipolysis stimulated lipoprotein receptor | protein-coding | 1.6 | 0.006 |
| ENSG00000161016 | RPL8 | ribosomal protein L8 | protein-coding | 1.6 | <0.001 |
| ENSG00000184009 | ACTG1 | actin gamma 1 | protein-coding | 1.6 | <0.001 |
| ENSG00000154146 | NRGN | neurogranin | protein-coding | 1.6 | 0.034 |
| ENSG00000185340 | GAS2L1 | growth arrest specific 2 like 1 | protein-coding | 1.6 | 0.029 |
| ENSG00000179526 | SHARPIN | SHANK associated RH domain interactor | protein-coding | 1.6 | 0.029 |
| ENSG00000215788 | TNFRSF25 | TNF receptor superfamily member 25 | protein-coding | 1.6 | <0.001 |
| ENSG00000126461 | SCAF1 | SR-related CTD associated factor 1 | protein-coding | 1.6 | 0.021 |
| ENSG00000077984 | CST7 | cystatin F | protein-coding | 1.6 | 0.001 |
| ENSG00000220517 | ASS1P1 | argininosuccinate synthetase 1 pseudogene 1 | pseudo | 1.6 | 0.009 |
| ENSG00000167815 | PRDX2 | peroxiredoxin 2 | protein-coding | 1.6 | <0.001 |
| ENSG00000135144 | DTX1 | deltex E3 ubiquitin ligase 1 | protein-coding | 1.6 | 0.005 |
| ENSG00000105374 | NKG7 | natural killer cell granule protein 7 | protein-coding | 1.6 | 0.001 |
| ENSG00000129968 | ABHD17A | abhydrolase domain containing 17A. depalmitoylase | protein-coding | 1.6 | 0.026 |
| ENSG00000074370 | ATP2A3 | ATPase sarcoplasmic/endoplasmic reticulum Ca2+ transporting 3 | protein-coding | 1.6 | 0.006 |
| ENSG00000070423 | RNF126 | ring finger protein 126 | protein-coding | 1.6 | 0.038 |
| ENSG00000211898 | IGHD | immunoglobulin heavy constant delta | other | 1.6 | 0.044 |
| ENSG00000120885 | CLU | clusterin | protein-coding | 1.6 | 0.029 |
| ENSG00000172543 | CTSW | cathepsin W | protein-coding | 1.6 | 0.001 |
| ENSG00000174996 | KLC2 | kinesin light chain 2 | protein-coding | 1.6 | 0.010 |
| ENSG00000116649 | SRM | spermidine synthase | protein-coding | 1.6 | 0.013 |
| ENSG00000238835 | SCARNA18 | small Cajal body-specific RNA 18 | ncRNA | 1.6 | 0.036 |
| ENSG00000185262 | UBALD2 | UBA like domain containing 2 | protein-coding | 1.6 | 0.009 |
| ENSG00000071564 | TCF3 | transcription factor 3 | protein-coding | 1.6 | 0.010 |
| ENSG00000171159 | BBLN | bublin coiled coil protein | protein-coding | 1.6 | 0.027 |
| ENSG00000276045 | ORAI1 | ORAI calcium release-activated calcium modulator 1 | protein-coding | 1.5 | 0.001 |
| ENSG00000180035 | ZNF48 | zinc finger protein 48 | protein-coding | 1.5 | 0.001 |
| ENSG00000141522 | ARHGDIA | Rho GDP dissociation inhibitor alpha | protein-coding | 1.5 | 0.047 |
| ENSG00000186049 | KRT73 | keratin 73 | protein-coding | 1.5 | 0.001 |
| ENSG00000196700 | ZNF512B | zinc finger protein 512B | protein-coding | 1.5 | 0.028 |
| ENSG00000170486 | KRT72 | keratin 72 | protein-coding | 1.5 | 0.017 |
| ENSG00000136717 | BIN1 | bridging integrator 1 | protein-coding | 1.5 | 0.027 |
| ENSG00000167106 | EEIG1 | estrogen-induced osteoclastogenesis regulator 1 | protein-coding | 1.5 | 0.001 |
| ENSG00000008517 | IL32 | interleukin 32 | protein-coding | 1.5 | 0.001 |
| ENSG00000158050 | DUSP2 | dual specificity phosphatase 2 | protein-coding | 1.5 | 0.012 |
| ENSG00000104823 | ECH1 | enoyl-CoA hydratase 1 | protein-coding | 1.5 | 0.002 |
| ENSG00000115085 | ZAP70 | zeta chain of T cell receptor associated protein kinase 70 | protein-coding | 1.5 | 0.011 |
| ENSG00000126464 | PRR12 | proline rich 12 | protein-coding | 1.5 | 0.036 |
| ENSG00000287823 |  | novel transcript | ncRNA | 1.5 | 0.007 |
| ENSG00000100299 | ARSA | arylsulfatase A | protein-coding | 1.5 | 0.027 |
| ENSG00000167114 | SLC27A4 | solute carrier family 27 member 4 | protein-coding | 1.5 | 0.012 |
| ENSG00000122140 | MRPS2 | mitochondrial ribosomal protein S2 | protein-coding | 1.5 | 0.015 |
| ENSG00000129925 | PGAP6 | post-GPI attachment to proteins 6 | protein-coding | 1.5 | 0.045 |
| ENSG00000254995 | STX16-NPEPL1 | STX16-NPEPL1 readthrough (NMD candidate) | ncRNA | 1.5 | 0.010 |
| ENSG00000187837 | H1-2 | H1.2 linker histone. cluster member | protein-coding | 1.5 | 0.003 |
| ENSG00000176340 | COX8A | cytochrome c oxidase subunit 8A | protein-coding | 1.5 | 0.001 |
| ENSG00000125534 | PPDPF | pancreatic progenitor cell differentiation and proliferation factor | protein-coding | 1.5 | 0.022 |
| ENSG00000100316 | RPL3 | ribosomal protein L3 | protein-coding | 1.5 | <0.001 |
| ENSG00000235683 |  | leucine zipper-EF-hand containing transmembrane protein 1 (LETM1) pseudogene | pseudo | 1.5 | 0.022 |
| ENSG00000185101 | ANO9 | anoctamin 9 | protein-coding | 1.5 | 0.019 |
| ENSG00000139725 | RHOF | ras homolog family member F. filopodia associated | protein-coding | 1.5 | <0.001 |
| ENSG00000184260 | H2AC20 | H2A clustered histone 20 | protein-coding | 1.5 | 0.002 |
| ENSG00000131634 | TMEM204 | transmembrane protein 204 | protein-coding | 1.5 | 0.002 |
| ENSG00000176108 | CHMP6 | charged multivesicular body protein 6 | protein-coding | 1.5 | 0.030 |
| ENSG00000203485 | INF2 | inverted formin 2 | protein-coding | 1.5 | 0.019 |
| ENSG00000102977 | ACD | ACD shelterin complex subunit and telomerase recruitment factor | protein-coding | 1.5 | 0.023 |
| ENSG00000115268 | RPS15 | ribosomal protein S15 | protein-coding | 1.5 | <0.001 |
| ENSG00000159753 | CARMIL2 | capping protein regulator and myosin 1 linker 2 | protein-coding | 1.5 | 0.028 |
| ENSG00000165272 | AQP3 | aquaporin 3 (Gill blood group) | protein-coding | 1.5 | <0.001 |
| ENSG00000157303 | SUSD3 | sushi domain containing 3 | protein-coding | 1.5 | 0.040 |
| ENSG00000198286 | CARD11 | caspase recruitment domain family member 11 | protein-coding | 1.5 | 0.005 |
| ENSG00000137309 | HMGA1 | high mobility group AT-hook 1 | protein-coding | 1.5 | 0.002 |
| ENSG00000130590 | SAMD10 | sterile alpha motif domain containing 10 | protein-coding | 1.5 | 0.004 |
| ENSG00000100450 | GZMH | granzyme H | protein-coding | 1.5 | 0.015 |
| ENSG00000184887 | BTBD6 | BTB domain containing 6 | protein-coding | 1.5 | 0.003 |
| ENSG00000142541 | RPL13A | ribosomal protein L13a | protein-coding | 1.5 | <0.001 |
| ENSG00000197562 | RAB40C | RAB40C. member RAS oncogene family | protein-coding | 1.5 | 0.034 |
| ENSG00000177600 | RPLP2 | ribosomal protein lateral stalk subunit P2 | protein-coding | 1.4 | 0.005 |
| ENSG00000181035 | SLC25A42 | solute carrier family 25 member 42 | protein-coding | 1.4 | 0.002 |
| ENSG00000140682 | TGFB1I1 | transforming growth factor beta 1 induced transcript 1 | protein-coding | 1.4 | 0.041 |
| ENSG00000196787 | H2AC11 | H2A clustered histone 11 | protein-coding | 1.4 | 0.015 |
| ENSG00000171823 | FBXL14 | F-box and leucine rich repeat protein 14 | protein-coding | 1.4 | 0.023 |
| ENSG00000171858 | RPS21 | ribosomal protein S21 | protein-coding | 1.4 | <0.001 |
| ENSG00000125520 | SLC2A4RG | SLC2A4 regulator | protein-coding | 1.4 | 0.002 |
| ENSG00000250731 | TPM3P6 | tropomyosin 3 pseudogene 6 | pseudo | 1.4 | 0.035 |
| ENSG00000110448 | CD5 | CD5 molecule | protein-coding | 1.4 | 0.004 |
| ENSG00000105063 | PPP6R1 | protein phosphatase 6 regulatory subunit 1 | protein-coding | 1.4 | 0.044 |
| ENSG00000130827 | PLXNA3 | plexin A3 | protein-coding | 1.4 | 0.012 |
| ENSG00000110651 | CD81 | CD81 molecule | protein-coding | 1.4 | 0.022 |
| ENSG00000154016 | GRAP | GRB2 related adaptor protein | protein-coding | 1.4 | 0.028 |
| ENSG00000186716 | BCR | BCR activator of RhoGEF and GTPase | protein-coding | 1.4 | 0.006 |
| ENSG00000140691 | ARMC5 | armadillo repeat containing 5 | protein-coding | 1.4 | 0.048 |
| ENSG00000132382 | MYBBP1A | MYB binding protein 1a | protein-coding | 1.4 | 0.038 |
| ENSG00000160877 | NACC1 | nucleus accumbens associated 1 | protein-coding | 1.4 | 0.021 |
| ENSG00000125817 | CENPB | centromere protein B | protein-coding | 1.4 | 0.004 |
| ENSG00000131653 | TRAF7 | TNF receptor associated factor 7 | protein-coding | 1.4 | 0.037 |
| ENSG00000096433 | ITPR3 | inositol 1.4.5-trisphosphate receptor type 3 | protein-coding | 1.4 | 0.034 |
| ENSG00000144476 | ACKR3 | atypical chemokine receptor 3 | protein-coding | 1.4 | 0.001 |
| ENSG00000108298 | RPL19 | ribosomal protein L19 | protein-coding | 1.4 | <0.001 |
| ENSG00000197858 | GPAA1 | glycosylphosphatidylinositol anchor attachment 1 | protein-coding | 1.4 | 0.040 |
| ENSG00000148773 | MKI67 | marker of proliferation Ki-67 | protein-coding | 1.4 | 0.033 |
| ENSG00000214706 | IFRD2 | interferon related developmental regulator 2 | protein-coding | 1.4 | 0.032 |
| ENSG00000142459 | EVI5L | ecotropic viral integration site 5 like | protein-coding | 1.4 | 0.030 |
| ENSG00000161999 | JMJD8 | jumonji domain containing 8 | protein-coding | 1.4 | 0.029 |
| ENSG00000182866 | LCK | LCK proto-oncogene. Src family tyrosine kinase | protein-coding | 1.4 | 0.001 |
| ENSG00000102178 | UBL4A | ubiquitin like 4A | protein-coding | 1.4 | 0.006 |
| ENSG00000153563 | CD8A | CD8a molecule | protein-coding | 1.4 | 0.019 |
| ENSG00000132819 | RBM38 | RNA binding motif protein 38 | protein-coding | 1.4 | 0.006 |
| ENSG00000064666 | CNN2 | calponin 2 | protein-coding | 1.4 | 0.002 |
| ENSG00000079435 | LIPE | lipase E. hormone sensitive type | protein-coding | 1.4 | 0.019 |
| ENSG00000213626 | LBH | LBH regulator of WNT signaling pathway | protein-coding | 1.4 | 0.001 |
| ENSG00000027869 | SH2D2A | SH2 domain containing 2A | protein-coding | 1.4 | 0.006 |
| ENSG00000227191 | TRGC2 | T cell receptor gamma constant 2 | other | 1.4 | 0.008 |
| ENSG00000205336 | ADGRG1 | adhesion G protein-coupled receptor G1 | protein-coding | 1.4 | 0.021 |
| ENSG00000110047 | EHD1 | EH domain containing 1 | protein-coding | 1.4 | 0.004 |
| ENSG00000114767 | RRP9 | ribosomal RNA processing 9. U3 small nucleolar RNA binding protein | protein-coding | 1.4 | 0.015 |
| ENSG00000136878 | USP20 | ubiquitin specific peptidase 20 | protein-coding | 1.4 | 0.018 |
| ENSG00000186577 | SMIM29 | small integral membrane protein 29 | protein-coding | 1.4 | 0.026 |
| ENSG00000167895 | TMC8 | transmembrane channel like 8 | protein-coding | 1.4 | 0.014 |
| ENSG00000119714 | GPR68 | G protein-coupled receptor 68 | protein-coding | 1.4 | 0.002 |
| ENSG00000186810 | CXCR3 | C-X-C motif chemokine receptor 3 | protein-coding | 1.4 | 0.046 |
| ENSG00000211751 | TRBC1 | T cell receptor beta constant 1 | other | 1.4 | <0.001 |
| ENSG00000226210 | WASH8P | WAS protein family homolog 8. pseudogene | pseudo | 1.4 | 0.032 |
| ENSG00000169976 | SF3B5 | splicing factor 3b subunit 5 | protein-coding | 1.4 | 0.009 |
| ENSG00000197903 | H2BC12 | H2B clustered histone 12 | protein-coding | 1.4 | 0.039 |
| ENSG00000180879 | SSR4 | signal sequence receptor subunit 4 | protein-coding | 1.4 | 0.009 |
| ENSG00000126903 | SLC10A3 | solute carrier family 10 member 3 | protein-coding | 1.4 | 0.023 |
| ENSG00000113758 | DBN1 | drebrin 1 | protein-coding | 1.4 | 0.017 |
| ENSG00000090097 | PCBP4 | poly(rC) binding protein 4 | protein-coding | 1.4 | 0.009 |
| ENSG00000101224 | CDC25B | cell division cycle 25B | protein-coding | 1.4 | <0.001 |
| ENSG00000149541 | B3GAT3 | beta-1.3-glucuronyltransferase 3 | protein-coding | 1.4 | 0.010 |
| ENSG00000106683 | LIMK1 | LIM domain kinase 1 | protein-coding | 1.4 | 0.031 |
| ENSG00000167702 | KIFC2 | kinesin family member C2 | protein-coding | 1.4 | 0.028 |
| ENSG00000105372 | RPS19 | ribosomal protein S19 | protein-coding | 1.4 | 0.001 |
| ENSG00000105122 | RASAL3 | RAS protein activator like 3 | protein-coding | 1.4 | 0.033 |
| ENSG00000110711 | AIP | aryl hydrocarbon receptor interacting protein | protein-coding | 1.4 | 0.006 |
| ENSG00000184381 | PLA2G6 | phospholipase A2 group VI | protein-coding | 1.3 | 0.015 |
| ENSG00000112306 | RPS12 | ribosomal protein S12 | protein-coding | 1.3 | <0.001 |
| ENSG00000172508 | CARNS1 | carnosine synthase 1 | protein-coding | 1.3 | 0.004 |
| ENSG00000014216 | CAPN1 | calpain 1 | protein-coding | 1.3 | 0.010 |
| ENSG00000100304 | TTLL12 | tubulin tyrosine ligase like 12 | protein-coding | 1.3 | 0.027 |
| ENSG00000099875 | MKNK2 | MAPK interacting serine/threonine kinase 2 | protein-coding | 1.3 | 0.020 |
| ENSG00000087074 | PPP1R15A | protein phosphatase 1 regulatory subunit 15A | protein-coding | 1.3 | 0.014 |
| ENSG00000198055 | GRK6 | G protein-coupled receptor kinase 6 | protein-coding | 1.3 | 0.037 |
| ENSG00000198792 | TMEM184B | transmembrane protein 184B | protein-coding | 1.3 | 0.025 |
| ENSG00000037280 | FLT4 | fms related receptor tyrosine kinase 4 | protein-coding | 1.3 | 0.013 |
| ENSG00000111678 | C12orf57 | chromosome 12 open reading frame 57 | protein-coding | 1.3 | <0.001 |
| ENSG00000172270 | BSG | basigin (Ok blood group) | protein-coding | 1.3 | 0.044 |
| ENSG00000158856 | DMTN | dematin actin binding protein | protein-coding | 1.3 | 0.044 |
| ENSG00000157778 | PSMG3 | proteasome assembly chaperone 3 | protein-coding | 1.3 | 0.010 |
| ENSG00000171130 | ATP6V0E2 | ATPase H+ transporting V0 subunit e2 | protein-coding | 1.3 | <0.001 |
| ENSG00000198276 | UCKL1 | uridine-cytidine kinase 1 like 1 | protein-coding | 1.3 | 0.037 |
| ENSG00000136942 | RPL35 | ribosomal protein L35 | protein-coding | 1.3 | 0.003 |
| ENSG00000103145 | HCFC1R1 | host cell factor C1 regulator 1 | protein-coding | 1.3 | 0.044 |
| ENSG00000164896 | FASTK | Fas activated serine/threonine kinase | protein-coding | 1.3 | 0.031 |
| ENSG00000130811 | EIF3G | eukaryotic translation initiation factor 3 subunit G | protein-coding | 1.3 | 0.005 |
| ENSG00000130726 | TRIM28 | tripartite motif containing 28 | protein-coding | 1.3 | 0.017 |
| ENSG00000290021 |  | novel transcript. antisense to BCL6-AS1 | ncRNA | 1.3 | 0.039 |
| ENSG00000198026 | ZNF335 | zinc finger protein 335 | protein-coding | 1.3 | 0.042 |
| ENSG00000103266 | STUB1 | STIP1 homology and U-box containing protein 1 | protein-coding | 1.3 | 0.031 |
| ENSG00000213672 | NCKIPSD | NCK interacting protein with SH3 domain | protein-coding | 1.3 | 0.016 |
| ENSG00000100242 | SUN2 | Sad1 and UNC84 domain containing 2 | protein-coding | 1.3 | 0.022 |
| ENSG00000133619 | KRBA1 | KRAB-A domain containing 1 | protein-coding | 1.3 | 0.047 |
| ENSG00000160959 | LRRC14 | leucine rich repeat containing 14 | protein-coding | 1.3 | 0.013 |
| ENSG00000111788 | DDX12B | DEAD/H-box helicase 12B (pseudogene) | pseudo | 1.3 | 0.048 |
| ENSG00000167543 | TP53I13 | tumor protein p53 inducible protein 13 | protein-coding | 1.3 | 0.027 |
| ENSG00000135127 | BICDL1 | BICD family like cargo adaptor 1 | protein-coding | 1.3 | 0.012 |
| ENSG00000105373 | NOP53 | NOP53 ribosome biogenesis factor | protein-coding | 1.3 | 0.009 |
| ENSG00000279672 |  |  |  | 1.3 | 0.015 |
| ENSG00000095321 | CRAT | carnitine O-acetyltransferase | protein-coding | 1.3 | 0.003 |
| ENSG00000123159 | GIPC1 | GIPC PDZ domain containing family member 1 | protein-coding | 1.3 | 0.016 |
| ENSG00000232487 | RASA3-IT1 | RASA3 intronic transcript 1 | ncRNA | 1.3 | 0.021 |
| ENSG00000109062 | SLC9A3R1 | SLC9A3 regulator 1 | protein-coding | 1.3 | 0.005 |
| ENSG00000179115 | FARSA | phenylalanyl-tRNA synthetase subunit alpha | protein-coding | 1.3 | 0.033 |
| ENSG00000136490 | LIMD2 | LIM domain containing 2 | protein-coding | 1.3 | 0.032 |
| ENSG00000011132 | APBA3 | amyloid beta precursor protein binding family A member 3 | protein-coding | 1.3 | 0.047 |
| ENSG00000100385 | IL2RB | interleukin 2 receptor subunit beta | protein-coding | 1.3 | 0.012 |
| ENSG00000198885 | ITPRIPL1 | ITPRIP like 1 | protein-coding | 1.3 | 0.015 |
| ENSG00000107742 | SPOCK2 | SPARC (osteonectin). cwcv and kazal like domains proteoglycan 2 | protein-coding | 1.3 | 0.001 |
| ENSG00000174903 | RAB1B | RAB1B. member RAS oncogene family | protein-coding | 1.3 | 0.005 |
| ENSG00000074416 | MGLL | monoglyceride lipase | protein-coding | 1.3 | 0.033 |
| ENSG00000105519 | CAPS | calcyphosine | protein-coding | 1.3 | 0.033 |
| ENSG00000166341 | DCHS1 | dachsous cadherin-related 1 | protein-coding | 1.3 | 0.034 |
| ENSG00000064545 | TMEM161A | transmembrane protein 161A | protein-coding | 1.3 | 0.010 |
| ENSG00000238164 | TNFRSF14-AS1 | TNFRSF14 antisense RNA 1 | ncRNA | 1.3 | 0.046 |
| ENSG00000174669 | SLC29A2 | solute carrier family 29 member 2 | protein-coding | 1.3 | 0.047 |
| ENSG00000140320 | BAHD1 | bromo adjacent homology domain containing 1 | protein-coding | 1.3 | 0.006 |
| ENSG00000099381 | SETD1A | SET domain containing 1A. histone lysine methyltransferase | protein-coding | 1.3 | 0.010 |
| ENSG00000130382 | MLLT1 | MLLT1 super elongation complex subunit | protein-coding | 1.3 | 0.047 |
| ENSG00000185189 | NRBP2 | nuclear receptor binding protein 2 | protein-coding | 1.3 | 0.018 |
| ENSG00000101298 | SNPH | syntaphilin | protein-coding | 1.3 | 0.043 |
| ENSG00000170604 | IRF2BP1 | interferon regulatory factor 2 binding protein 1 | protein-coding | 1.3 | 0.036 |
| ENSG00000126391 | FRMD8 | FERM domain containing 8 | protein-coding | 1.2 | 0.042 |
| ENSG00000143851 | PTPN7 | protein tyrosine phosphatase non-receptor type 7 | protein-coding | 1.2 | 0.002 |
| ENSG00000164867 | NOS3 | nitric oxide synthase 3 | protein-coding | 1.2 | 0.017 |
| ENSG00000090006 | LTBP4 | latent transforming growth factor beta binding protein 4 | protein-coding | 1.2 | 0.016 |
| ENSG00000136802 | LRRC8A | leucine rich repeat containing 8 VRAC subunit A | protein-coding | 1.2 | 0.001 |
| ENSG00000237181 | PRKAR1B-AS1 | PRKAR1B antisense RNA 1 | ncRNA | 1.2 | 0.007 |
| ENSG00000277476 |  | novel transcript | ncRNA | 1.2 | 0.026 |
| ENSG00000111640 | GAPDH | glyceraldehyde-3-phosphate dehydrogenase | protein-coding | 1.2 | 0.001 |
| ENSG00000235316 | DUSP8P5 | dual specificity phosphatase 8 pseudogene 5 | pseudo | 1.2 | 0.019 |
| ENSG00000142546 | NOSIP | nitric oxide synthase interacting protein | protein-coding | 1.2 | <0.001 |
| ENSG00000015532 | XYLT2 | xylosyltransferase 2 | protein-coding | 1.2 | 0.028 |
| ENSG00000172534 | HCFC1 | host cell factor C1 | protein-coding | 1.2 | 0.032 |
| ENSG00000124181 | PLCG1 | phospholipase C gamma 1 | protein-coding | 1.2 | 0.007 |
| ENSG00000126705 | AHDC1 | AT-hook DNA binding motif containing 1 | protein-coding | 1.2 | 0.039 |
| ENSG00000180096 | SEPTIN1 | septin 1 | protein-coding | 1.2 | <0.001 |
| ENSG00000167972 | ABCA3 | ATP binding cassette subfamily A member 3 | protein-coding | 1.2 | 0.025 |
| ENSG00000116852 | KIF21B | kinesin family member 21B | protein-coding | 1.2 | 0.010 |
| ENSG00000179262 | RAD23A | RAD23 homolog A. nucleotide excision repair protein | protein-coding | 1.2 | 0.020 |
| ENSG00000072818 | ACAP1 | ArfGAP with coiled-coil. ankyrin repeat and PH domains 1 | protein-coding | 1.2 | 0.007 |
| ENSG00000159618 | ADGRG5 | adhesion G protein-coupled receptor G5 | protein-coding | 1.2 | 0.007 |
| ENSG00000159840 | ZYX | zyxin | protein-coding | 1.2 | 0.024 |
| ENSG00000115073 | ACTR1B | actin related protein 1B | protein-coding | 1.2 | 0.006 |
| ENSG00000274618 | H4C6 | H4 clustered histone 6 | protein-coding | 1.2 | 0.024 |
| ENSG00000182208 | MOB2 | MOB kinase activator 2 | protein-coding | 1.2 | 0.034 |
| ENSG00000105193 | RPS16 | ribosomal protein S16 | protein-coding | 1.2 | <0.001 |
| ENSG00000158863 | FHIP2B | FHF complex subunit HOOK interacting protein 2B | protein-coding | 1.2 | 0.043 |
| ENSG00000289398 |  | novel transcript | ncRNA | 1.2 | 0.036 |
| ENSG00000211772 | TRBC2 | T cell receptor beta constant 2 | other | 1.2 | 0.017 |
| ENSG00000261221 | ZNF865 | zinc finger protein 865 | protein-coding | 1.2 | 0.050 |
| ENSG00000212443 | SNORA53 | small nucleolar RNA. H/ACA box 53 | snoRNA | 1.2 | 0.037 |
| ENSG00000167775 | CD320 | CD320 molecule | protein-coding | 1.2 | 0.029 |
| ENSG00000104856 | RELB | RELB proto-oncogene. NF-kB subunit | protein-coding | 1.2 | 0.048 |
| ENSG00000140688 | RUSF1 | RUS family member 1 | protein-coding | 1.2 | 0.034 |
| ENSG00000227039 | ITGB2-AS1 | ITGB2 antisense RNA 1 | ncRNA | 1.2 | 0.025 |
| ENSG00000167770 | OTUB1 | OTU deubiquitinase. ubiquitin aldehyde binding 1 | protein-coding | 1.2 | 0.016 |
| ENSG00000229474 | PATL2 | PAT1 homolog 2 | protein-coding | 1.2 | 0.009 |
| ENSG00000156858 | PRR14 | proline rich 14 | protein-coding | 1.2 | 0.037 |
| ENSG00000168758 | SEMA4C | semaphorin 4C | protein-coding | 1.2 | 0.016 |
| ENSG00000130520 | LSM4 | LSM4 homolog. U6 small nuclear RNA and mRNA degradation associated | protein-coding | 1.2 | 0.001 |
| ENSG00000204628 | RACK1 | receptor for activated C kinase 1 | protein-coding | 1.2 | <0.001 |
| ENSG00000108107 | RPL28 | ribosomal protein L28 | protein-coding | 1.2 | 0.005 |
| ENSG00000100060 | MFNG | MFNG O-fucosylpeptide 3-beta-N-acetylglucosaminyltransferase | protein-coding | 1.2 | 0.012 |
| ENSG00000198356 | GET3 | guided entry of tail-anchored proteins factor 3. ATPase | protein-coding | 1.2 | 0.019 |
| ENSG00000231500 | RPS18 | ribosomal protein S18 | protein-coding | 1.2 | 0.004 |
| ENSG00000188636 | RTL6 | retrotransposon Gag like 6 | protein-coding | 1.2 | 0.001 |
| ENSG00000007264 | MATK | megakaryocyte-associated tyrosine kinase | protein-coding | 1.2 | 0.050 |
| ENSG00000151929 | BAG3 | BAG cochaperone 3 | protein-coding | 1.2 | <0.001 |
| ENSG00000136807 | CDK9 | cyclin dependent kinase 9 | protein-coding | 1.2 | 0.016 |
| ENSG00000196453 | ZNF777 | zinc finger protein 777 | protein-coding | 1.2 | 0.004 |
| ENSG00000101997 | CCDC22 | coiled-coil domain containing 22 | protein-coding | 1.2 | 0.026 |
| ENSG00000105321 | CCDC9 | coiled-coil domain containing 9 | protein-coding | 1.2 | 0.031 |
| ENSG00000183207 | RUVBL2 | RuvB like AAA ATPase 2 | protein-coding | 1.2 | 0.045 |
| ENSG00000119242 | CCDC92 | coiled-coil domain containing 92 | protein-coding | 1.2 | 0.007 |
| ENSG00000090975 | PITPNM2 | phosphatidylinositol transfer protein membrane associated 2 | protein-coding | 1.2 | 0.015 |
| ENSG00000099991 | CABIN1 | calcineurin binding protein 1 | protein-coding | 1.2 | 0.025 |
| ENSG00000068831 | RASGRP2 | RAS guanyl releasing protein 2 | protein-coding | 1.2 | 0.050 |
| ENSG00000274070 | CASTOR2 | cytosolic arginine sensor for mTORC1 subunit 2 | protein-coding | 1.2 | 0.004 |
| ENSG00000130175 | PRKCSH | protein kinase C substrate 80K-H | protein-coding | 1.2 | 0.012 |
| ENSG00000061273 | HDAC7 | histone deacetylase 7 | protein-coding | 1.2 | 0.006 |
| ENSG00000126457 | PRMT1 | protein arginine methyltransferase 1 | protein-coding | 1.2 | 0.005 |
| ENSG00000177731 | FLII | FLII actin remodeling protein | protein-coding | 1.2 | 0.045 |
| ENSG00000148334 | PTGES2 | prostaglandin E synthase 2 | protein-coding | 1.2 | 0.030 |
| ENSG00000100345 | MYH9 | myosin heavy chain 9 | protein-coding | 1.2 | 0.011 |
| ENSG00000114737 | CISH | cytokine inducible SH2 containing protein | protein-coding | 1.2 | 0.002 |
| ENSG00000149273 | RPS3 | ribosomal protein S3 | protein-coding | 1.2 | <0.001 |
| ENSG00000161381 | PLXDC1 | plexin domain containing 1 | protein-coding | 1.2 | 0.007 |
| ENSG00000163346 | PBXIP1 | PBX homeobox interacting protein 1 | protein-coding | 1.2 | 0.008 |
| ENSG00000206634 | SNORA22 | small nucleolar RNA. H/ACA box 22 | snoRNA | 1.2 | 0.011 |
| ENSG00000141524 | TMC6 | transmembrane channel like 6 | protein-coding | 1.2 | 0.024 |
| ENSG00000187688 | TRPV2 | transient receptor potential cation channel subfamily V member 2 | protein-coding | 1.2 | 0.015 |
| ENSG00000056558 | TRAF1 | TNF receptor associated factor 1 | protein-coding | 1.2 | 0.019 |
| ENSG00000020633 | RUNX3 | RUNX family transcription factor 3 | protein-coding | 1.2 | 0.001 |
| ENSG00000181045 | SLC26A11 | solute carrier family 26 member 11 | protein-coding | 1.2 | 0.006 |
| ENSG00000100075 | SLC25A1 | solute carrier family 25 member 1 | protein-coding | 1.2 | 0.025 |
| ENSG00000180739 | S1PR5 | sphingosine-1-phosphate receptor 5 | protein-coding | 1.2 | 0.009 |
| ENSG00000178498 | DTX3 | deltex E3 ubiquitin ligase 3 | protein-coding | 1.2 | 0.015 |
| ENSG00000275395 | FCGBP | Fc gamma binding protein | protein-coding | 1.2 | 0.050 |
| ENSG00000008853 | RHOBTB2 | Rho related BTB domain containing 2 | protein-coding | 1.2 | 0.012 |
| ENSG00000104814 | MAP4K1 | mitogen-activated protein kinase kinase kinase kinase 1 | protein-coding | 1.2 | 0.021 |
| ENSG00000130653 | PNPLA7 | patatin like phospholipase domain containing 7 | protein-coding | 1.2 | 0.021 |
| ENSG00000103495 | MAZ | MYC associated zinc finger protein | protein-coding | 1.1 | 0.002 |
| ENSG00000122678 | POLM | DNA polymerase mu | protein-coding | 1.1 | 0.006 |
| ENSG00000104529 | EEF1D | eukaryotic translation elongation factor 1 delta | protein-coding | 1.1 | 0.029 |
| ENSG00000198858 | R3HDM4 | R3H domain containing 4 | protein-coding | 1.1 | 0.009 |
| ENSG00000076662 | ICAM3 | intercellular adhesion molecule 3 | protein-coding | 1.1 | 0.041 |
| ENSG00000131196 | NFATC1 | nuclear factor of activated T cells 1 | protein-coding | 1.1 | 0.002 |
| ENSG00000188191 | PRKAR1B | protein kinase cAMP-dependent type I regulatory subunit beta | protein-coding | 1.1 | 0.038 |
| ENSG00000182463 | TSHZ2 | teashirt zinc finger homeobox 2 | protein-coding | 1.1 | 0.042 |
| ENSG00000124575 | H1-3 | H1.3 linker histone. cluster member | protein-coding | 1.1 | 0.017 |
| ENSG00000168067 | MAP4K2 | mitogen-activated protein kinase kinase kinase kinase 2 | protein-coding | 1.1 | 0.012 |
| ENSG00000101945 | SUV39H1 | SUV39H1 histone lysine methyltransferase | protein-coding | 1.1 | 0.023 |
| ENSG00000128340 | RAC2 | Rac family small GTPase 2 | protein-coding | 1.1 | 0.001 |
| ENSG00000100403 | ZC3H7B | zinc finger CCCH-type containing 7B | protein-coding | 1.1 | 0.032 |
| ENSG00000167984 | NLRC3 | NLR family CARD domain containing 3 | protein-coding | 1.1 | 0.015 |
| ENSG00000227507 | LTB | lymphotoxin beta | protein-coding | 1.1 | 0.001 |
| ENSG00000185800 | DMWD | DM1 locus. WD repeat containing | protein-coding | 1.1 | 0.006 |
| ENSG00000188522 | FAM83G | family with sequence similarity 83 member G | protein-coding | 1.1 | 0.037 |
| ENSG00000067057 | PFKP | phosphofructokinase. platelet | protein-coding | 1.1 | 0.013 |
| ENSG00000135631 | RAB11FIP5 | RAB11 family interacting protein 5 | protein-coding | 1.1 | 0.041 |
| ENSG00000232442 | MHENCR | melanoma highly expressed competing endogenous lncRNA for miR-425 and miR-489 | ncRNA | 1.1 | 0.022 |
| ENSG00000088543 | C3orf18 | chromosome 3 open reading frame 18 | protein-coding | 1.1 | 0.001 |
| ENSG00000179588 | ZFPM1 | zinc finger protein. FOG family member 1 | protein-coding | 1.1 | 0.044 |
| ENSG00000173511 | VEGFB | vascular endothelial growth factor B | protein-coding | 1.1 | 0.030 |
| ENSG00000161013 | MGAT4B | alpha-1.3-mannosyl-glycoprotein 4-beta-N-acetylglucosaminyltransferase B | protein-coding | 1.1 | 0.030 |
| ENSG00000139579 | NABP2 | nucleic acid binding protein 2 | protein-coding | 1.1 | <0.001 |
| ENSG00000081692 | JMJD4 | jumonji domain containing 4 | protein-coding | 1.1 | 0.047 |
| ENSG00000172531 | PPP1CA | protein phosphatase 1 catalytic subunit alpha | protein-coding | 1.1 | 0.001 |
| ENSG00000007402 | CACNA2D2 | calcium voltage-gated channel auxiliary subunit alpha2delta 2 | protein-coding | 1.1 | 0.021 |
| ENSG00000173456 | RNF26 | ring finger protein 26 | protein-coding | 1.1 | 0.002 |
| ENSG00000146083 | RNF44 | ring finger protein 44 | protein-coding | 1.1 | 0.034 |
| ENSG00000131116 | ZNF428 | zinc finger protein 428 | protein-coding | 1.1 | 0.005 |
| ENSG00000116685 | KIAA2013 | KIAA2013 | protein-coding | 1.1 | 0.030 |
| ENSG00000243477 | NAA80 | N-alpha-acetyltransferase 80. NatH catalytic subunit | protein-coding | 1.1 | 0.033 |
| ENSG00000213339 | QTRT1 | queuine tRNA-ribosyltransferase catalytic subunit 1 | protein-coding | 1.1 | 0.042 |
| ENSG00000096996 | IL12RB1 | interleukin 12 receptor subunit beta 1 | protein-coding | 1.1 | 0.005 |
| ENSG00000149923 | PPP4C | protein phosphatase 4 catalytic subunit | protein-coding | 1.1 | 0.039 |
| ENSG00000130311 | DDA1 | DET1 and DDB1 associated 1 | protein-coding | 1.1 | 0.013 |
| ENSG00000079999 | KEAP1 | kelch like ECH associated protein 1 | protein-coding | 1.1 | 0.029 |
| ENSG00000104979 | C19orf53 | chromosome 19 open reading frame 53 | protein-coding | 1.1 | 0.017 |
| ENSG00000175463 | TBC1D10C | TBC1 domain family member 10C | protein-coding | 1.1 | 0.034 |
| ENSG00000168646 | AXIN2 | axin 2 | protein-coding | 1.1 | 0.044 |
| ENSG00000124203 | ZNF831 | zinc finger protein 831 | protein-coding | 1.1 | 0.003 |
| ENSG00000175265 | GOLGA8A | golgin A8 family member A | protein-coding | 1.1 | 0.045 |
| ENSG00000104872 | PIH1D1 | PIH1 domain containing 1 | protein-coding | 1.1 | 0.041 |
| ENSG00000162676 | GFI1 | growth factor independent 1 transcriptional repressor | protein-coding | 1.1 | 0.009 |
| ENSG00000167965 | MLST8 | MTOR associated protein. LST8 homolog | protein-coding | 1.1 | 0.047 |
| ENSG00000107816 | LZTS2 | leucine zipper tumor suppressor 2 | protein-coding | 1.1 | 0.024 |
| ENSG00000167491 | GATAD2A | GATA zinc finger domain containing 2A | protein-coding | 1.1 | 0.006 |
| ENSG00000180644 | PRF1 | perforin 1 | protein-coding | 1.1 | 0.019 |
| ENSG00000168286 | THAP11 | THAP domain containing 11 | protein-coding | 1.1 | 0.003 |
| ENSG00000148291 | SURF2 | surfeit 2 | protein-coding | 1.1 | 0.027 |
| ENSG00000178952 | TUFM | Tu translation elongation factor. mitochondrial | protein-coding | 1.1 | 0.010 |
| ENSG00000071655 | MBD3 | methyl-CpG binding domain protein 3 | protein-coding | 1.1 | 0.026 |
| ENSG00000006025 | OSBPL7 | oxysterol binding protein like 7 | protein-coding | 1.1 | 0.031 |
| ENSG00000168298 | H1-4 | H1.4 linker histone. cluster member | protein-coding | 1.1 | 0.039 |
| ENSG00000177732 | SOX12 | SRY-box transcription factor 12 | protein-coding | 1.1 | 0.001 |
| ENSG00000071575 | TRIB2 | tribbles pseudokinase 2 | protein-coding | 1.1 | 0.003 |
| ENSG00000106266 | SNX8 | sorting nexin 8 | protein-coding | 1.1 | 0.032 |
| ENSG00000069188 | SDK2 | sidekick cell adhesion molecule 2 | protein-coding | 1.1 | 0.032 |
| ENSG00000182054 | IDH2 | isocitrate dehydrogenase (NADP(+)) 2 | protein-coding | 1.1 | 0.025 |
| ENSG00000112511 | PHF1 | PHD finger protein 1 | protein-coding | 1.1 | 0.030 |
| ENSG00000164587 | RPS14 | ribosomal protein S14 | protein-coding | 1.1 | <0.001 |
| ENSG00000073861 | TBX21 | T-box transcription factor 21 | protein-coding | 1.1 | 0.033 |
| ENSG00000127418 | FGFRL1 | fibroblast growth factor receptor like 1 | protein-coding | 1.1 | 0.027 |
| ENSG00000247077 | PGAM5 | PGAM family member 5. mitochondrial serine/threonine protein phosphatase | protein-coding | 1.1 | 0.009 |
| ENSG00000275342 | PRAG1 | PEAK1 related. kinase-activating pseudokinase 1 | protein-coding | 1.1 | 0.002 |
| ENSG00000181036 | FCRL6 | Fc receptor like 6 | protein-coding | 1.1 | 0.020 |
| ENSG00000213654 | GPSM3 | G protein signaling modulator 3 | protein-coding | 1.1 | 0.025 |
| ENSG00000185085 | INTS5 | integrator complex subunit 5 | protein-coding | 1.1 | 0.009 |
| ENSG00000111752 | PHC1 | polyhomeotic homolog 1 | protein-coding | 1.1 | 0.035 |
| ENSG00000116824 | CD2 | CD2 molecule | protein-coding | 1.1 | 0.025 |
| ENSG00000089157 | RPLP0 | ribosomal protein lateral stalk subunit P0 | protein-coding | 1.1 | <0.001 |
| ENSG00000107140 | TESK1 | testis associated actin remodelling kinase 1 | protein-coding | 1.1 | 0.041 |
| ENSG00000143761 | ARF1 | ADP ribosylation factor 1 | protein-coding | 1.1 | 0.039 |
| ENSG00000100139 | MICALL1 | MICAL like 1 | protein-coding | 1.1 | 0.026 |
| ENSG00000116670 | MAD2L2 | mitotic arrest deficient 2 like 2 | protein-coding | 1.1 | 0.036 |
| ENSG00000106351 | AGFG2 | ArfGAP with FG repeats 2 | protein-coding | 1.1 | 0.014 |
| ENSG00000164086 | DUSP7 | dual specificity phosphatase 7 | protein-coding | 1.1 | 0.011 |
| ENSG00000137497 | NUMA1 | nuclear mitotic apparatus protein 1 | protein-coding | 1.1 | 0.037 |
| ENSG00000240342 | RPS2P5 | ribosomal protein S2 pseudogene 5 | pseudo | 1.1 | 0.014 |
| ENSG00000173846 | PLK3 | polo like kinase 3 | protein-coding | 1.1 | 0.043 |
| ENSG00000184076 | UQCR10 | ubiquinol-cytochrome c reductase. complex III subunit X | protein-coding | 1.1 | 0.007 |
| ENSG00000103199 | ZNF500 | zinc finger protein 500 | protein-coding | 1.1 | 0.018 |
| ENSG00000085117 | CD82 | CD82 molecule | protein-coding | 1.1 | 0.027 |
| ENSG00000200959 | SNORA74A | small nucleolar RNA. H/ACA box 74A | snoRNA | 1.1 | 0.004 |
| ENSG00000197283 | SYNGAP1 | synaptic Ras GTPase activating protein 1 | protein-coding | 1.1 | 0.003 |
| ENSG00000172057 | ORMDL3 | ORMDL sphingolipid biosynthesis regulator 3 | protein-coding | 1.1 | 0.001 |
| ENSG00000170468 | RIOX1 | ribosomal oxygenase 1 | protein-coding | 1.1 | 0.001 |
| ENSG00000171163 | ZNF692 | zinc finger protein 692 | protein-coding | 1.1 | 0.045 |
| ENSG00000148337 | CIZ1 | CDKN1A interacting zinc finger protein 1 | protein-coding | 1.1 | 0.032 |
| ENSG00000171863 | RPS7 | ribosomal protein S7 | protein-coding | 1.1 | <0.001 |
| ENSG00000141564 | RPTOR | regulatory associated protein of MTOR complex 1 | protein-coding | 1.1 | 0.038 |
| ENSG00000166925 | TSC22D4 | TSC22 domain family member 4 | protein-coding | 1.1 | 0.046 |
| ENSG00000105329 | TGFB1 | transforming growth factor beta 1 | protein-coding | 1.1 | 0.031 |
| ENSG00000136997 | MYC | MYC proto-oncogene. bHLH transcription factor | protein-coding | 1.1 | 0.006 |
| ENSG00000184481 | FOXO4 | forkhead box O4 | protein-coding | 1.1 | 0.005 |
| ENSG00000008988 | RPS20 | ribosomal protein S20 | protein-coding | 1.1 | 0.004 |
| ENSG00000124587 | PEX6 | peroxisomal biogenesis factor 6 | protein-coding | 1.1 | 0.010 |
| ENSG00000270170 | NCBP2AS2 | NCBP2 antisense 2 (head to head) | protein-coding | 1.1 | 0.030 |
| ENSG00000160014 | CALM3 | calmodulin 3 | protein-coding | 1.1 | 0.005 |
| ENSG00000171608 | PIK3CD | phosphatidylinositol-4.5-bisphosphate 3-kinase catalytic subunit delta | protein-coding | 1.1 | 0.036 |
| ENSG00000162241 | SLC25A45 | solute carrier family 25 member 45 | protein-coding | 1.1 | 0.010 |
| ENSG00000057657 | PRDM1 | PR/SET domain 1 | protein-coding | 1.1 | 0.001 |
| ENSG00000204209 | DAXX | death domain associated protein | protein-coding | 1.1 | 0.009 |
| ENSG00000179632 | MAF1 | MAF1 homolog. negative regulator of RNA polymerase III | protein-coding | 1.1 | 0.013 |
| ENSG00000141552 | ANAPC11 | anaphase promoting complex subunit 11 | protein-coding | 1.1 | 0.047 |
| ENSG00000184640 | SEPTIN9 | septin 9 | protein-coding | 1.1 | 0.019 |
| ENSG00000137265 | IRF4 | interferon regulatory factor 4 | protein-coding | 1.1 | 0.028 |
| ENSG00000164054 | SHISA5 | shisa family member 5 | protein-coding | 1.1 | 0.045 |
| ENSG00000185989 | RASA3 | RAS p21 protein activator 3 | protein-coding | 1.1 | 0.018 |
| ENSG00000160445 | ZER1 | zyg-11 related cell cycle regulator | protein-coding | 1.1 | 0.039 |
| ENSG00000106290 | TAF6 | TATA-box binding protein associated factor 6 | protein-coding | 1.1 | 0.026 |
| ENSG00000125637 | PSD4 | pleckstrin and Sec7 domain containing 4 | protein-coding | 1.1 | 0.026 |
| ENSG00000143373 | ZNF687 | zinc finger protein 687 | protein-coding | 1.1 | 0.036 |
| ENSG00000137076 | TLN1 | talin 1 | protein-coding | 1.0 | 0.041 |
| ENSG00000034510 | TMSB10 | thymosin beta 10 | protein-coding | 1.0 | 0.002 |
| ENSG00000173540 | GMPPB | GDP-mannose pyrophosphorylase B | protein-coding | 1.0 | 0.040 |
| ENSG00000103932 | RPAP1 | RNA polymerase II associated protein 1 | protein-coding | 1.0 | 0.018 |
| ENSG00000107485 | GATA3 | GATA binding protein 3 | protein-coding | 1.0 | 0.020 |
| ENSG00000135439 | AGAP2 | ArfGAP with GTPase domain. ankyrin repeat and PH domain 2 | protein-coding | 1.0 | 0.010 |
| ENSG00000101445 | PPP1R16B | protein phosphatase 1 regulatory subunit 16B | protein-coding | 1.0 | 0.009 |
| ENSG00000144749 | LRIG1 | leucine rich repeats and immunoglobulin like domains 1 | protein-coding | 1.0 | 0.002 |
| ENSG00000105618 | PRPF31 | pre-mRNA processing factor 31 | protein-coding | 1.0 | 0.045 |
| ENSG00000131469 | RPL27 | ribosomal protein L27 | protein-coding | 1.0 | <0.001 |
| ENSG00000153406 | NMRAL1 | NmrA like redox sensor 1 | protein-coding | 1.0 | 0.046 |
| ENSG00000155034 | FBXL18 | F-box and leucine rich repeat protein 18 | protein-coding | 1.0 | 0.040 |
| ENSG00000182858 | ALG12 | ALG12 alpha-1.6-mannosyltransferase | protein-coding | 1.0 | <0.001 |
| ENSG00000162244 | RPL29 | ribosomal protein L29 | protein-coding | 1.0 | <0.001 |
| ENSG00000184371 | CSF1 | colony stimulating factor 1 | protein-coding | 1.0 | 0.022 |
| ENSG00000138629 | UBL7 | ubiquitin like 7 | protein-coding | 1.0 | 0.008 |
| ENSG00000140511 | HAPLN3 | hyaluronan and proteoglycan link protein 3 | protein-coding | 1.0 | 0.026 |
| ENSG00000103148 | NPRL3 | NPR3 like. GATOR1 complex subunit | protein-coding | 1.0 | 0.015 |
| ENSG00000055483 | USP36 | ubiquitin specific peptidase 36 | protein-coding | 1.0 | 0.025 |
| ENSG00000050327 | ARHGEF5 | Rho guanine nucleotide exchange factor 5 | protein-coding | 1.0 | 0.026 |
| ENSG00000142002 | DPP9 | dipeptidyl peptidase 9 | protein-coding | 1.0 | 0.046 |
| ENSG00000101216 | GMEB2 | glucocorticoid modulatory element binding protein 2 | protein-coding | 1.0 | 0.014 |
| ENSG00000141858 | SAMD1 | sterile alpha motif domain containing 1 | protein-coding | 1.0 | 0.025 |
| ENSG00000168028 | RPSA | ribosomal protein SA | protein-coding | 1.0 | <0.001 |
| ENSG00000139626 | ITGB7 | integrin subunit beta 7 | protein-coding | 1.0 | 0.002 |
| ENSG00000100413 | POLR3H | RNA polymerase III subunit H | protein-coding | 1.0 | 0.028 |
| ENSG00000185043 | CIB1 | calcium and integrin binding 1 | protein-coding | 1.0 | 0.023 |
| ENSG00000196912 | ANKRD36B | ankyrin repeat domain 36B | protein-coding | 1.0 | 0.003 |
| ENSG00000125651 | GTF2F1 | general transcription factor IIF subunit 1 | protein-coding | 1.0 | 0.015 |
| ENSG00000166313 | APBB1 | amyloid beta precursor protein binding family B member 1 | protein-coding | 1.0 | 0.003 |
| ENSG00000075234 | TTC38 | tetratricopeptide repeat domain 38 | protein-coding | 1.0 | 0.015 |
| ENSG00000126882 | FAM78A | family with sequence similarity 78 member A | protein-coding | 1.0 | 0.020 |
| ENSG00000198873 | GRK5 | G protein-coupled receptor kinase 5 | protein-coding | 1.0 | 0.012 |
| ENSG00000075624 | ACTB | actin beta | protein-coding | 1.0 | 0.022 |
| ENSG00000179715 | PCED1B | PC-esterase domain containing 1B | protein-coding | 1.0 | 0.027 |
| ENSG00000043143 | JADE2 | jade family PHD finger 2 | protein-coding | 1.0 | 0.001 |
| ENSG00000182199 | SHMT2 | serine hydroxymethyltransferase 2 | protein-coding | 1.0 | 0.032 |
| ENSG00000104825 | NFKBIB | NFKB inhibitor beta | protein-coding | 1.0 | 0.035 |
| ENSG00000108961 | RANGRF | RAN guanine nucleotide release factor | protein-coding | 1.0 | 0.002 |
| ENSG00000290600 |  | novel transcript | ncRNA | 1.0 | 0.034 |
| ENSG00000119669 | IRF2BPL | interferon regulatory factor 2 binding protein like | protein-coding | 1.0 | 0.007 |
| ENSG00000100982 | PCIF1 | phosphorylated CTD interacting factor 1 | protein-coding | 1.0 | 0.047 |
| ENSG00000107175 | CREB3 | cAMP responsive element binding protein 3 | protein-coding | 1.0 | 0.029 |
| ENSG00000178252 | WDR6 | WD repeat domain 6 | protein-coding | 1.0 | 0.017 |
| ENSG00000143515 | ATP8B2 | ATPase phospholipid transporting 8B2 | protein-coding | 1.0 | 0.025 |
| ENSG00000126456 | IRF3 | interferon regulatory factor 3 | protein-coding | 1.0 | 0.050 |
| ENSG00000104884 | ERCC2 | ERCC excision repair 2. TFIIH core complex helicase subunit | protein-coding | 1.0 | 0.033 |
| ENSG00000102226 | USP11 | ubiquitin specific peptidase 11 | protein-coding | 1.0 | 0.001 |
| ENSG00000175634 | RPS6KB2 | ribosomal protein S6 kinase B2 | protein-coding | 1.0 | 0.039 |
| ENSG00000144152 | FBLN7 | fibulin 7 | protein-coding | 1.0 | 0.036 |
| ENSG00000139722 | VPS37B | VPS37B subunit of ESCRT-I | protein-coding | 1.0 | 0.001 |
| ENSG00000148303 | RPL7A | ribosomal protein L7a | protein-coding | 1.0 | <0.001 |
| ENSG00000112033 | PPARD | peroxisome proliferator activated receptor delta | protein-coding | 1.0 | 0.041 |
| ENSG00000104880 | ARHGEF18 | Rho/Rac guanine nucleotide exchange factor 18 | protein-coding | 1.0 | 0.047 |
| ENSG00000100324 | TAB1 | TGF-beta activated kinase 1 (MAP3K7) binding protein 1 | protein-coding | 1.0 | 0.041 |
| ENSG00000275023 | MLLT6 | MLLT6. PHD finger containing | protein-coding | 1.0 | 0.005 |
| ENSG00000132182 | NUP210 | nucleoporin 210 | protein-coding | 1.0 | 0.017 |
| ENSG00000114942 | EEF1B2 | eukaryotic translation elongation factor 1 beta 2 | protein-coding | 1.0 | 0.015 |

**FC = fold change, p-adj = FDR-adjusted p-value**

**Table S3:** Significantly downregulated RNAs at gestational weeks 36-38 in women developing preeclampsia (N=4) vs normal pregnancy (N=6).

| **Ensembl** | **Symbol** | **Description** | **Biotype** | **log_2_FC** | **p-adj** |
| --- | --- | --- | --- | --- | --- |
| ENSG00000259379 | MTND5P32 | MT-ND5 pseudogene 32 | pseudo | -2.7 | <0.001 |
| ENSG00000289310 |  | novel transcript. sense intronic to LRRK2 | ncRNA | -2.6 | 0.001 |
| ENSG00000259671 | MTCYBP23 | MT-CYB pseudogene 23 | pseudo | -2.6 | <0.001 |
| ENSG00000259600 | MTND4LP23 | MT-ND4L pseudogene 23 | pseudo | -2.5 | 0.001 |
| ENSG00000137959 | IFI44L | interferon induced protein 44 like | protein-coding | -2.3 | 0.001 |
| ENSG00000229899 |  | pseudogene similar to part of the cytochrome c oxidase subunit I (COX1) | pseudo | -2.2 | 0.042 |
| ENSG00000230789 | ARHGAP26-IT1 | ARHGAP26 intronic transcript 1 | ncRNA | -2.0 | 0.010 |
| ENSG00000272807 |  | novel transcript. antisense to KANSL1L | ncRNA | -1.9 | 0.034 |
| ENSG00000232542 | DPYD-IT1 | DPYD intronic transcript 1 | ncRNA | -1.8 | 0.001 |
| ENSG00000228097 | MTATP6P11 | MT-ATP6 pseudogene 11 | pseudo | -1.7 | 0.002 |
| ENSG00000276015 |  |  | ncRNA | -1.7 | <0.001 |
| ENSG00000204020 | LIPN | lipase family member N | protein-coding | -1.7 | <0.001 |
| ENSG00000286219 | NOTCH2NLC | notch 2 N-terminal like C | protein-coding | -1.7 | <0.001 |
| ENSG00000237272 | OR51R1P | olfactory receptor family 51 subfamily R member 1 pseudogene | pseudo | -1.7 | 0.003 |
| ENSG00000239040 |  | Y RNA | ncRNA | -1.6 | 0.043 |
| ENSG00000154589 | LY96 | lymphocyte antigen 96 | protein-coding | -1.6 | 0.019 |
| ENSG00000185745 | IFIT1 | interferon induced protein with tetratricopeptide repeats 1 | protein-coding | -1.6 | 0.005 |
| ENSG00000258476 | LINC02207 | long intergenic non-protein coding RNA 2207 | ncRNA | -1.6 | 0.009 |
| ENSG00000279663 |  | TEC | TEC | -1.6 | <0.001 |
| ENSG00000236254 | MTND4P14 | MT-ND4 pseudogene 14 | pseudo | -1.6 | 0.014 |
| ENSG00000279957 |  | TEC | TEC | -1.5 | 0.035 |
| ENSG00000166527 | CLEC4D | C-type lectin domain family 4 member D | protein-coding | -1.5 | 0.005 |
| ENSG00000232373 | MTCYBP3 | MT-CYB pseudogene 3 | pseudo | -1.5 | 0.044 |
| ENSG00000277130 |  | novel transcript. antisense to PPP1R12A | ncRNA | -1.5 | 0.005 |
| ENSG00000279447 |  | TEC | TEC | -1.5 | 0.026 |
| ENSG00000270062 |  | novel transcript. sense intronic to NIN | ncRNA | -1.5 | 0.019 |
| ENSG00000205571 | SMN1 | survival of motor neuron 1. telomeric | protein-coding | -1.5 | 0.009 |
| ENSG00000286019 | NOTCH2NLB | notch 2 N-terminal like B | protein-coding | -1.5 | 0.001 |
| ENSG00000228166 | MTND1P11 | MT-ND1 pseudogene 11 | pseudo | -1.5 | 0.015 |
| ENSG00000291144 |  | novel transcript | ncRNA | -1.5 | 0.004 |
| ENSG00000255163 | HSPE1P18 | heat shock protein family E (Hsp10) member 1 pseudogene 18 | pseudo | -1.5 | 0.001 |
| ENSG00000143452 | HORMAD1 | HORMA domain containing 1 | protein-coding | -1.5 | 0.006 |
| ENSG00000273284 |  | novel transcript. sense intronic to ANKRD12 | ncRNA | -1.5 | 0.011 |
| ENSG00000115271 | GCA | grancalcin | protein-coding | -1.5 | 0.002 |
| ENSG00000196778 | OR52K1 | olfactory receptor family 52 subfamily K member 1 | protein-coding | -1.5 | 0.006 |
| ENSG00000241772 |  |  |  | -1.5 | 0.038 |
| ENSG00000224707 | E2F3-IT1 | E2F3 intronic transcript 1 | ncRNA | -1.5 | 0.015 |
| ENSG00000237135 | DDX10P1 | DEAD-box helicase 10 pseudogene 1 | pseudo | -1.4 | <0.001 |
| ENSG00000124882 | EREG | epiregulin | protein-coding | -1.4 | 0.037 |
| ENSG00000251429 | AIDAP2 | AIDA pseudogene 2 | pseudo | -1.4 | 0.025 |
| ENSG00000248429 | GASK1B-AS1 | GASK1B antisense RNA 1 | ncRNA | -1.4 | 0.001 |
| ENSG00000164124 | TMEM144 | transmembrane protein 144 | protein-coding | -1.4 | <0.001 |
| ENSG00000279320 |  | TEC | TEC | -1.4 | 0.006 |
| ENSG00000250608 | NUDT16-DT | NUDT16 divergent transcript | ncRNA | -1.4 | 0.002 |
| ENSG00000277151 |  | novel transcript | ncRNA | -1.4 | 0.014 |
| ENSG00000236709 | DAPK1-IT1 | DAPK1 intronic transcript 1 | ncRNA | -1.4 | 0.025 |
| ENSG00000266126 |  | novel transcript | ncRNA | -1.4 | 0.036 |
| ENSG00000289617 |  | novel transcript. sense intronic to FRY | ncRNA | -1.4 | 0.031 |
| ENSG00000101883 | RHOXF1 | Rhox homeobox family member 1 | protein-coding | -1.4 | 0.044 |
| ENSG00000255893 |  | novel transcript | ncRNA | -1.3 | 0.010 |
| ENSG00000289470 |  | novel transcript | ncRNA | -1.3 | 0.001 |
| ENSG00000166507 | NDST2 | N-deacetylase and N-sulfotransferase 2 | protein-coding | -1.3 | 0.012 |
| ENSG00000270562 | FOXP1-DT | FOXP1 divergent transcript | ncRNA | -1.3 | 0.024 |
| ENSG00000093134 | VNN3P | vanin 3. pseudogene | pseudo | -1.3 | 0.009 |
| ENSG00000289691 |  | novel transcript | ncRNA | -1.3 | <0.001 |
| ENSG00000086288 | NME8 | NME/NM23 family member 8 | protein-coding | -1.3 | 0.009 |
| ENSG00000186205 | MTARC1 | mitochondrial amidoxime reducing component 1 | protein-coding | -1.3 | 0.016 |
| ENSG00000121552 | CSTA | cystatin A | protein-coding | -1.3 | 0.030 |
| ENSG00000151773 | CCDC122 | coiled-coil domain containing 122 | protein-coding | -1.3 | 0.002 |
| ENSG00000267632 |  | novel transcript | ncRNA | -1.3 | 0.022 |
| ENSG00000256660 | CLEC12B | C-type lectin domain family 12 member B | protein-coding | -1.3 | 0.002 |
| ENSG00000105967 | TFEC | transcription factor EC | protein-coding | -1.3 | 0.001 |
| ENSG00000230225 | MTND5P14 | MT-ND5 pseudogene 14 | pseudo | -1.3 | 0.028 |
| ENSG00000137965 | IFI44 | interferon induced protein 44 | protein-coding | -1.3 | 0.041 |
| ENSG00000266127 | ZNF415P1 | zinc finger protein 415 pseudogene 1 | pseudo | -1.3 | 0.020 |
| ENSG00000276533 |  | novel transcript. sense intronic to RAB27A | ncRNA | -1.3 | 0.005 |
| ENSG00000205534 | SMG1P2 | SMG1 pseudogene 2 | pseudo | -1.3 | 0.037 |
| ENSG00000271862 |  | novel trancript | ncRNA | -1.3 | 0.004 |
| ENSG00000284977 | LOC105376244 | uncharacterized LOC105376244 | ncRNA | -1.2 | 0.023 |
| ENSG00000235917 | MTCO2P11 | MT-CO2 pseudogene 11 | pseudo | -1.2 | 0.026 |
| ENSG00000187037 | GPR141 | G protein-coupled receptor 141 | protein-coding | -1.2 | 0.001 |
| ENSG00000276649 |  |  |  | -1.2 | 0.006 |
| ENSG00000275056 |  | novel transcript. sense intronic to GLYR1 | ncRNA | -1.2 | 0.001 |
| ENSG00000265625 |  | novel transcript. sense intronic to SSH2 | ncRNA | -1.2 | <0.001 |
| ENSG00000117151 | CTBS | chitobiase | protein-coding | -1.2 | 0.002 |
| ENSG00000244687 | UBE2V1 | ubiquitin conjugating enzyme E2 V1 | protein-coding | -1.2 | 0.009 |
| ENSG00000224083 | MTCO1P11 | MT-CO1 pseudogene 11 | pseudo | -1.2 | 0.006 |
| ENSG00000151151 | IPMK | inositol polyphosphate multikinase | protein-coding | -1.2 | 0.004 |
| ENSG00000288025 |  | novel transcript. antisense to SCRG1 | ncRNA | -1.2 | 0.001 |
| ENSG00000288538 |  | novel transcript. antisense to KCTD21 | ncRNA | -1.2 | 0.003 |
| ENSG00000288899 |  | novel transcript | ncRNA | -1.2 | 0.048 |
| ENSG00000229989 | MIR181A1HG | MIR181A1 host gene | ncRNA | -1.2 | 0.002 |
| ENSG00000179630 | LACC1 | laccase domain containing 1 | protein-coding | -1.2 | 0.050 |
| ENSG00000172322 | CLEC12A | C-type lectin domain family 12 member A | protein-coding | -1.2 | 0.018 |
| ENSG00000162959 | MEMO1 | mediator of cell motility 1 | protein-coding | -1.2 | 0.024 |
| ENSG00000271533 |  | novel transcript. sense intronic FTX | ncRNA | -1.2 | 0.001 |
| ENSG00000289251 |  | novel transcript | ncRNA | -1.2 | 0.038 |
| ENSG00000289013 |  | novel transcript. sense intronic to SLC8A1 | ncRNA | -1.2 | 0.027 |
| ENSG00000235530 | TMEM11-DT | TMEM11 divergent transcript | ncRNA | -1.2 | 0.024 |
| ENSG00000271383 | NBPF19 | NBPF member 19 | protein-coding | -1.2 | 0.009 |
| ENSG00000289463 |  | novel transcript. sense intronic to ZNF638 | ncRNA | -1.2 | 0.019 |
| ENSG00000072401 | UBE2D1 | ubiquitin conjugating enzyme E2 D1 | protein-coding | -1.2 | 0.041 |
| ENSG00000290792 |  | novel transcript | ncRNA | -1.2 | 0.005 |
| ENSG00000226761 | TAS2R46 | taste 2 receptor member 46 | protein-coding | -1.2 | 0.048 |
| ENSG00000250461 |  | family with sequence similarity 133. member B (FAM133B) pseudogene | pseudo | -1.2 | 0.019 |
| ENSG00000266900 |  | novel transcript. sense intronic to KIAA1468 | ncRNA | -1.2 | 0.026 |
| ENSG00000280543 | ASAP1-IT2 | ASAP1 intronic transcript 2 | ncRNA | -1.2 | 0.003 |
| ENSG00000277687 |  | novel transcript. sense intronic to CACUL1 | ncRNA | -1.2 | 0.001 |
| ENSG00000243650 | RN7SL834P | RNA. 7SL. cytoplasmic 834. pseudogene | pseudo | -1.2 | 0.002 |
| ENSG00000274536 | MIR223HG | MIR223 host gene | ncRNA | -1.2 | 0.009 |
| ENSG00000275764 |  | novel transcript | ncRNA | -1.2 | 0.047 |
| ENSG00000150048 | CLEC1A | C-type lectin domain family 1 member A | protein-coding | -1.1 | 0.036 |
| ENSG00000179978 | NAIPP2 | NAIP pseudogene 2 | pseudo | -1.1 | 0.034 |
| ENSG00000264343 | NOTCH2NLA | notch 2 N-terminal like A | protein-coding | -1.1 | 0.003 |
| ENSG00000279814 |  | TEC | TEC | -1.1 | 0.008 |
| ENSG00000289353 |  | novel transcript. sense intronic to CMTM1and CKLF-CMTM1 | ncRNA | -1.1 | 0.049 |
| ENSG00000143067 | ZNF697 | zinc finger protein 697 | protein-coding | -1.1 | 0.010 |
| ENSG00000138646 | HERC5 | HECT and RLD domain containing E3 ubiquitin protein ligase 5 | protein-coding | -1.1 | 0.007 |
| ENSG00000279620 |  | novel transcript | TEC | -1.1 | 0.039 |
| ENSG00000286825 |  |  |  | -1.1 | 0.004 |
| ENSG00000238113 | LINC01410 | long intergenic non-protein coding RNA 1410 | ncRNA | -1.1 | 0.029 |
| ENSG00000237711 | MTCO3P11 | MT-CO3 pseudogene 11 | pseudo | -1.1 | 0.018 |
| ENSG00000274963 | RN7SL600P | RNA. 7SL. cytoplasmic 600. pseudogene | pseudo | -1.1 | 0.019 |
| ENSG00000181619 | GPR135 | G protein-coupled receptor 135 | protein-coding | -1.1 | 0.040 |
| ENSG00000231607 | DLEU2 | deleted in lymphocytic leukemia 2 | ncRNA | -1.1 | 0.015 |
| ENSG00000281026 | N4BP2L2-IT2 | N4BPL2 intronic transcript 2 | ncRNA | -1.1 | 0.006 |
| ENSG00000175857 | GAPT | GRB2 binding adaptor protein. transmembrane | protein-coding | -1.1 | 0.027 |
| ENSG00000236535 | RC3H1-IT1 | RC3H1 intronic transcript 1 | ncRNA | -1.1 | 0.010 |
| ENSG00000164136 | IL15 | interleukin 15 | protein-coding | -1.1 | 0.005 |
| ENSG00000119922 | IFIT2 | interferon induced protein with tetratricopeptide repeats 2 | protein-coding | -1.1 | 0.032 |
| ENSG00000230539 | AOAH-IT1 | AOAH intronic transcript 1 | ncRNA | -1.1 | 0.008 |
| ENSG00000269924 |  | novel transcript. sense intronic to KIAA0146 | ncRNA | -1.1 | 0.014 |
| ENSG00000290683 |  | novel transcript | ncRNA | -1.1 | 0.002 |
| ENSG00000062716 | VMP1 | vacuole membrane protein 1 | protein-coding | -1.1 | 0.006 |
| ENSG00000228280 |  | novel transcript. sense intronic to C10orf11 | ncRNA | -1.1 | 0.050 |
| ENSG00000232973 | CYP1B1-AS1 | CYP1B1 antisense RNA 1 | ncRNA | -1.1 | 0.015 |
| ENSG00000229152 |  | ANKRD10 intronic transcript 1 | ncRNA | -1.1 | 0.041 |
| ENSG00000090376 | IRAK3 | interleukin 1 receptor associated kinase 3 | protein-coding | -1.1 | 0.004 |
| ENSG00000279166 |  | TEC | TEC | -1.1 | 0.014 |
| ENSG00000107201 | RIGI | RNA sensor RIG-I | protein-coding | -1.1 | <0.001 |
| ENSG00000166927 | MS4A7 | membrane spanning 4-domains A7 | protein-coding | -1.1 | 0.041 |
| ENSG00000273691 |  | novel transcript. sense intronic to ZNF710 | ncRNA | -1.1 | 0.017 |
| ENSG00000254325 |  | novel transcript | ncRNA | -1.1 | 0.025 |
| ENSG00000224003 | YES1P1 | YES1 pseudogene 1 | pseudo | -1.1 | 0.002 |
| ENSG00000272888 | CHASERR | CHD2 adjacent suppressive regulatory RNA | ncRNA | -1.1 | 0.010 |
| ENSG00000270344 | POC1B-AS1 | POC1B antisense RNA 1 | ncRNA | -1.1 | 0.009 |
| ENSG00000111666 | CHPT1 | choline phosphotransferase 1 | protein-coding | -1.0 | <0.001 |
| ENSG00000101916 | TLR8 | toll like receptor 8 | protein-coding | -1.0 | 0.043 |
| ENSG00000115919 | KYNU | kynureninase | protein-coding | -1.0 | 0.002 |
| ENSG00000254996 | ANKHD1-EIF4EBP3 | ANKHD1-EIF4EBP3 readthrough | protein-coding | -1.0 | 0.022 |
| ENSG00000258944 |  | novel transcript. antisense to PAPLN | ncRNA | -1.0 | 0.025 |
| ENSG00000228817 | BACH1-IT2 | BACH1 intronic transcript 2 | ncRNA | -1.0 | 0.042 |
| ENSG00000242094 | FOXP1-IT1 | FOXP1 intronic transcript 1 | ncRNA | -1.0 | 0.001 |
| ENSG00000283839 | LOC105373780 | proline-rich protein 18-like | ncRNA | -1.0 | 0.017 |
| ENSG00000147894 | C9orf72 | C9orf72-SMCR8 complex subunit | protein-coding | -1.0 | 0.016 |
| ENSG00000236194 |  | novel transcript. sense intronic to STAT5B | ncRNA | -1.0 | 0.029 |
| ENSG00000287100 |  | novel transcript | ncRNA | -1.0 | 0.023 |
| ENSG00000288839 |  | novel transcript. sense intronic to RNMT | ncRNA | -1.0 | 0.008 |
| ENSG00000151458 | ANKRD50 | ankyrin repeat domain containing 50 | protein-coding | -1.0 | 0.019 |
| ENSG00000205269 | TMEM170B | transmembrane protein 170B | protein-coding | -1.0 | 0.004 |
| ENSG00000039319 | ZFYVE16 | zinc finger FYVE-type containing 16 | protein-coding | -1.0 | <0.001 |
| ENSG00000203666 | EFCAB2 | EF-hand calcium binding domain 2 | protein-coding | -1.0 | 0.012 |
| ENSG00000261596 |  | novel transcript. sense intronic to METTL9 | ncRNA | -1.0 | 0.025 |
| ENSG00000169629 | RGPD8 | RANBP2 like and GRIP domain containing 8 | protein-coding | -1.0 | 0.017 |
| ENSG00000180509 | KCNE1 | potassium voltage-gated channel subfamily E regulatory subunit 1 | protein-coding | -1.0 | 0.042 |

**FC = fold change, p-adj = FDR-adjusted p-value**

**Table S4:** Significantly upregulated and downregulated RNAs 5 years following pregnancy in women who had preeclampsia (N=9) vs normal pregnancy (N=7).

| **Ensembl** | **Symbol** | **Description** | **Biotype** | **log_2_FC** | **p-adj** |
| --- | --- | --- | --- | --- | --- |
| ENSG00000243238 | IGKV2-30 | immunoglobulin kappa variable 2-30 | other | 2.2 | 0.026 |
| ENSG00000173262 | SLC2A14 | solute carrier family 2 member 14 | protein-coding | -4.9 | 0.004 |
| ENSG00000233429 | HOTAIRM1 | HOXA transcript antisense RNA. myeloid-specific 1 | ncRNA | -1.4 | 0.026 |

**FC = fold change, p-adj = FDR-adjusted p-value**

**Table S5:** Significantly regulated RNAs at 36—38 weeks (N=6 controls, N=4 preeclampsia) involved in each Reactome pathway analysis term for the top 10 upregulated and downregulated pathways.

| **group** | **n** | **Pathway** | **Pathway ID** | **RNAs** |
| --- | --- | --- | --- | --- |
| Up | 31 | Eukaryotic Translation Elongation | R-HSA-156842 | ENSG00000008988, ENSG00000083845, ENSG00000089157, ENSG00000100316, ENSG00000104529, ENSG00000105193, ENSG00000105372, ENSG00000105640, ENSG00000108107, ENSG00000108298, ENSG00000112306, ENSG00000114942, ENSG00000115268, ENSG00000130255, ENSG00000131469, ENSG00000136942, ENSG00000137818, ENSG00000140988, ENSG00000142541, ENSG00000148303, ENSG00000149273, ENSG00000161016, ENSG00000162244, ENSG00000164587, ENSG00000167526, ENSG00000167658, ENSG00000168028, ENSG00000171858, ENSG00000171863, ENSG00000177600, ENSG00000231500 |
| Up | 29 | Peptide chain elongation | R-HSA-156902 | ENSG00000008988, ENSG00000083845, ENSG00000089157, ENSG00000100316, ENSG00000105193, ENSG00000105372, ENSG00000105640, ENSG00000108107, ENSG00000108298, ENSG00000112306, ENSG00000115268, ENSG00000130255, ENSG00000131469, ENSG00000136942, ENSG00000137818, ENSG00000140988, ENSG00000142541, ENSG00000148303, ENSG00000149273, ENSG00000161016, ENSG00000162244, ENSG00000164587, ENSG00000167526, ENSG00000167658, ENSG00000168028, ENSG00000171858, ENSG00000171863, ENSG00000177600, ENSG00000231500 |
| Up | 28 | Viral mRNA Translation | R-HSA-192823 | ENSG00000008988, ENSG00000083845, ENSG00000089157, ENSG00000100316, ENSG00000105193, ENSG00000105372, ENSG00000105640, ENSG00000108107, ENSG00000108298, ENSG00000112306, ENSG00000115268, ENSG00000130255, ENSG00000131469, ENSG00000136942, ENSG00000137818, ENSG00000140988, ENSG00000142541, ENSG00000148303, ENSG00000149273, ENSG00000161016, ENSG00000162244, ENSG00000164587, ENSG00000167526, ENSG00000168028, ENSG00000171858, ENSG00000171863, ENSG00000177600, ENSG00000231500 |
| Up | 29 | Formation of a pool of free 40S subunits | R-HSA-72689 | ENSG00000008988, ENSG00000083845, ENSG00000089157, ENSG00000100316, ENSG00000105193, ENSG00000105372, ENSG00000105640, ENSG00000108107, ENSG00000108298, ENSG00000112306, ENSG00000115268, ENSG00000130255, ENSG00000130811, ENSG00000131469, ENSG00000136942, ENSG00000137818, ENSG00000140988, ENSG00000142541, ENSG00000148303, ENSG00000149273, ENSG00000161016, ENSG00000162244, ENSG00000164587, ENSG00000167526, ENSG00000168028, ENSG00000171858, ENSG00000171863, ENSG00000177600, ENSG00000231500 |
| Up | 28 | Eukaryotic Translation Termination | R-HSA-72764 | ENSG00000008988, ENSG00000083845, ENSG00000089157, ENSG00000100316, ENSG00000105193, ENSG00000105372, ENSG00000105640, ENSG00000108107, ENSG00000108298, ENSG00000112306, ENSG00000115268, ENSG00000130255, ENSG00000131469, ENSG00000136942, ENSG00000137818, ENSG00000140988, ENSG00000142541, ENSG00000148303, ENSG00000149273, ENSG00000161016, ENSG00000162244, ENSG00000164587, ENSG00000167526, ENSG00000168028, ENSG00000171858, ENSG00000171863, ENSG00000177600, ENSG00000231500 |
| Up | 28 | Selenocysteine synthesis | R-HSA-2408557 | ENSG00000008988, ENSG00000083845, ENSG00000089157, ENSG00000100316, ENSG00000105193, ENSG00000105372, ENSG00000105640, ENSG00000108107, ENSG00000108298, ENSG00000112306, ENSG00000115268, ENSG00000130255, ENSG00000131469, ENSG00000136942, ENSG00000137818, ENSG00000140988, ENSG00000142541, ENSG00000148303, ENSG00000149273, ENSG00000161016, ENSG00000162244, ENSG00000164587, ENSG00000167526, ENSG00000168028, ENSG00000171858, ENSG00000171863, ENSG00000177600, ENSG00000231500 |
| Up | 28 | Nonsense Mediated Decay NMD independent of the Exon Junction Complex EJC | R-HSA-975956 | ENSG00000008988, ENSG00000083845, ENSG00000089157, ENSG00000100316, ENSG00000105193, ENSG00000105372, ENSG00000105640, ENSG00000108107, ENSG00000108298, ENSG00000112306, ENSG00000115268, ENSG00000130255, ENSG00000131469, ENSG00000136942, ENSG00000137818, ENSG00000140988, ENSG00000142541, ENSG00000148303, ENSG00000149273, ENSG00000161016, ENSG00000162244, ENSG00000164587, ENSG00000167526, ENSG00000168028, ENSG00000171858, ENSG00000171863, ENSG00000177600, ENSG00000231500 |
| Up | 29 | L13a-mediated translational silencing of Ceruloplasmin expression | R-HSA-156827 | ENSG00000008988, ENSG00000083845, ENSG00000089157, ENSG00000100316, ENSG00000105193, ENSG00000105372, ENSG00000105640, ENSG00000108107, ENSG00000108298, ENSG00000112306, ENSG00000115268, ENSG00000130255, ENSG00000130811, ENSG00000131469, ENSG00000136942, ENSG00000137818, ENSG00000140988, ENSG00000142541, ENSG00000148303, ENSG00000149273, ENSG00000161016, ENSG00000162244, ENSG00000164587, ENSG00000167526, ENSG00000168028, ENSG00000171858, ENSG00000171863, ENSG00000177600, ENSG00000231500 |
| Up | 28 | Response of EIF2AK4 GCN2 to amino acid deficiency | R-HSA-9633012 | ENSG00000008988, ENSG00000083845, ENSG00000089157, ENSG00000100316, ENSG00000105193, ENSG00000105372, ENSG00000105640, ENSG00000108107, ENSG00000108298, ENSG00000112306, ENSG00000115268, ENSG00000130255, ENSG00000131469, ENSG00000136942, ENSG00000137818, ENSG00000140988, ENSG00000142541, ENSG00000148303, ENSG00000149273, ENSG00000161016, ENSG00000162244, ENSG00000164587, ENSG00000167526, ENSG00000168028, ENSG00000171858, ENSG00000171863, ENSG00000177600, ENSG00000231500 |
| Up | 29 | GTP hydrolysis and joining of the 60S ribosomal subunit | R-HSA-72706 | ENSG00000008988, ENSG00000083845, ENSG00000089157, ENSG00000100316, ENSG00000105193, ENSG00000105372, ENSG00000105640, ENSG00000108107, ENSG00000108298, ENSG00000112306, ENSG00000115268, ENSG00000130255, ENSG00000130811, ENSG00000131469, ENSG00000136942, ENSG00000137818, ENSG00000140988, ENSG00000142541, ENSG00000148303, ENSG00000149273, ENSG00000161016, ENSG00000162244, ENSG00000164587, ENSG00000167526, ENSG00000168028, ENSG00000171858, ENSG00000171863, ENSG00000177600, ENSG00000231500 |
| Down | 3 | IKK complex recruitment mediated by RIP1 | R-HSA-937041 | ENSG00000072401, ENSG00000154589, ENSG00000244687 |
| Down | 5 | Toll-like Receptor Cascades | R-HSA-168898 | ENSG00000072401, ENSG00000090376, ENSG00000101916, ENSG00000154589, ENSG00000244687 |
| Down | 3 | Negative regulators of DDX58/IFIH1 signaling | R-HSA-936440 | ENSG00000072401, ENSG00000107201, ENSG00000138646 |
| Down | 10 | Innate Immune System | R-HSA-168249 | ENSG00000072401, ENSG00000090376, ENSG00000101916, ENSG00000107201, ENSG00000115271, ENSG00000138646, ENSG00000154589, ENSG00000166527, ENSG00000172322, ENSG00000244687 |
| Down | 4 | Toll Like Receptor 4 TLR4 Cascade | R-HSA-166016 | ENSG00000072401, ENSG00000090376, ENSG00000154589, ENSG00000244687 |
| Down | 3 | ISG15 antiviral mechanism | R-HSA-1169408 | ENSG00000107201, ENSG00000138646, ENSG00000185745 |
| Down | 2 | ERBB2 Regulates Cell Motility | R-HSA-6785631 | ENSG00000124882, ENSG00000162959 |
| Down | 8 | Cytokine Signaling in Immune system | R-HSA-1280215 | ENSG00000072401, ENSG00000090376, ENSG00000107201, ENSG00000119922, ENSG00000138646, ENSG00000164136, ENSG00000185745, ENSG00000244687 |
| Down | 2 | TICAM1 RIP1-mediated IKK complex recruitment | R-HSA-168927 | ENSG00000072401, ENSG00000244687 |
| Down | 3 | Antiviral mechanism by IFN-stimulated genes | R-HSA-1169410 | ENSG00000107201, ENSG00000138646, ENSG00000185745 |

**Table S6:** Significantly regulated RNAs at 36—38 weeks (N=6 controls, N=4 preeclampsia) involved in each Gene Ontology (Biological Process) enrichment term for the top 15 upregulated and downregulated pathways.

| **group** | **n** | **Pathway** | **GO term** | **RNAs** |
| --- | --- | --- | --- | --- |
| Up | 30 | Cytoplasmic translation | GO:0002181 | ENSG00000089157, ENSG00000130255, ENSG00000137818, ENSG00000161016, ENSG00000162244, ENSG00000168028, ENSG00000008988, ENSG00000083845, ENSG00000100316, ENSG00000105193, ENSG00000105372, ENSG00000105640, ENSG00000108107, ENSG00000108298, ENSG00000112306, ENSG00000115268, ENSG00000130811, ENSG00000131469, ENSG00000136942, ENSG00000140988, ENSG00000142541, ENSG00000148303, ENSG00000149273, ENSG00000164587, ENSG00000167526, ENSG00000171858, ENSG00000171863, ENSG00000177600, ENSG00000204628, ENSG00000231500 |
| Up | 110 | Intracellular signal transduction | GO:0035556 | ENSG00000104529, ENSG00000164054, ENSG00000196924, ENSG00000056558, ENSG00000068831, ENSG00000099875, ENSG00000103148, ENSG00000104856, ENSG00000104880, ENSG00000128340, ENSG00000131196, ENSG00000141522, ENSG00000141564, ENSG00000144476, ENSG00000158050, ENSG00000164086, ENSG00000164867, ENSG00000167100, ENSG00000167965, ENSG00000167984, ENSG00000171608, ENSG00000173846, ENSG00000175634, ENSG00000179526, ENSG00000185189, ENSG00000186810, ENSG00000198286, ENSG00000274070, ENSG00000160014, ENSG00000008988, ENSG00000050327, ENSG00000074370, ENSG00000090975, ENSG00000103495, ENSG00000104814, ENSG00000106290, ENSG00000107175, ENSG00000109062, ENSG00000110651, ENSG00000111640, ENSG00000115268, ENSG00000116670, ENSG00000131653, ENSG00000132819, ENSG00000136997, ENSG00000139579, ENSG00000149273, ENSG00000168067, ENSG00000168758, ENSG00000173511, ENSG00000182866, ENSG00000185043, ENSG00000204209, ENSG00000205336, ENSG00000008853, ENSG00000037280, ENSG00000096433, ENSG00000100324, ENSG00000104825, ENSG00000105373, ENSG00000106211, ENSG00000107485, ENSG00000110711, ENSG00000112033, ENSG00000114737, ENSG00000115085, ENSG00000124181, ENSG00000125637, ENSG00000126456, ENSG00000135439, ENSG00000139193, ENSG00000139725, ENSG00000149923, ENSG00000158856, ENSG00000166289, ENSG00000166341, ENSG00000167815, ENSG00000175463, ENSG00000184371, ENSG00000184381, ENSG00000184481, ENSG00000185989, ENSG00000186716, ENSG00000197283, ENSG00000197457, ENSG00000197562, ENSG00000061273, ENSG00000062524, ENSG00000064545, ENSG00000101445, ENSG00000101997, ENSG00000104872, ENSG00000105122, ENSG00000108961, ENSG00000120885, ENSG00000126457, ENSG00000130726, ENSG00000132382, ENSG00000161999, ENSG00000171863, ENSG00000204628, ENSG00000213626, ENSG00000275342, ENSG00000130175, ENSG00000087074, ENSG00000105519, ENSG00000105701, ENSG00000106683, ENSG00000143851, ENSG00000154016 |
| Up | 47 | Translation | GO:0006412 | ENSG00000081692, ENSG00000083845, ENSG00000089157, ENSG00000104529, ENSG00000114942, ENSG00000130255, ENSG00000137818, ENSG00000140988, ENSG00000142541, ENSG00000161016, ENSG00000162244, ENSG00000164587, ENSG00000167658, ENSG00000168028, ENSG00000178952, ENSG00000179115, ENSG00000204628, ENSG00000229474, ENSG00000008988, ENSG00000087074, ENSG00000100316, ENSG00000105193, ENSG00000105372, ENSG00000105640, ENSG00000108107, ENSG00000108298, ENSG00000112306, ENSG00000115268, ENSG00000122140, ENSG00000130811, ENSG00000131469, ENSG00000136942, ENSG00000148303, ENSG00000149273, ENSG00000167526, ENSG00000171858, ENSG00000171863, ENSG00000177600, ENSG00000231500, ENSG00000100982, ENSG00000111640, ENSG00000099875, ENSG00000182199, ENSG00000175634, ENSG00000172531, ENSG00000136997, ENSG00000106211 |
| Up | 48 | Peptide biosynthetic process | GO:0043043 | ENSG00000081692, ENSG00000083845, ENSG00000089157, ENSG00000104529, ENSG00000114942, ENSG00000130255, ENSG00000137818, ENSG00000140988, ENSG00000142541, ENSG00000161016, ENSG00000162244, ENSG00000164587, ENSG00000167658, ENSG00000168028, ENSG00000178952, ENSG00000179115, ENSG00000204628, ENSG00000229474, ENSG00000008988, ENSG00000087074, ENSG00000100316, ENSG00000105193, ENSG00000105372, ENSG00000105640, ENSG00000108107, ENSG00000108298, ENSG00000112306, ENSG00000115268, ENSG00000122140, ENSG00000130811, ENSG00000131469, ENSG00000136942, ENSG00000148303, ENSG00000149273, ENSG00000167526, ENSG00000171858, ENSG00000171863, ENSG00000177600, ENSG00000231500, ENSG00000100982, ENSG00000111640, ENSG00000172508, ENSG00000099875, ENSG00000182199, ENSG00000175634, ENSG00000172531, ENSG00000136997, ENSG00000106211 |
| Up | 116 | Regulation of signal transduction | GO:0009966 | ENSG00000104529, ENSG00000164054, ENSG00000196924, ENSG00000056558, ENSG00000100060, ENSG00000103148, ENSG00000104880, ENSG00000104964, ENSG00000107816, ENSG00000126391, ENSG00000131634, ENSG00000149273, ENSG00000158050, ENSG00000164086, ENSG00000167984, ENSG00000179526, ENSG00000186174, ENSG00000198055, ENSG00000198286, ENSG00000198873, ENSG00000274070, ENSG00000164867, ENSG00000172531, ENSG00000008988, ENSG00000050327, ENSG00000103495, ENSG00000104814, ENSG00000106290, ENSG00000107175, ENSG00000109062, ENSG00000110651, ENSG00000111640, ENSG00000115268, ENSG00000131653, ENSG00000136997, ENSG00000140682, ENSG00000141564, ENSG00000160959, ENSG00000162676, ENSG00000167114, ENSG00000167895, ENSG00000168067, ENSG00000168646, ENSG00000168758, ENSG00000173511, ENSG00000183207, ENSG00000184371, ENSG00000185043, ENSG00000204628, ENSG00000037280, ENSG00000100324, ENSG00000104825, ENSG00000105373, ENSG00000105701, ENSG00000106211, ENSG00000107485, ENSG00000110711, ENSG00000112033, ENSG00000114737, ENSG00000115085, ENSG00000123159, ENSG00000125637, ENSG00000126456, ENSG00000135439, ENSG00000135916, ENSG00000137265, ENSG00000137497, ENSG00000139193, ENSG00000144476, ENSG00000158856, ENSG00000160014, ENSG00000163346, ENSG00000166289, ENSG00000167815, ENSG00000168286, ENSG00000175463, ENSG00000184381, ENSG00000185187, ENSG00000185989, ENSG00000188191, ENSG00000197283, ENSG00000197457, ENSG00000205336, ENSG00000213626, ENSG00000275342, ENSG00000112306, ENSG00000135144, ENSG00000171608, ENSG00000054148, ENSG00000061273, ENSG00000064545, ENSG00000070423, ENSG00000100304, ENSG00000101445, ENSG00000101997, ENSG00000103266, ENSG00000104872, ENSG00000105122, ENSG00000116670, ENSG00000120885, ENSG00000126457, ENSG00000136878, ENSG00000141522, ENSG00000161999, ENSG00000167965, ENSG00000171863, ENSG00000176108, ENSG00000182866, ENSG00000186716, ENSG00000074416, ENSG00000131196, ENSG00000014216, ENSG00000087074, ENSG00000090006, ENSG00000139725, ENSG00000173846 |
| Up | 51 | Amide biosynthetic process | GO:0043604 | ENSG00000081692, ENSG00000083845, ENSG00000089157, ENSG00000104529, ENSG00000114942, ENSG00000130255, ENSG00000137818, ENSG00000140988, ENSG00000142541, ENSG00000161016, ENSG00000162244, ENSG00000164587, ENSG00000167658, ENSG00000168028, ENSG00000172057, ENSG00000178952, ENSG00000179115, ENSG00000184381, ENSG00000204628, ENSG00000229474, ENSG00000008988, ENSG00000087074, ENSG00000100316, ENSG00000105193, ENSG00000105372, ENSG00000105640, ENSG00000108107, ENSG00000108298, ENSG00000112306, ENSG00000115268, ENSG00000122140, ENSG00000130811, ENSG00000131469, ENSG00000136942, ENSG00000148303, ENSG00000149273, ENSG00000167526, ENSG00000171858, ENSG00000171863, ENSG00000177600, ENSG00000231500, ENSG00000100982, ENSG00000111640, ENSG00000172508, ENSG00000099875, ENSG00000182199, ENSG00000175634, ENSG00000172531, ENSG00000136997, ENSG00000100075, ENSG00000106211 |
| Up | 52 | Peptide metabolic process | GO:0006518 | ENSG00000081692, ENSG00000083845, ENSG00000089157, ENSG00000104529, ENSG00000114942, ENSG00000130255, ENSG00000137818, ENSG00000140988, ENSG00000142541, ENSG00000161016, ENSG00000162244, ENSG00000164587, ENSG00000167658, ENSG00000168028, ENSG00000178952, ENSG00000179115, ENSG00000204628, ENSG00000229474, ENSG00000008988, ENSG00000087074, ENSG00000100316, ENSG00000105193, ENSG00000105372, ENSG00000105640, ENSG00000108107, ENSG00000108298, ENSG00000112306, ENSG00000115268, ENSG00000122140, ENSG00000130811, ENSG00000131469, ENSG00000136942, ENSG00000148303, ENSG00000149273, ENSG00000167526, ENSG00000171858, ENSG00000171863, ENSG00000177600, ENSG00000231500, ENSG00000100982, ENSG00000111640, ENSG00000120885, ENSG00000172508, ENSG00000099875, ENSG00000136717, ENSG00000148334, ENSG00000182199, ENSG00000107331, ENSG00000175634, ENSG00000172531, ENSG00000136997, ENSG00000106211 |
| Up | 125 | Regulation of signaling | GO:0023051 | ENSG00000104529, ENSG00000164054, ENSG00000196924, ENSG00000056558, ENSG00000100060, ENSG00000103148, ENSG00000104880, ENSG00000104964, ENSG00000107816, ENSG00000126391, ENSG00000131634, ENSG00000149273, ENSG00000158050, ENSG00000164086, ENSG00000167984, ENSG00000179526, ENSG00000186174, ENSG00000198055, ENSG00000198286, ENSG00000198873, ENSG00000274070, ENSG00000164867, ENSG00000172531, ENSG00000008988, ENSG00000050327, ENSG00000103495, ENSG00000104814, ENSG00000106290, ENSG00000107175, ENSG00000109062, ENSG00000110651, ENSG00000111640, ENSG00000115268, ENSG00000131653, ENSG00000135631, ENSG00000136997, ENSG00000140682, ENSG00000141564, ENSG00000160959, ENSG00000162676, ENSG00000167114, ENSG00000167895, ENSG00000168067, ENSG00000168646, ENSG00000168758, ENSG00000173511, ENSG00000183207, ENSG00000184371, ENSG00000185043, ENSG00000204628, ENSG00000037280, ENSG00000096433, ENSG00000100324, ENSG00000104825, ENSG00000105373, ENSG00000105701, ENSG00000106211, ENSG00000107485, ENSG00000110711, ENSG00000112033, ENSG00000113758, ENSG00000114737, ENSG00000115085, ENSG00000119714, ENSG00000123159, ENSG00000125637, ENSG00000126456, ENSG00000135439, ENSG00000135916, ENSG00000136802, ENSG00000137265, ENSG00000137497, ENSG00000139193, ENSG00000144476, ENSG00000154146, ENSG00000158856, ENSG00000160014, ENSG00000163346, ENSG00000166289, ENSG00000167815, ENSG00000168286, ENSG00000175463, ENSG00000184381, ENSG00000185187, ENSG00000185989, ENSG00000186716, ENSG00000188191, ENSG00000197283, ENSG00000197457, ENSG00000205336, ENSG00000213626, ENSG00000275342, ENSG00000112306, ENSG00000135144, ENSG00000171608, ENSG00000054148, ENSG00000061273, ENSG00000064545, ENSG00000070423, ENSG00000100304, ENSG00000101445, ENSG00000101997, ENSG00000103266, ENSG00000104872, ENSG00000105122, ENSG00000116670, ENSG00000120885, ENSG00000124181, ENSG00000126457, ENSG00000136878, ENSG00000141522, ENSG00000161999, ENSG00000167965, ENSG00000171863, ENSG00000176108, ENSG00000182866, ENSG00000074416, ENSG00000131196, ENSG00000143761, ENSG00000014216, ENSG00000074370, ENSG00000087074, ENSG00000090006, ENSG00000139725, ENSG00000173846 |
| Up | 124 | Regulation of cell communication | GO:0010646 | ENSG00000104529, ENSG00000164054, ENSG00000196924, ENSG00000056558, ENSG00000100060, ENSG00000103148, ENSG00000104880, ENSG00000104964, ENSG00000107816, ENSG00000126391, ENSG00000131634, ENSG00000149273, ENSG00000158050, ENSG00000164086, ENSG00000167984, ENSG00000179526, ENSG00000186174, ENSG00000198055, ENSG00000198286, ENSG00000198873, ENSG00000274070, ENSG00000160014, ENSG00000164867, ENSG00000172531, ENSG00000008988, ENSG00000050327, ENSG00000103495, ENSG00000104814, ENSG00000106290, ENSG00000107175, ENSG00000109062, ENSG00000110651, ENSG00000111640, ENSG00000115268, ENSG00000131653, ENSG00000135631, ENSG00000136997, ENSG00000140682, ENSG00000141564, ENSG00000160959, ENSG00000162676, ENSG00000167114, ENSG00000167895, ENSG00000168067, ENSG00000168646, ENSG00000168758, ENSG00000173511, ENSG00000183207, ENSG00000184371, ENSG00000185043, ENSG00000204628, ENSG00000037280, ENSG00000096433, ENSG00000100324, ENSG00000104825, ENSG00000105373, ENSG00000105701, ENSG00000106211, ENSG00000107485, ENSG00000110711, ENSG00000112033, ENSG00000113758, ENSG00000114737, ENSG00000115085, ENSG00000119714, ENSG00000123159, ENSG00000125637, ENSG00000126456, ENSG00000135439, ENSG00000135916, ENSG00000136802, ENSG00000137265, ENSG00000137497, ENSG00000139193, ENSG00000144476, ENSG00000154146, ENSG00000158856, ENSG00000163346, ENSG00000166289, ENSG00000167815, ENSG00000168286, ENSG00000175463, ENSG00000184381, ENSG00000185187, ENSG00000185989, ENSG00000186716, ENSG00000188191, ENSG00000197283, ENSG00000197457, ENSG00000205336, ENSG00000213626, ENSG00000275342, ENSG00000112306, ENSG00000135144, ENSG00000171608, ENSG00000054148, ENSG00000061273, ENSG00000064545, ENSG00000070423, ENSG00000100304, ENSG00000101445, ENSG00000101997, ENSG00000103266, ENSG00000104872, ENSG00000105122, ENSG00000116670, ENSG00000120885, ENSG00000124181, ENSG00000126457, ENSG00000136878, ENSG00000141522, ENSG00000161999, ENSG00000167965, ENSG00000171863, ENSG00000176108, ENSG00000182866, ENSG00000074416, ENSG00000131196, ENSG00000143761, ENSG00000014216, ENSG00000087074, ENSG00000090006, ENSG00000139725, ENSG00000173846 |
| Up | 35 | Histone modification | GO:0016570 | ENSG00000061273, ENSG00000081692, ENSG00000099381, ENSG00000101945, ENSG00000126457, ENSG00000130382, ENSG00000167770, ENSG00000170468, ENSG00000183207, ENSG00000198026, ENSG00000075624, ENSG00000132382, ENSG00000169976, ENSG00000172534, ENSG00000043143, ENSG00000071655, ENSG00000106290, ENSG00000111752, ENSG00000136807, ENSG00000167491, ENSG00000057657, ENSG00000105329, ENSG00000107485, ENSG00000112511, ENSG00000124575, ENSG00000137265, ENSG00000162676, ENSG00000163346, ENSG00000168298, ENSG00000187837, ENSG00000275023, ENSG00000055483, ENSG00000104872, ENSG00000149923, ENSG00000166313 |
| Up | 132 | Protein modification process | GO:0036211 | ENSG00000054148, ENSG00000055483, ENSG00000056558, ENSG00000061273, ENSG00000071575, ENSG00000081692, ENSG00000087074, ENSG00000099381, ENSG00000099875, ENSG00000101445, ENSG00000101945, ENSG00000103266, ENSG00000105063, ENSG00000126457, ENSG00000129968, ENSG00000130382, ENSG00000130726, ENSG00000135144, ENSG00000136807, ENSG00000141552, ENSG00000141564, ENSG00000143851, ENSG00000149923, ENSG00000161013, ENSG00000167770, ENSG00000170468, ENSG00000173456, ENSG00000173511, ENSG00000173540, ENSG00000175634, ENSG00000178498, ENSG00000179526, ENSG00000182208, ENSG00000182858, ENSG00000183207, ENSG00000185189, ENSG00000188191, ENSG00000188322, ENSG00000197858, ENSG00000198026, ENSG00000198055, ENSG00000198873, ENSG00000204628, ENSG00000243477, ENSG00000247077, ENSG00000275342, ENSG00000075624, ENSG00000132382, ENSG00000169976, ENSG00000172534, ENSG00000020633, ENSG00000037280, ENSG00000043143, ENSG00000050327, ENSG00000062524, ENSG00000070423, ENSG00000071655, ENSG00000079999, ENSG00000100324, ENSG00000101224, ENSG00000102226, ENSG00000104814, ENSG00000106290, ENSG00000106683, ENSG00000110651, ENSG00000111752, ENSG00000115085, ENSG00000116670, ENSG00000131653, ENSG00000135439, ENSG00000136878, ENSG00000140988, ENSG00000149273, ENSG00000160014, ENSG00000164086, ENSG00000164896, ENSG00000167491, ENSG00000168067, ENSG00000170604, ENSG00000171863, ENSG00000182866, ENSG00000204209, ENSG00000007264, ENSG00000008988, ENSG00000057657, ENSG00000100304, ENSG00000105329, ENSG00000106211, ENSG00000107140, ENSG00000107485, ENSG00000107742, ENSG00000109062, ENSG00000112511, ENSG00000114737, ENSG00000115268, ENSG00000119669, ENSG00000124575, ENSG00000130311, ENSG00000137265, ENSG00000137818, ENSG00000142546, ENSG00000149541, ENSG00000158050, ENSG00000158856, ENSG00000162676, ENSG00000163346, ENSG00000167965, ENSG00000168298, ENSG00000168646, ENSG00000171823, ENSG00000172531, ENSG00000173846, ENSG00000184371, ENSG00000184381, ENSG00000185043, ENSG00000186716, ENSG00000187837, ENSG00000197562, ENSG00000275023, ENSG00000104872, ENSG00000105373, ENSG00000105701, ENSG00000107331, ENSG00000120885, ENSG00000176108, ENSG00000182199, ENSG00000196924, ENSG00000111640, ENSG00000166313, ENSG00000171608, ENSG00000079435, ENSG00000102178 |
| Up | 141 | Regulation of response to stimulus | GO:0048583 | ENSG00000104529, ENSG00000164054, ENSG00000196924, ENSG00000056558, ENSG00000077984, ENSG00000100060, ENSG00000103148, ENSG00000104880, ENSG00000104964, ENSG00000107816, ENSG00000112033, ENSG00000126391, ENSG00000131634, ENSG00000149273, ENSG00000158050, ENSG00000164086, ENSG00000167770, ENSG00000167984, ENSG00000168758, ENSG00000173511, ENSG00000179526, ENSG00000182866, ENSG00000186174, ENSG00000198055, ENSG00000198286, ENSG00000198873, ENSG00000213654, ENSG00000274070, ENSG00000075624, ENSG00000106290, ENSG00000116670, ENSG00000164867, ENSG00000169976, ENSG00000172531, ENSG00000183207, ENSG00000008988, ENSG00000050327, ENSG00000096996, ENSG00000103495, ENSG00000104814, ENSG00000105372, ENSG00000107175, ENSG00000109062, ENSG00000110651, ENSG00000111640, ENSG00000115268, ENSG00000124181, ENSG00000126456, ENSG00000130726, ENSG00000131653, ENSG00000136807, ENSG00000136997, ENSG00000140682, ENSG00000141564, ENSG00000153563, ENSG00000160959, ENSG00000162676, ENSG00000167114, ENSG00000167895, ENSG00000168067, ENSG00000168646, ENSG00000173456, ENSG00000176083, ENSG00000184371, ENSG00000185043, ENSG00000186810, ENSG00000204628, ENSG00000037280, ENSG00000073861, ENSG00000089692, ENSG00000100324, ENSG00000104825, ENSG00000105329, ENSG00000105373, ENSG00000105701, ENSG00000106211, ENSG00000107485, ENSG00000110711, ENSG00000114737, ENSG00000115085, ENSG00000119242, ENSG00000120885, ENSG00000123159, ENSG00000125637, ENSG00000128340, ENSG00000130827, ENSG00000135439, ENSG00000135916, ENSG00000137265, ENSG00000137497, ENSG00000139193, ENSG00000144476, ENSG00000158856, ENSG00000159840, ENSG00000160014, ENSG00000163346, ENSG00000166289, ENSG00000167815, ENSG00000168286, ENSG00000175463, ENSG00000184381, ENSG00000185187, ENSG00000185989, ENSG00000186716, ENSG00000188191, ENSG00000197283, ENSG00000197457, ENSG00000205336, ENSG00000213626, ENSG00000275342, ENSG00000112306, ENSG00000135144, ENSG00000171608, ENSG00000054148, ENSG00000061273, ENSG00000064545, ENSG00000070423, ENSG00000100304, ENSG00000101445, ENSG00000101997, ENSG00000103266, ENSG00000104872, ENSG00000105122, ENSG00000126457, ENSG00000136878, ENSG00000141522, ENSG00000149923, ENSG00000161999, ENSG00000167965, ENSG00000171863, ENSG00000176108, ENSG00000184009, ENSG00000074416, ENSG00000105374, ENSG00000131196, ENSG00000014216, ENSG00000087074, ENSG00000090006, ENSG00000106683, ENSG00000139725, ENSG00000173846 |
| Up | 130 | Cellular protein modification process | GO:0006464 | ENSG00000054148, ENSG00000055483, ENSG00000056558, ENSG00000061273, ENSG00000071575, ENSG00000081692, ENSG00000087074, ENSG00000099381, ENSG00000099875, ENSG00000101445, ENSG00000101945, ENSG00000103266, ENSG00000105063, ENSG00000126457, ENSG00000129968, ENSG00000130382, ENSG00000130726, ENSG00000135144, ENSG00000136807, ENSG00000141552, ENSG00000141564, ENSG00000143851, ENSG00000149923, ENSG00000161013, ENSG00000167770, ENSG00000170468, ENSG00000173456, ENSG00000173511, ENSG00000173540, ENSG00000175634, ENSG00000178498, ENSG00000179526, ENSG00000182208, ENSG00000182858, ENSG00000183207, ENSG00000185189, ENSG00000188191, ENSG00000188322, ENSG00000197858, ENSG00000198026, ENSG00000198055, ENSG00000198873, ENSG00000204628, ENSG00000243477, ENSG00000247077, ENSG00000275342, ENSG00000075624, ENSG00000132382, ENSG00000169976, ENSG00000172534, ENSG00000020633, ENSG00000037280, ENSG00000043143, ENSG00000050327, ENSG00000062524, ENSG00000070423, ENSG00000071655, ENSG00000079999, ENSG00000100324, ENSG00000101224, ENSG00000102226, ENSG00000104814, ENSG00000106290, ENSG00000106683, ENSG00000110651, ENSG00000111752, ENSG00000115085, ENSG00000116670, ENSG00000131653, ENSG00000135439, ENSG00000136878, ENSG00000140988, ENSG00000149273, ENSG00000160014, ENSG00000164086, ENSG00000164896, ENSG00000167491, ENSG00000168067, ENSG00000170604, ENSG00000171863, ENSG00000182866, ENSG00000204209, ENSG00000007264, ENSG00000008988, ENSG00000057657, ENSG00000105329, ENSG00000106211, ENSG00000107140, ENSG00000107485, ENSG00000107742, ENSG00000109062, ENSG00000112511, ENSG00000114737, ENSG00000115268, ENSG00000119669, ENSG00000124575, ENSG00000130311, ENSG00000137265, ENSG00000137818, ENSG00000142546, ENSG00000149541, ENSG00000158050, ENSG00000158856, ENSG00000162676, ENSG00000163346, ENSG00000167965, ENSG00000168298, ENSG00000168646, ENSG00000171823, ENSG00000172531, ENSG00000173846, ENSG00000184371, ENSG00000184381, ENSG00000185043, ENSG00000186716, ENSG00000187837, ENSG00000197562, ENSG00000275023, ENSG00000104872, ENSG00000105373, ENSG00000105701, ENSG00000107331, ENSG00000120885, ENSG00000176108, ENSG00000182199, ENSG00000196924, ENSG00000111640, ENSG00000166313, ENSG00000171608, ENSG00000079435 |
| Up | 115 | Regulation of molecular function | GO:0065009 | ENSG00000037280, ENSG00000050327, ENSG00000056558, ENSG00000062524, ENSG00000068831, ENSG00000071575, ENSG00000077984, ENSG00000079999, ENSG00000087074, ENSG00000102977, ENSG00000104825, ENSG00000105063, ENSG00000108961, ENSG00000130827, ENSG00000141564, ENSG00000142459, ENSG00000160014, ENSG00000160959, ENSG00000163346, ENSG00000164867, ENSG00000175463, ENSG00000188191, ENSG00000008517, ENSG00000011132, ENSG00000014216, ENSG00000054148, ENSG00000071564, ENSG00000074370, ENSG00000100324, ENSG00000104964, ENSG00000106683, ENSG00000107175, ENSG00000107331, ENSG00000110651, ENSG00000111640, ENSG00000116670, ENSG00000135439, ENSG00000136807, ENSG00000136997, ENSG00000140988, ENSG00000142546, ENSG00000149273, ENSG00000149541, ENSG00000162676, ENSG00000164086, ENSG00000167114, ENSG00000167895, ENSG00000167984, ENSG00000168067, ENSG00000171863, ENSG00000182866, ENSG00000185101, ENSG00000186716, ENSG00000196924, ENSG00000198286, ENSG00000204628, ENSG00000276045, ENSG00000008988, ENSG00000072818, ENSG00000075624, ENSG00000099991, ENSG00000100060, ENSG00000101224, ENSG00000101445, ENSG00000103148, ENSG00000103266, ENSG00000104529, ENSG00000104880, ENSG00000104884, ENSG00000105122, ENSG00000105329, ENSG00000106211, ENSG00000106351, ENSG00000107140, ENSG00000107485, ENSG00000109062, ENSG00000110711, ENSG00000114737, ENSG00000114942, ENSG00000115268, ENSG00000125637, ENSG00000125651, ENSG00000128340, ENSG00000130382, ENSG00000130726, ENSG00000136717, ENSG00000137265, ENSG00000137818, ENSG00000139193, ENSG00000139725, ENSG00000141522, ENSG00000167815, ENSG00000167965, ENSG00000168286, ENSG00000168646, ENSG00000172057, ENSG00000179588, ENSG00000182208, ENSG00000184371, ENSG00000184381, ENSG00000185043, ENSG00000185989, ENSG00000197283, ENSG00000197457, ENSG00000204209, ENSG00000213654, ENSG00000247077, ENSG00000101997, ENSG00000104872, ENSG00000120885, ENSG00000161999, ENSG00000176108, ENSG00000185187, ENSG00000172531, ENSG00000107742 |
| Up | 136 | Macromolecule modification | GO:0043412 | ENSG00000054148, ENSG00000055483, ENSG00000056558, ENSG00000061273, ENSG00000071575, ENSG00000081692, ENSG00000087074, ENSG00000099381, ENSG00000099875, ENSG00000101445, ENSG00000101945, ENSG00000103266, ENSG00000105063, ENSG00000126457, ENSG00000129968, ENSG00000130382, ENSG00000130726, ENSG00000135144, ENSG00000136807, ENSG00000141552, ENSG00000141564, ENSG00000143851, ENSG00000149923, ENSG00000161013, ENSG00000167770, ENSG00000170468, ENSG00000173456, ENSG00000173511, ENSG00000173540, ENSG00000175634, ENSG00000178252, ENSG00000178498, ENSG00000179526, ENSG00000182208, ENSG00000182858, ENSG00000183207, ENSG00000185189, ENSG00000188191, ENSG00000188322, ENSG00000197858, ENSG00000198026, ENSG00000198055, ENSG00000198873, ENSG00000204628, ENSG00000213339, ENSG00000243477, ENSG00000247077, ENSG00000275342, ENSG00000075624, ENSG00000132382, ENSG00000169976, ENSG00000172534, ENSG00000020633, ENSG00000037280, ENSG00000043143, ENSG00000050327, ENSG00000062524, ENSG00000070423, ENSG00000071655, ENSG00000079999, ENSG00000100324, ENSG00000100982, ENSG00000101224, ENSG00000102226, ENSG00000104814, ENSG00000106290, ENSG00000106683, ENSG00000110651, ENSG00000111752, ENSG00000115085, ENSG00000116670, ENSG00000131653, ENSG00000135439, ENSG00000136878, ENSG00000140988, ENSG00000149273, ENSG00000160014, ENSG00000164086, ENSG00000164896, ENSG00000167491, ENSG00000168067, ENSG00000170604, ENSG00000171863, ENSG00000182866, ENSG00000204209, ENSG00000007264, ENSG00000008988, ENSG00000057657, ENSG00000100304, ENSG00000105329, ENSG00000106211, ENSG00000107140, ENSG00000107485, ENSG00000107742, ENSG00000109062, ENSG00000112511, ENSG00000114737, ENSG00000115268, ENSG00000119669, ENSG00000124575, ENSG00000130311, ENSG00000137265, ENSG00000137818, ENSG00000142546, ENSG00000149541, ENSG00000158050, ENSG00000158856, ENSG00000162676, ENSG00000163346, ENSG00000167965, ENSG00000168298, ENSG00000168646, ENSG00000171823, ENSG00000172531, ENSG00000173846, ENSG00000184371, ENSG00000184381, ENSG00000185043, ENSG00000186716, ENSG00000187837, ENSG00000197562, ENSG00000275023, ENSG00000104872, ENSG00000105373, ENSG00000105701, ENSG00000107331, ENSG00000120885, ENSG00000136997, ENSG00000176108, ENSG00000182199, ENSG00000196924, ENSG00000111640, ENSG00000166313, ENSG00000171608, ENSG00000079435, ENSG00000102178 |
| Down | 9 | Regulation of response to biotic stimulus | GO:0002831 | ENSG00000138646, ENSG00000154589, ENSG00000101916, ENSG00000090376, ENSG00000124882, ENSG00000164136, ENSG00000185745, ENSG00000107201, ENSG00000256660 |
| Down | 9 | Response to virus | GO:0009615 | ENSG00000101916, ENSG00000107201, ENSG00000119922, ENSG00000138646, ENSG00000185745, ENSG00000090376, ENSG00000137959, ENSG00000164136, ENSG00000137965 |
| Down | 14 | Defense response to other organism | GO:0098542 | ENSG00000101916, ENSG00000107201, ENSG00000119922, ENSG00000138646, ENSG00000166527, ENSG00000185745, ENSG00000154589, ENSG00000115919, ENSG00000137959, ENSG00000090376, ENSG00000124882, ENSG00000164136, ENSG00000179630, ENSG00000256660 |
| Down | 17 | Immune response | GO:0006955 | ENSG00000101916, ENSG00000107201, ENSG00000137959, ENSG00000137965, ENSG00000154589, ENSG00000164136, ENSG00000166527, ENSG00000175857, ENSG00000256660, ENSG00000147894, ENSG00000115919, ENSG00000179630, ENSG00000185745, ENSG00000090376, ENSG00000119922, ENSG00000124882, ENSG00000138646 |
| Down | 15 | Response to biotic stimulus | GO:0009607 | ENSG00000101916, ENSG00000107201, ENSG00000119922, ENSG00000138646, ENSG00000154589, ENSG00000166527, ENSG00000185745, ENSG00000090376, ENSG00000115919, ENSG00000137959, ENSG00000124882, ENSG00000137965, ENSG00000164136, ENSG00000179630, ENSG00000256660 |
| Down | 15 | Response to external biotic stimulus | GO:0043207 | ENSG00000101916, ENSG00000107201, ENSG00000119922, ENSG00000138646, ENSG00000154589, ENSG00000166527, ENSG00000185745, ENSG00000090376, ENSG00000115919, ENSG00000137959, ENSG00000124882, ENSG00000137965, ENSG00000164136, ENSG00000179630, ENSG00000256660 |
| Down | 12 | Innate immune response | GO:0045087 | ENSG00000107201, ENSG00000166527, ENSG00000154589, ENSG00000101916, ENSG00000115919, ENSG00000185745, ENSG00000090376, ENSG00000119922, ENSG00000124882, ENSG00000138646, ENSG00000179630, ENSG00000256660 |
| Down | 7 | Defense response to virus | GO:0051607 | ENSG00000101916, ENSG00000119922, ENSG00000138646, ENSG00000185745, ENSG00000107201, ENSG00000137959, ENSG00000164136 |
| Down | 15 | Response to other organism | GO:0051707 | ENSG00000101916, ENSG00000107201, ENSG00000119922, ENSG00000138646, ENSG00000154589, ENSG00000166527, ENSG00000185745, ENSG00000090376, ENSG00000115919, ENSG00000137959, ENSG00000124882, ENSG00000137965, ENSG00000164136, ENSG00000179630, ENSG00000256660 |
| Down | 7 | Defense response to symbiont | GO:0140546 | ENSG00000101916, ENSG00000119922, ENSG00000138646, ENSG00000185745, ENSG00000107201, ENSG00000137959, ENSG00000164136 |
| Down | 15 | Biological process involved in interspecies interaction between organisms | GO:0044419 | ENSG00000101916, ENSG00000107201, ENSG00000119922, ENSG00000138646, ENSG00000154589, ENSG00000166527, ENSG00000185745, ENSG00000090376, ENSG00000115919, ENSG00000137959, ENSG00000124882, ENSG00000137965, ENSG00000164136, ENSG00000179630, ENSG00000256660 |
| Down | 4 | Regulation of defense response to virus | GO:0050688 | ENSG00000138646, ENSG00000164136, ENSG00000185745, ENSG00000107201 |
| Down | 9 | Regulation of defense response | GO:0031347 | ENSG00000138646, ENSG00000164136, ENSG00000101916, ENSG00000090376, ENSG00000124882, ENSG00000179630, ENSG00000185745, ENSG00000107201, ENSG00000256660 |
| Down | 5 | Pattern recognition receptor signaling pathway | GO:0002221 | ENSG00000101916, ENSG00000154589, ENSG00000107201, ENSG00000179630, ENSG00000090376 |
| Down | 3 | Positive regulation of Notch signaling pathway | GO:0045747 | ENSG00000264343, ENSG00000286019, ENSG00000286219 |

**Table S7:** Spearman correlation coefficients (rho) and 2-tailed p-values (p) for dysregulated lncRNAs (normalized counts) at 36-38 weeks (N=6 normal pregnant controls, N=4 preeclampsia) correlated to selected clinical markers. Statistically significant correlations are marked in bold.

| **Ensembl** | **Symbol** |  | **PlGF** | **AOPP** | **sFlt1** | **bgal** | **BAFF** | **CD 163** | **CD14** | **sCD 25** | **VWF** | **psel** | **tm** | **esel** | **MPO** |
| --- | --- | --- | --- | --- | --- | --- | --- | --- | --- | --- | --- | --- | --- | --- | --- |
| ENSG00000174171 | LOC105370792 | rho | -0.14 | 0.01 | 0.50 | 0.20 | 0.30 | 0.14 | 0.07 | 0.20 | -0.20 | -0.46 | -0.49 | -0.26 | 0.58 |
|  |  | p | 0.701 | 0.987 | 0.138 | 0.58 | 0.405 | 0.701 | 0.855 | 0.58 | 0.58 | 0.187 | 0.15 | 0.467 | 0.08 |
| ENSG00000287823 | ENSG00000287823 | rho | -0.12 | -0.13 | 0.48 | 0.04 | 0.44 | 0.26 | 0.27 | -0.15 | -0.37 | -0.55 | -0.31 | -0.21 | 0.23 |
|  |  | p | 0.751 | 0.726 | 0.162 | 0.907 | 0.2 | 0.467 | 0.446 | 0.676 | 0.293 | 0.098 | 0.385 | 0.556 | 0.521 |
| ENSG00000254995 | STX16-NPEPL1 | rho | -0.35 | -0.15 | 0.50 | 0.37 | 0.41 | 0.29 | 0.32 | 0.14 | -0.44 | -0.55 | -0.49 | -0.06 | 0.51 |
|  |  | p | 0.328 | 0.676 | 0.138 | 0.293 | 0.244 | 0.425 | 0.365 | 0.701 | 0.2 | 0.098 | 0.15 | 0.881 | 0.137 |
| ENSG00000232487 | RASA3-IT1 | rho | -0.35 | -0.19 | 0.52 | 0.35 | 0.24 | 0.15 | 0.32 | 0.18 | -0.33 | -0.61 | **-0.64** | -0.21 | **0.67** |
|  |  | p | 0.328 | 0.603 | 0.128 | 0.328 | 0.511 | 0.676 | 0.365 | 0.627 | 0.347 | 0.06 | **0.048** | 0.556 | **0.035** |
| ENSG00000238164 | TNFRSF14-AS1 | rho | -0.41 | -0.13 | 0.55 | 0.33 | 0.53 | 0.43 | 0.39 | 0.22 | -0.39 | -0.47 | -0.37 | 0.08 | 0.45 |
|  |  | p | 0.244 | 0.726 | 0.098 | 0.347 | 0.117 | 0.214 | 0.26 | 0.533 | 0.26 | 0.174 | 0.293 | 0.829 | 0.192 |
| ENSG00000237181 | PRKAR1B-AS1 | rho | -0.38 | -0.27 | 0.53 | 0.32 | 0.39 | 0.24 | 0.52 | 0.03 | -0.44 | **-0.70** | -0.56 | -0.14 | 0.47 |
|  |  | p | 0.276 | 0.446 | 0.117 | 0.365 | 0.26 | 0.511 | 0.128 | 0.934 | 0.2 | **0.025** | 0.09 | 0.701 | 0.172 |
| ENSG00000277476 | ENSG00000277476 | rho | -0.18 | -0.25 | 0.46 | 0.10 | 0.41 | 0.29 | 0.27 | 0.16 | -0.37 | -0.50 | -0.44 | -0.13 | 0.42 |
|  |  | p | 0.627 | 0.489 | 0.187 | 0.777 | 0.244 | 0.425 | 0.446 | 0.651 | 0.293 | 0.138 | 0.2 | 0.726 | 0.228 |
| ENSG00000289398 | ENSG00000289398 | rho | -0.07 | -0.06 | 0.39 | 0.09 | 0.18 | -0.07 | 0.06 | -0.04 | -0.31 | -0.61 | -0.48 | -0.43 | 0.46 |
|  |  | p | 0.855 | 0.881 | 0.26 | 0.803 | 0.627 | 0.855 | 0.881 | 0.907 | 0.385 | 0.06 | 0.162 | 0.214 | 0.185 |
| ENSG00000227039 | ITGB2-AS1 | rho | -0.33 | -0.30 | 0.55 | 0.08 | 0.33 | 0.42 | 0.46 | 0.06 | -0.21 | -0.46 | -0.41 | -0.08 | 0.44 |
|  |  | p | 0.347 | 0.405 | 0.098 | 0.829 | 0.347 | 0.229 | 0.187 | 0.881 | 0.556 | 0.187 | 0.244 | 0.829 | 0.199 |
| ENSG00000232442 | MHENCR | rho | -0.10 | -0.25 | 0.39 | 0.18 | 0.35 | 0.19 | 0.35 | 0.15 | -0.39 | -0.56 | -0.53 | -0.20 | 0.44 |
|  |  | p | 0.777 | 0.489 | 0.26 | 0.627 | 0.328 | 0.603 | 0.328 | 0.676 | 0.26 | 0.09 | 0.117 | 0.58 | 0.206 |
| ENSG00000290021 | antisense BCL6-AS1 | rho | -0.41 | -0.25 | 0.52 | 0.30 | 0.31 | 0.35 | 0.53 | -0.02 | -0.43 | -0.60 | -0.46 | -0.08 | 0.49 |
|  |  | p | 0.244 | 0.489 | 0.128 | 0.405 | 0.385 | 0.328 | 0.117 | 0.96 | 0.214 | 0.067 | 0.187 | 0.829 | 0.154 |
| ENSG00000290600 | ENSG00000290600 | rho | 0.12 | -0.43 | 0.19 | -0.09 | 0.10 | 0.09 | 0.30 | -0.02 | -0.43 | -0.54 | -0.48 | -0.33 | 0.33 |
|  |  | p | 0.751 | 0.214 | 0.603 | 0.803 | 0.777 | 0.803 | 0.405 | 0.96 | 0.214 | 0.108 | 0.162 | 0.347 | 0.354 |
| ENSG00000289310 | senseintronicLRRK2 | rho | 0.47 | -0.36 | **-0.72** | -0.54 | -0.50 | -0.22 | -0.16 | -0.18 | 0.24 | 0.53 | 0.37 | 0.12 | **-0.63** |
|  |  | p | 0.174 | 0.31 | **0.019** | 0.108 | 0.138 | 0.533 | 0.651 | 0.627 | 0.511 | 0.117 | 0.293 | 0.751 | **0.05** |
| ENSG00000230789 | ARHGAP26-IT1 | rho | 0.31 | -0.20 | -0.61 | -0.37 | -0.59 | -0.29 | -0.18 | -0.27 | 0.30 | 0.47 | 0.32 | 0.06 | -0.49 |
|  |  | p | 0.385 | 0.58 | 0.06 | 0.293 | 0.074 | 0.425 | 0.627 | 0.446 | 0.405 | 0.174 | 0.365 | 0.881 | 0.148 |
| ENSG00000272807 | antisense KANSL1L | rho | 0.20 | 0.02 | -0.38 | -0.31 | **-0.70** | -0.50 | -0.35 | -0.37 | 0.52 | 0.31 | 0.14 | -0.25 | -0.23 |
|  |  | p | 0.58 | 0.96 | 0.276 | 0.385 | **0.025** | 0.138 | 0.328 | 0.293 | 0.128 | 0.385 | 0.701 | 0.489 | 0.521 |
| ENSG00000232542 | DPYD-IT1 | rho | 0.32 | -0.15 | -0.50 | -0.49 | -0.60 | -0.08 | -0.04 | -0.22 | 0.43 | 0.55 | 0.42 | 0.06 | -0.39 |
|  |  | p | 0.365 | 0.676 | 0.138 | 0.15 | 0.067 | 0.829 | 0.907 | 0.533 | 0.214 | 0.098 | 0.229 | 0.881 | 0.266 |
| ENSG00000258476 | LINC02207 | rho | **0.64** | -0.22 | **-0.70** | -0.44 | -0.04 | -0.20 | -0.10 | -0.18 | 0.12 | 0.46 | 0.42 | 0.19 | **-0.95** |
|  |  | p | **0.048** | 0.533 | **0.025** | 0.2 | 0.907 | 0.58 | 0.777 | 0.627 | 0.751 | 0.187 | 0.229 | 0.603 | **<0.001** |
| ENSG00000270062 | senseintronicNIN | rho | 0.14 | 0.24 | -0.39 | -0.29 | -0.50 | -0.15 | -0.48 | 0.01 | 0.50 | **0.69** | 0.52 | 0.16 | -0.24 |
|  |  | p | 0.701 | 0.511 | 0.26 | 0.425 | 0.138 | 0.676 | 0.162 | 0.987 | 0.138 | **0.029** | 0.128 | 0.651 | 0.498 |
| ENSG00000291144 | ENSG00000291144 | rho | 0.24 | -0.04 | -0.48 | -0.25 | -0.09 | -0.10 | -0.20 | -0.25 | 0.14 | 0.46 | 0.48 | 0.29 | **-0.74** |
|  |  | p | 0.511 | 0.907 | 0.162 | 0.489 | 0.803 | 0.777 | 0.58 | 0.489 | 0.701 | 0.187 | 0.162 | 0.425 | **0.015** |
| ENSG00000277130 | antisensePPP1R12A | rho | 0.20 | 0.10 | -0.42 | -0.35 | -0.52 | -0.33 | -0.44 | -0.25 | 0.54 | 0.54 | 0.38 | 0.01 | -0.41 |
|  |  | p | 0.58 | 0.777 | 0.229 | 0.328 | 0.128 | 0.347 | 0.2 | 0.489 | 0.108 | 0.108 | 0.276 | 0.987 | 0.235 |
| ENSG00000273284 | senseintronicANKRD12 | rho | -0.07 | 0.27 | -0.26 | -0.10 | -0.54 | -0.15 | -0.38 | -0.07 | 0.39 | 0.50 | 0.41 | 0.12 | -0.05 |
|  |  | p | 0.855 | 0.446 | 0.467 | 0.777 | 0.108 | 0.676 | 0.276 | 0.855 | 0.26 | 0.138 | 0.244 | 0.751 | 0.894 |
| ENSG00000224707 | E2F3-IT1 | rho | 0.31 | -0.06 | -0.53 | -0.43 | -0.58 | -0.29 | -0.32 | -0.32 | 0.43 | 0.53 | 0.39 | 0.01 | -0.49 |
|  |  | p | 0.385 | 0.881 | 0.117 | 0.214 | 0.082 | 0.425 | 0.365 | 0.365 | 0.214 | 0.117 | 0.26 | 0.987 | 0.154 |
| ENSG00000248429 | GASK1B-AS1 | rho | 0.36 | -0.19 | -0.52 | -0.58 | -0.58 | -0.09 | -0.10 | -0.27 | 0.44 | 0.56 | 0.44 | 0.04 | -0.47 |
|  |  | p | 0.31 | 0.603 | 0.128 | 0.082 | 0.082 | 0.803 | 0.777 | 0.446 | 0.2 | 0.09 | 0.2 | 0.907 | 0.172 |
| ENSG00000250608 | NUDT16-DT | rho | 0.39 | -0.29 | **-0.66** | -0.44 | -0.42 | -0.19 | -0.18 | -0.20 | 0.25 | 0.54 | 0.38 | 0.18 | **-0.66** |
|  |  | p | 0.26 | 0.425 | **0.038** | 0.2 | 0.229 | 0.603 | 0.627 | 0.58 | 0.489 | 0.108 | 0.276 | 0.627 | **0.039** |
| ENSG00000277151 | ENSG00000277151 | rho | 0.33 | 0.18 | -0.48 | -0.37 | -0.37 | -0.31 | -0.49 | -0.22 | 0.49 | 0.60 | 0.49 | 0.07 | -0.58 |
|  |  | p | 0.347 | 0.627 | 0.162 | 0.293 | 0.293 | 0.385 | 0.15 | 0.533 | 0.15 | 0.067 | 0.15 | 0.855 | 0.08 |
| ENSG00000236709 | DAPK1-IT1 | rho | 0.36 | -0.44 | **-0.69** | -0.53 | -0.46 | -0.15 | -0.08 | -0.04 | 0.21 | 0.52 | 0.35 | 0.20 | -0.57 |
|  |  | p | 0.31 | 0.2 | **0.029** | 0.117 | 0.187 | 0.676 | 0.829 | 0.907 | 0.556 | 0.128 | 0.328 | 0.58 | 0.084 |
| ENSG00000266126 | ENSG00000266126 | rho | 0.04 | -0.06 | -0.27 | -0.44 | -0.53 | -0.39 | -0.43 | -0.25 | **0.65** | 0.41 | 0.21 | -0.10 | -0.30 |
|  |  | p | 0.907 | 0.881 | 0.446 | 0.2 | 0.117 | 0.26 | 0.214 | 0.489 | **0.043** | 0.244 | 0.556 | 0.777 | 0.393 |
| ENSG00000267632 | ENSG00000267632 | rho | 0.55 | 0.02 | -0.59 | -0.46 | -0.14 | 0.04 | -0.10 | -0.04 | 0.33 | **0.71** | **0.64** | 0.30 | **-0.75** |
|  |  | p | 0.098 | 0.96 | 0.074 | 0.187 | 0.701 | 0.907 | 0.777 | 0.907 | 0.347 | **0.022** | **0.048** | 0.405 | **0.012** |
| ENSG00000289691 | ENSG00000289691 | rho | 0.22 | -0.33 | -0.49 | -0.56 | -0.37 | -0.02 | -0.10 | -0.15 | 0.37 | 0.56 | 0.43 | 0.22 | -0.57 |
|  |  | p | 0.533 | 0.347 | 0.15 | 0.09 | 0.293 | 0.96 | 0.777 | 0.676 | 0.293 | 0.09 | 0.214 | 0.533 | 0.084 |
| ENSG00000270562 | FOXP1-DT | rho | 0.27 | -0.25 | **-0.64** | -0.43 | -0.49 | -0.18 | -0.27 | -0.10 | 0.22 | 0.56 | 0.42 | 0.21 | -0.52 |
|  |  | p | 0.446 | 0.489 | **0.048** | 0.214 | 0.15 | 0.627 | 0.446 | 0.777 | 0.533 | 0.09 | 0.229 | 0.556 | 0.126 |
| ENSG00000289470 | ENSG00000289470 | rho | 0.47 | -0.07 | **-0.72** | -0.37 | -0.38 | -0.14 | -0.08 | -0.07 | 0.12 | 0.55 | 0.56 | 0.22 | -0.62 |
|  |  | p | 0.174 | 0.855 | **0.019** | 0.293 | 0.276 | 0.701 | 0.829 | 0.855 | 0.751 | 0.098 | 0.09 | 0.533 | 0.056 |
| ENSG00000255893 | ENSG00000255893 | rho | 0.21 | 0.01 | -0.30 | -0.50 | -0.36 | -0.09 | -0.22 | -0.27 | **0.66** | 0.59 | 0.44 | 0.07 | -0.51 |
|  |  | p | 0.556 | 0.987 | 0.405 | 0.138 | 0.31 | 0.803 | 0.533 | 0.446 | **0.038** | 0.074 | 0.2 | 0.855 | 0.137 |
| ENSG00000289617 | senseintronicFRY | rho | 0.21 | -0.03 | -0.35 | -0.44 | -0.47 | 0.16 | -0.10 | -0.14 | 0.47 | **0.67** | 0.52 | 0.20 | -0.30 |
|  |  | p | 0.556 | 0.934 | 0.328 | 0.2 | 0.174 | 0.651 | 0.777 | 0.701 | 0.174 | **0.033** | 0.128 | 0.58 | 0.393 |
| ENSG00000288899 | ENSG00000288899 | rho | 0.21 | 0.06 | -0.43 | -0.29 | -0.62 | -0.50 | -0.47 | -0.15 | 0.47 | 0.38 | 0.18 | -0.15 | -0.21 |
|  |  | p | 0.556 | 0.881 | 0.214 | 0.425 | 0.054 | 0.138 | 0.174 | 0.676 | 0.174 | 0.276 | 0.627 | 0.676 | 0.555 |
| ENSG00000288538 | antisenseKCTD21 | rho | 0.33 | -0.18 | **-0.76** | -0.02 | -0.15 | -0.14 | -0.03 | 0.35 | 0.01 | 0.56 | 0.33 | 0.49 | -0.57 |
|  |  | p | 0.347 | 0.627 | **0.011** | 0.96 | 0.676 | 0.701 | 0.934 | 0.328 | 0.987 | 0.09 | 0.347 | 0.15 | 0.084 |
| ENSG00000288025 | antisenseSCRG1 | rho | 0.14 | -0.01 | -0.38 | -0.32 | -0.08 | 0.25 | -0.10 | 0.09 | 0.35 | **0.75** | **0.64** | 0.52 | -0.55 |
|  |  | p | 0.701 | 0.987 | 0.276 | 0.365 | 0.829 | 0.489 | 0.777 | 0.803 | 0.328 | **0.013** | **0.048** | 0.128 | 0.097 |
| ENSG00000265625 | senseintronicSSH2 | rho | 0.31 | -0.06 | -0.53 | -0.43 | -0.58 | -0.29 | -0.32 | -0.32 | 0.43 | 0.53 | 0.39 | 0.01 | -0.49 |
|  |  | p | 0.385 | 0.881 | 0.117 | 0.214 | 0.082 | 0.425 | 0.365 | 0.365 | 0.214 | 0.117 | 0.26 | 0.987 | 0.154 |
| ENSG00000275056 | senseintronicGLYR1 | rho | 0.44 | -0.13 | **-0.75** | -0.20 | -0.01 | 0.01 | -0.10 | 0.21 | 0.02 | **0.67** | 0.55 | 0.55 | **-0.80** |
|  |  | p | 0.2 | 0.726 | **0.013** | 0.58 | 0.987 | 0.987 | 0.777 | 0.556 | 0.96 | **0.033** | 0.098 | 0.098 | **0.006** |
| ENSG00000284977 | LOC105376244 | rho | 0.61 | -0.13 | **-0.73** | -0.43 | -0.26 | -0.07 | 0.02 | -0.20 | 0.07 | 0.53 | 0.58 | 0.21 | **-0.79** |
|  |  | p | 0.06 | 0.726 | **0.016** | 0.214 | 0.467 | 0.855 | 0.96 | 0.58 | 0.855 | 0.117 | 0.082 | 0.556 | **0.007** |
| ENSG00000271862 | ENSG00000271862 | rho | 0.24 | -0.25 | -0.55 | -0.31 | -0.13 | -0.22 | -0.26 | -0.03 | 0.31 | 0.53 | 0.31 | 0.31 | **-0.74** |
|  |  | p | 0.511 | 0.489 | 0.098 | 0.385 | 0.726 | 0.533 | 0.467 | 0.934 | 0.385 | 0.117 | 0.385 | 0.385 | **0.015** |
| ENSG00000276533 | senseintronicRAB27A | rho | 0.26 | -0.01 | -0.49 | -0.48 | -0.53 | -0.15 | -0.41 | -0.21 | 0.43 | **0.64** | 0.52 | 0.12 | -0.45 |
|  |  | p | 0.467 | 0.987 | 0.15 | 0.162 | 0.117 | 0.676 | 0.244 | 0.556 | 0.214 | **0.048** | 0.128 | 0.751 | 0.192 |
| ENSG00000277687 | senseintronicCACUL1 | rho | 0.21 | 0.13 | -0.42 | -0.31 | -0.56 | -0.31 | -0.35 | -0.14 | 0.55 | 0.54 | 0.36 | -0.01 | -0.30 |
|  |  | p | 0.556 | 0.726 | 0.229 | 0.385 | 0.09 | 0.385 | 0.328 | 0.701 | 0.098 | 0.108 | 0.31 | 0.987 | 0.403 |
| ENSG00000280543 | ASAP1-IT2 | rho | 0.30 | -0.20 | -0.50 | -0.49 | -0.47 | -0.32 | -0.25 | -0.36 | 0.47 | 0.46 | 0.31 | -0.01 | -0.61 |
|  |  | p | 0.405 | 0.58 | 0.138 | 0.15 | 0.174 | 0.365 | 0.489 | 0.31 | 0.174 | 0.187 | 0.385 | 0.987 | 0.062 |
| ENSG00000266900 | senseintronicKIAA1468 | rho | 0.33 | 0.26 | -0.31 | -0.53 | -0.39 | -0.18 | **-0.77** | -0.08 | **0.67** | **0.75** | 0.50 | 0.02 | -0.35 |
|  |  | p | 0.347 | 0.467 | 0.385 | 0.117 | 0.26 | 0.627 | **0.009** | 0.829 | **0.033** | **0.013** | 0.138 | 0.96 | 0.327 |
| ENSG00000290792 | ENSG00000290792 | rho | 0.41 | 0.03 | -0.59 | -0.43 | -0.62 | -0.20 | -0.31 | -0.07 | 0.41 | **0.64** | 0.47 | 0.06 | -0.34 |
|  |  | p | 0.244 | 0.934 | 0.074 | 0.214 | 0.054 | 0.58 | 0.385 | 0.855 | 0.244 | **0.048** | 0.174 | 0.881 | 0.336 |
| ENSG00000289463 | senseintronicZNF638 | rho | 0.09 | 0.33 | -0.41 | -0.07 | -0.38 | -0.14 | -0.30 | -0.01 | 0.36 | 0.60 | 0.54 | 0.25 | -0.30 |
|  |  | p | 0.803 | 0.347 | 0.244 | 0.855 | 0.276 | 0.701 | 0.405 | 0.987 | 0.31 | 0.067 | 0.108 | 0.489 | 0.393 |
| ENSG00000235530 | TMEM11-DT | rho | 0.32 | 0.10 | -0.48 | -0.39 | -0.39 | -0.32 | -0.31 | -0.24 | 0.47 | 0.50 | 0.46 | 0.02 | -0.56 |
|  |  | p | 0.365 | 0.777 | 0.162 | 0.26 | 0.26 | 0.365 | 0.385 | 0.511 | 0.174 | 0.138 | 0.187 | 0.96 | 0.093 |
| ENSG00000289013 | senseintronicSLC8A1 | rho | 0.32 | -0.25 | -0.43 | **-0.70** | **-0.69** | 0.01 | -0.21 | -0.26 | 0.44 | 0.58 | 0.42 | -0.02 | -0.27 |
|  |  | p | 0.365 | 0.489 | 0.214 | **0.025** | **0.029** | 0.987 | 0.556 | 0.467 | 0.2 | 0.082 | 0.229 | 0.96 | 0.444 |
| ENSG00000289251 | ENSG00000289251 | rho | 0.43 | -0.37 | **-0.80** | -0.30 | -0.46 | -0.12 | -0.09 | 0.18 | 0.02 | 0.56 | 0.31 | 0.31 | -0.45 |
|  |  | p | 0.214 | 0.293 | **0.006** | 0.405 | 0.187 | 0.751 | 0.803 | 0.627 | 0.96 | 0.09 | 0.385 | 0.385 | 0.192 |
| ENSG00000271533 | senseintronicFTX | rho | 0.29 | 0.16 | -0.48 | -0.38 | -0.52 | -0.27 | -0.53 | -0.10 | 0.50 | **0.65** | 0.48 | 0.07 | -0.38 |
|  |  | p | 0.425 | 0.651 | 0.162 | 0.276 | 0.128 | 0.446 | 0.117 | 0.777 | 0.138 | **0.043** | 0.162 | 0.855 | 0.275 |
| ENSG00000229989 | MIR181A1HG | rho | 0.22 | -0.09 | -0.46 | -0.49 | -0.53 | 0.04 | -0.16 | -0.18 | 0.41 | **0.65** | 0.54 | 0.19 | -0.39 |
|  |  | p | 0.533 | 0.803 | 0.187 | 0.15 | 0.117 | 0.907 | 0.651 | 0.627 | 0.244 | **0.043** | 0.108 | 0.603 | 0.266 |
| ENSG00000290683 | ENSG00000290683 | rho | 0.20 | 0.18 | -0.22 | -0.49 | -0.18 | 0.02 | -0.27 | -0.12 | **0.71** | **0.69** | 0.58 | 0.18 | -0.52 |
|  |  | p | 0.58 | 0.627 | 0.533 | 0.15 | 0.627 | 0.96 | 0.446 | 0.751 | **0.022** | **0.029** | 0.082 | 0.627 | 0.121 |
| ENSG00000269924 | senseintronicKIAA0146 | rho | 0.27 | -0.27 | -0.55 | -0.38 | **-0.72** | -0.36 | -0.10 | -0.32 | 0.35 | 0.35 | 0.16 | -0.10 | -0.33 |
|  |  | p | 0.446 | 0.446 | 0.098 | 0.276 | **0.019** | 0.31 | 0.777 | 0.365 | 0.328 | 0.328 | 0.651 | 0.777 | 0.345 |
| ENSG00000230539 | AOAH-IT1 | rho | 0.19 | -0.36 | -0.48 | -0.52 | -0.19 | 0.06 | 0.01 | -0.04 | 0.31 | 0.55 | 0.46 | 0.35 | **-0.65** |
|  |  | p | 0.603 | 0.31 | 0.162 | 0.128 | 0.603 | 0.881 | 0.987 | 0.907 | 0.385 | 0.098 | 0.187 | 0.328 | **0.042** |
| ENSG00000236535 | RC3H1-IT1 | rho | 0.36 | -0.10 | -0.43 | **-0.67** | -0.36 | -0.03 | -0.20 | -0.26 | 0.53 | 0.62 | 0.56 | 0.10 | -0.62 |
|  |  | p | 0.31 | 0.777 | 0.214 | **0.033** | 0.31 | 0.934 | 0.58 | 0.467 | 0.117 | 0.054 | 0.09 | 0.777 | 0.056 |
| ENSG00000281026 | N4BP2L2-IT2 | rho | 0.29 | 0.16 | -0.48 | -0.38 | -0.52 | -0.27 | -0.53 | -0.10 | 0.50 | **0.65** | 0.48 | 0.07 | -0.38 |
|  |  | p | 0.425 | 0.651 | 0.162 | 0.276 | 0.128 | 0.446 | 0.117 | 0.777 | 0.138 | **0.043** | 0.162 | 0.855 | 0.275 |
| ENSG00000231607 | DLEU2 | rho | 0.24 | -0.33 | -0.49 | -0.54 | -0.42 | 0.03 | -0.14 | -0.15 | 0.31 | 0.58 | 0.42 | 0.22 | -0.49 |
|  |  | p | 0.511 | 0.347 | 0.15 | 0.108 | 0.229 | 0.934 | 0.701 | 0.676 | 0.385 | 0.082 | 0.229 | 0.533 | 0.148 |
| ENSG00000238113 | LINC01410 | rho | 0.47 | -0.36 | **-0.72** | -0.54 | -0.50 | -0.22 | -0.16 | -0.18 | 0.24 | 0.53 | 0.37 | 0.12 | **-0.63** |
|  |  | p | 0.174 | 0.31 | **0.019** | 0.108 | 0.138 | 0.533 | 0.651 | 0.627 | 0.511 | 0.117 | 0.293 | 0.751 | **0.05** |
| ENSG00000289353 | senseintronicCMTM1_CKLF_CMTM1 | rho | 0.41 | -0.03 | -0.61 | -0.31 | -0.58 | -0.42 | -0.24 | -0.41 | 0.27 | 0.38 | 0.32 | -0.08 | -0.54 |
|  |  | p | 0.244 | 0.934 | 0.06 | 0.385 | 0.082 | 0.229 | 0.511 | 0.244 | 0.446 | 0.276 | 0.365 | 0.829 | 0.111 |
| ENSG00000275764 | ENSG00000275764 | rho | 0.12 | 0.15 | -0.31 | -0.24 | **-0.69** | -0.36 | -0.44 | -0.19 | 0.56 | 0.46 | 0.21 | -0.13 | -0.07 |
|  |  | p | 0.751 | 0.676 | 0.385 | 0.511 | **0.029** | 0.31 | 0.2 | 0.603 | 0.09 | 0.187 | 0.556 | 0.726 | 0.841 |
| ENSG00000274536 | MIR223HG | rho | 0.16 | -0.15 | -0.47 | -0.48 | -0.21 | 0.06 | -0.26 | -0.06 | 0.31 | **0.66** | 0.59 | 0.38 | -0.62 |
|  |  | p | 0.651 | 0.676 | 0.174 | 0.162 | 0.556 | 0.881 | 0.467 | 0.881 | 0.385 | **0.038** | 0.074 | 0.276 | 0.056 |
| ENSG00000242094 | FOXP1-IT1 | rho | 0.24 | -0.16 | -0.53 | -0.54 | -0.50 | -0.04 | -0.30 | -0.09 | 0.35 | **0.65** | 0.53 | 0.21 | -0.44 |
|  |  | p | 0.511 | 0.651 | 0.117 | 0.108 | 0.138 | 0.907 | 0.405 | 0.803 | 0.328 | **0.043** | 0.117 | 0.556 | 0.206 |
| ENSG00000228817 | BACH1-IT2 | rho | 0.22 | -0.24 | -0.43 | -0.53 | -0.47 | 0.06 | -0.13 | -0.21 | 0.38 | 0.59 | 0.44 | 0.18 | -0.43 |
|  |  | p | 0.533 | 0.511 | 0.214 | 0.117 | 0.174 | 0.881 | 0.726 | 0.556 | 0.276 | 0.074 | 0.2 | 0.627 | 0.213 |
| ENSG00000258944 | antisensePAPLN | rho | 0.13 | -0.15 | -0.35 | -0.48 | -0.42 | -0.27 | -0.24 | -0.32 | 0.59 | 0.44 | 0.30 | 0.01 | -0.52 |
|  |  | p | 0.726 | 0.676 | 0.328 | 0.162 | 0.229 | 0.446 | 0.511 | 0.365 | 0.074 | 0.2 | 0.405 | 0.987 | 0.126 |
| ENSG00000270344 | POC1B-AS1 | rho | 0.29 | 0.30 | -0.42 | -0.20 | -0.38 | -0.22 | -0.31 | -0.03 | 0.53 | 0.61 | 0.46 | 0.10 | -0.37 |
|  |  | p | 0.425 | 0.405 | 0.229 | 0.58 | 0.276 | 0.533 | 0.385 | 0.934 | 0.117 | 0.06 | 0.187 | 0.777 | 0.291 |
| ENSG00000272888 | CHASERR | rho | 0.13 | 0.10 | -0.33 | -0.37 | -0.54 | -0.22 | -0.31 | -0.12 | 0.61 | 0.55 | 0.38 | 0.02 | -0.26 |
|  |  | p | 0.726 | 0.777 | 0.347 | 0.293 | 0.108 | 0.533 | 0.385 | 0.751 | 0.06 | 0.098 | 0.276 | 0.96 | 0.476 |
| ENSG00000254325 | ENSG00000254325 | rho | 0.39 | -0.42 | -0.59 | -0.50 | -0.19 | 0.06 | -0.15 | 0.26 | 0.22 | **0.64** | 0.33 | 0.36 | -0.52 |
|  |  | p | 0.26 | 0.229 | 0.074 | 0.138 | 0.603 | 0.881 | 0.676 | 0.467 | 0.533 | **0.048** | 0.347 | 0.31 | 0.121 |
| ENSG00000273691 | senseintronicZNF710 | rho | 0.29 | -0.47 | -0.60 | -0.56 | -0.48 | -0.02 | -0.08 | -0.02 | 0.21 | 0.54 | 0.36 | 0.22 | -0.45 |
|  |  | p | 0.425 | 0.174 | 0.067 | 0.09 | 0.162 | 0.96 | 0.829 | 0.96 | 0.556 | 0.108 | 0.31 | 0.533 | 0.192 |
| ENSG00000229152 | ANKRD10_IT1 | rho | -0.08 | 0.06 | -0.12 | -0.38 | -0.62 | -0.22 | -0.36 | -0.24 | **0.71** | 0.43 | 0.22 | -0.12 | -0.04 |
|  |  | p | 0.829 | 0.881 | 0.751 | 0.276 | 0.054 | 0.533 | 0.31 | 0.511 | **0.022** | 0.214 | 0.533 | 0.751 | 0.907 |
| ENSG00000232973 | CYP1B1-AS1 | rho | 0.47 | -0.02 | **-0.66** | -0.41 | -0.26 | -0.13 | -0.29 | -0.16 | 0.21 | **0.64** | 0.60 | 0.26 | **-0.76** |
|  |  | p | 0.174 | 0.96 | **0.038** | 0.244 | 0.467 | 0.726 | 0.425 | 0.651 | 0.556 | **0.048** | 0.067 | 0.467 | **0.011** |
| ENSG00000228280 | senseintronicC10orf11 | rho | 0.31 | 0.01 | -0.53 | -0.47 | -0.13 | 0.29 | -0.29 | 0.25 | 0.26 | **0.89** | **0.78** | 0.56 | -0.56 |
|  |  | p | 0.385 | 0.987 | 0.117 | 0.174 | 0.726 | 0.425 | 0.425 | 0.489 | 0.467 | **<0.001** | **0.008** | 0.09 | 0.093 |
| ENSG00000261596 | senseintronicMETTL9 | rho | 0.38 | 0.01 | -0.55 | -0.39 | -0.44 | -0.29 | -0.30 | -0.35 | 0.39 | 0.53 | 0.44 | 0.04 | -0.63 |
|  |  | p | 0.276 | 0.987 | 0.098 | 0.26 | 0.2 | 0.425 | 0.405 | 0.328 | 0.26 | 0.117 | 0.2 | 0.907 | 0.053 |
| ENSG00000288839 | senseintronicRNMT | rho | 0.42 | 0.07 | **-0.71** | -0.21 | -0.54 | -0.22 | -0.25 | 0.07 | 0.19 | 0.61 | 0.47 | 0.19 | -0.38 |
|  |  | p | 0.229 | 0.855 | **0.022** | 0.556 | 0.108 | 0.533 | 0.489 | 0.855 | 0.603 | 0.06 | 0.174 | 0.603 | 0.283 |
| ENSG00000287100 | ENSG00000287100 | rho | 0.36 | 0.02 | -0.44 | -0.43 | -0.29 | 0.14 | -0.10 | 0.08 | 0.48 | **0.76** | 0.56 | 0.30 | -0.45 |
|  |  | p | 0.31 | 0.96 | 0.2 | 0.214 | 0.425 | 0.701 | 0.777 | 0.829 | 0.162 | **0.011** | 0.09 | 0.405 | 0.192 |
| ENSG00000236194 | senseintronicSTAT5B | rho | 0.21 | 0.27 | -0.41 | -0.14 | -0.31 | -0.29 | -0.35 | 0.04 | 0.52 | 0.58 | 0.39 | 0.14 | -0.38 |
|  |  | p | 0.556 | 0.446 | 0.244 | 0.701 | 0.385 | 0.425 | 0.328 | 0.907 | 0.128 | 0.082 | 0.26 | 0.701 | 0.275 |
| ENSG00000283839 | LOC105373780 | rho | 0.38 | -0.03 | -0.54 | -0.53 | -0.46 | 0.02 | -0.31 | -0.16 | 0.38 | **0.73** | 0.61 | 0.21 | -0.52 |
|  |  | p | 0.276 | 0.934 | 0.108 | 0.117 | 0.187 | 0.96 | 0.385 | 0.651 | 0.276 | **0.016** | 0.06 | 0.556 | 0.121 |

**Table S8:** Participant characteristics for the larger cohort used in this study to analyze the differences in NOTCH2NLA/B/C RNA expression between women who developed preeclampsia (N=37) and normal pregnant controls (N=215) at index pregnancy in the STORK study.

| **Characteristics** | **Normal pregnant controls (n = 215)** | **Preeclampsia (n = 37)** | **p-value** |
| --- | --- | --- | --- |
|  |  |  |  |
| Age (mean (SD)) | 32 (4) | 30 (4) | 0.005 |
| Primiparous n (%) | 111 (52.1) | 26 (70.3) | 0.062 |
| Sex of the baby: female (%) | 99 (46.3) | 17 (45.9) | 1.000 |
| **BMI (kg/m^2^)*** |  |  |  |
| Weeks 14-16 | 23.5 [21.3, 25.3] | 27.2 [23.2, 29.8] | <0.001 |
| Weeks 22-24 | 24.8 [22.6, 26.8] | 28.9 [24.1, 31.0] | 0.001 |
| Weeks 36-38 | 27.2 [24.9, 29.5] | 31.8 [27.4, 34.8] | <0.001 |
| 5-year follow-up | 22.6 [20.8, 24.5] | 23.0 [20.3, 25.1] | 0.785 |
| **Systolic blood pressure (mmHg)*** |  |  |  |
| Weeks 22-24 | 110 [100, 115] | 114 [105, 120] | 0.069 |
| Weeks 36-38 | 110 [105, 120] | 130 [120, 136] | <0.001 |
| 5-year follow-up | 110 [100, 120] | 110 [106, 110] | 0.568 |
| **Diastolic blood pressure (mmHg)*** |  |  |  |
| Weeks 22-24 | 65 [60, 70] | 70 [60, 80] | 0.002 |
| Weeks 36-38 | 70 [70, 80] | 83 [79, 90] | <0.001 |
| 5-year follow-up | 70 [60, 75] | 70 [63, 74] | 0.753 |
| **Uterine artery PI*** |  |  |  |
| Weeks 22-24 | 0.83 [0.72, 0.99] | 1.01 [0.80, 1.17] | 0.011 |
| Weeks 36-38 | 0.72 [0.60, 0.83] | 0.84 [0.69, 0.98] | 0.013 |
| **sFlt-1 (ng/mL)*** |  |  |  |
| Weeks 22-24 | 1.4 [1.0, 2.0] | 1.8 [1.2, 2.5] | 0.062 |
| Weeks 36-38 | 3.7 [2.5, 5.4] | 6.1 [4.9, 9.0] | <0.001 |
| 5-year follow-up | 0.5 [0.35, 0.8] | 0.4 [0.3, 0.7] | 0.559 |
| **PlGF (pg/mL)*** |  |  |  |
| Weeks 22-24 | 210 [144, 293] | 150 [107, 250] | 0.013 |
| Weeks 36-38 | 134 [61, 264] | 53 [17, 98] | <0.001 |
| 5-year follow-up | 6 [5, 18] | 6 [5, 24] | 0.860 |
| **sFLlt1/PlGF ratio*** |  |  |  |
| Weeks 22-24 | 6.72 [4.51, 11.73] | 10.09 [6.58, 21.88] | 0.002 |
| Weeks 36-38 | 31.33 [11.50, 78.71] | 149.63 [62.47, 716.84] | <0.001 |
| 5-year follow-up | 64.00 [36.80, 116.00] | 59.00 [51.57, 90.71] | 0.802 |

*****(median [25^th^, 75^th^ percentile])

**Table S9: Participant characteristics table for history of preeclampsia** The cohort used to analyze the differences in NOTCH2NLA/B/C RNA expression between women who have had preeclampsia (N=29) or not (N=270) at 5-year follow-up in the STORK study. PE = preeclampsia.

| **Characteristics** | **No previous PE  (N = 271)** | **Previous PE  (N = 29)** | **p-value** |
| --- | --- | --- | --- |
| Age (mean (SD)) | 38 (4) | 38 (4) | 0.730 |
| Sex of the baby: female (%)) | 130 (48.1) | 14 (48.3) | 1.000 |
| **Status at index pregnancy n (%)** |  |  | <0.001 |
| Normal control | 204 (76.4) | 11 (39.3) |  |
| GDM | 63 (23.6) | 7 (25.0) |  |
| PE | 0 (0.0) | 10 (35.7) |  |
| BMI (kg/m^2^)* | 22.9 [21.0, 25.1] | 23.9 [21.1, 29.1] | 0.096 |
| Systolic blood pressure (mmHg)* | 110 [100, 120] | 115 [110, 120] | 0.128 |
| Diastolic blood pressure (mmHg)* | 70 [60, 75] | 70 [65, 80] | 0.048 |
| sFlt-1 (ng/mL)* | 0.5 [0.4, 0.8] | 0.5 [0.3, 1.1] | 0.946 |
| PlGF (pg/mL)* | 6 [5, 19] | 5 [5, 26] | 0.735 |
| **sFLlt1/PlGF ratio*** |  |  |  |
| Weeks 22-24 | 6.4 [4.5, 11.4] | 6.9 [5.5, 14.7] | 0.220 |
| Weeks 36-38 | 29.8 [10.9, 78.7] | 78.6 [25.1, 139.7] | 0.019 |
| 5-year follow-up | 64.0 [36.8, 108.0] | 60.0 [24.5, 98.3] | 0.531 |

*****(median [25^th^, 75^th^ percentile])

**Table S10:** Spearman’s correlation coefficients (rho) and 2-tailed p-values (p-value) for *NOTCH2NLA/B/C* and various clinical variables as well as known markers dysregulated in women with preeclampsia at 22-24 weeks and 36-38 weeks in the total cohort (N=215 normal pregnant controls, N=37 preeclampsia).

| ***NOTCH2NLA/B/C*** | **22-24 weeks** | | **36-38 weeks** | |
| --- | --- | --- | --- | --- |
|  | **rho** | **p-value** | **rho** | **p-value** |
| **Maternal age (years)** | -0.11 | 0.128 | -0.10 | 0.138 |
| **BMI (kg/m^2^)** | -0.11 | 0.115 | -0.06 | 0.393 |
| **Systolic BP (mmHg)** | 0.02 | 0.765 | 0.01 | 0.867 |
| **Diastolic BP (mmHg)** | 0.03 | 0.662 | -0.09 | 0.208 |
| **UtA-PI** | 0.10 | 0.201 | -0.01 | 0.944 |
| **β-galactosidase (ng/µL)** | **-0.15^*^** | 0.032 | **-0.22^**^** | 0.002 |
| **sFlt-1 (ng/mL)** | -0.04 | 0.618 | -0.08 | 0.244 |
| **PlGF (pg/mL)** | -0.06 | 0.407 | 0.06 | 0.363 |
| **sFlt-1/PlGF** | 0.02 | 0.825 | -0.08 | 0.243 |
| **AOPP (µM)** | **-0.15^*^** | 0.032 | -0.04 | 0.525 |

* = Least Significant Differences (LSD)-adjusted p-value < 0.05. ** = LSD-adjusted p-value < 0.01.
